# Understanding preferences for HIV care and treatment in Zambia: Evidence from a discrete choice experiment among patients who have been lost to follow-up

**DOI:** 10.1371/journal.pmed.1002636

**Published:** 2018-08-13

**Authors:** Arianna Zanolini, Kombatende Sikombe, Izukanji Sikazwe, Ingrid Eshun-Wilson, Paul Somwe, Carolyn Bolton Moore, Stephanie M. Topp, Nancy Czaicki, Laura K. Beres, Chanda P. Mwamba, Nancy Padian, Charles B. Holmes, Elvin H. Geng

**Affiliations:** 1 United Kingdom Department for International Development, Dar Es Salaam office, Dar Es Salaam, Tanzania; 2 Centre for Infectious Disease Research in Zambia, Lusaka, Zambia; 3 University of California, San Francisco, San Francisco, California, United States of America; 4 University of Alabama at Birmingham, Birmingham, Alabama, United States of America; 5 James Cook University, Townsville, Australia; 6 Johns Hopkins University, Baltimore, Maryland, United States of America; 7 University of California, Berkeley, Berkeley, California, United States of America; 8 Georgetown University, Washington D.C., United States of America; Massachusetts General Hospital, UNITED STATES

## Abstract

**Background:**

In public health HIV treatment programs in Africa, long-term retention remains a challenge. A number of improvement strategies exist (e.g., bring services closer to home, reduce visit frequency, expand hours of clinic operation, improve provider attitude), but implementers lack data about which to prioritize when resource constraints preclude implementing all. We used a discrete choice experiment (DCE) to quantify preferences for a number of potential clinic improvements to enhance retention.

**Methods and findings:**

We sought a random sample of HIV patients who were lost to follow-up (defined as >90 days late for their last scheduled appointment) from treatment facilities in Lusaka Province, Zambia. Among those contacted, we asked patients to choose between 2 hypothetical clinics in which the following 5 attributes of those facilities were varied: waiting time at the clinic (1, 3, or 5 hours), distance from residence to clinic (5, 10, or 20 km), ART supply given at each refill (1, 3, or 5 months), hours of operation (morning only, morning and afternoon, or morning and Saturday), and staff attitude (“rude” or “nice”). We used mixed-effects logistic regression to estimate relative utility (i.e., preference) for each attribute level. We calculated how much additional waiting time or travel distance patients were willing to accept in order to obtain other desired features of care. Between December 9, 2015 and May 31, 2016, we offered the survey to 385 patients, and 280 participated (average age 35; 60% female). Patients exhibited a strong preference for nice as opposed to rude providers (relative utility of 2.66; 95% CI 1.9–3.42; *p* < 0.001). In a standard willingness to wait or willingness to travel analysis, patients were willing to wait 19 hours more or travel 45 km farther to see nice rather than rude providers. An alternative analysis, in which trade-offs were constrained to values actually posed to patients in the experiment, suggested that patients were willing to accept a facility located 10 km from home (as opposed to 5) that required 5 hours of waiting per visit (as opposed to 1 hour) and that dispensed 3 months of medications (instead of 5) in order to access nice (as opposed to rude) providers. This study was limited by the fact that attributes included in the experiment may not have captured additional important determinants of preference.

**Conclusions:**

In this study, patients were willing to expend considerable time and effort as well as accept substantial inconvenience in order to access providers with a nice attitude. In addition to service delivery redesign (e.g., differentiated service delivery models), current improvement strategies should also prioritize improving provider attitude and promoting patient centeredness—an area of limited policy attention to date.

## Abstract 2

In a discrete choice experiment carried out in Zambia, Elvin Geng and colleagues study the preferences for treatment provision of HIV patients lost to care.

## Author summary

**Why was this study done?:**

- To achieve optimal impact, public health should tailor services to meet, whenever possible, the preferences of populations that could benefit from services.
- Although a global effort to provide treatment for HIV has saved millions of lives, many patients are still inconsistently engaged in care and fail to achieve full health benefits of ART.
- Choice experiments—still a relatively novel tool in public health—can be used to identify preferred features of a health service as well as the relative strength of those preferences in comparison with each other.
- In such an experiment, researchers ask patients to consider a series of comparisons between 2 hypothetical services (e.g., 2 clinics) in which features of that service (e.g., time spent at the facility during a visit) differ. The choices made by a population reveal which features of the services are most desired, as well as how much of another characteristic would they trade for what they desire.

**What did the researchers do and find?:**

- We used such a choice experiment in patients who were lost to follow-up from HIV care. First, we intensively traced patients who were lost to follow-up in the community. Then, we administered a choice survey to those contacted in person who consented.
- In the choice experiment, patients were asked to select between 2 hypothetical facilities that varied in the following 5 attributes: distance from facility to residence (1, 5, or 20 km), time spent waiting at the facility (1, 3, or 5 hours), opening hours (8:00 am–12:00 noon, 8:00 am–3:00 pm, or 8:00 am–12:00 noon plus Saturdays), the quantity of HIV medications dispensed at each visit (1, 3, or 5 months), and the attitude of providers (“rude” or “nice”).
- Overall, 280 patients were surveyed. Patients expressed a strong preference for nice, as opposed to rude, providers, as well as strong preferences for longer versus shorter medication refill duration. As expected, shorter distance, less waiting time, and longer opening hours were also desired.
- A standard willingness to wait analysis suggested that patients would trade up to 19 hours of waiting time to access a facility with nice as opposed to rude providers. A willingness to travel analysis suggested that patients were willing to travel an extra 45 km to see a nice as opposed to a rude provider.
- An alternative approach to quantifying trade-offs avoiding values that fall outside of the range specifically asked in the choice experiment (i.e., the standard willingness to wait suggested a value of 19 hours, exceeding the 5-hour maximum offered in the experiment) suggested that patients were willing to give up 5 months of medications to receive 3 months only, travel 10 km (rather than 5), and spend 3 hours waiting (rather than 1) at a visit, all in order to access a facility with nice providers.

**What do these findings mean?:**

- In addition to current improvement efforts to increase drug dispensation, move services closer to home, and extend hours (in differentiated service delivery models), a concomitant effort to improve healthcare worker attitude has not been undertaken but may represent a high priority.
- International donor agencies as well as national governments responding to the HIV epidemic should consider incorporating training on patient-centered perspectives and communications into investments to build human resources for health.
- Healthcare worker morale and job satisfaction should be systematically assessed and improved.

## Introduction

Although public health programs in Africa continue to rapidly start new patients living with HIV on life-saving treatment programs, sustained engagement of these patients—necessary for long-term success—remains a widespread challenge. Existing delivery practices are often part of the problem: programs to date sometimes expect frequent (e.g., monthly) visits to a healthcare facility for medication refills and clinical review, require standing in long queues once at those facilities, and offer only impersonal and brief interactions with healthcare workers [1]. Although programs recognize the need for improvement, few data exist on the comparative effectiveness of many potential innovations, and therefore the way forward remains uncertain. For example, in order to make treatment more accessible, a clinic could choose to extend open hours, increase the quantity of medications dispensed (and therefore reduce visit frequency), build satellite clinics deeper in the community, or numerous other strategies [2]. In most resource-limited environments, programs cannot implement all strategies simultaneously, and so which one to prioritize remains an important unknown.

Although consensus exists that HIV services must innovate to improve retention and viral suppression, there is less agreement about which innovations to prioritize nor even about a widely usable method for prioritization. Studies of interventions such as short message service (SMS) [3] and peer navigators [4] to improve retention have involved comparisons to “standard of care.” Such studies offer evidence of effectiveness but not the comparative effectiveness needed to choose between 2 (or more) potential innovations. Differentiated service delivery models—which vary the timing, frequency, location, and nature of contact between the health system and patients [5]—are a family of promising approaches widely promoted by stakeholders. But many differentiated service delivery models exist, and some may be more effective or efficient than others. Comparing the numerous possibilities against each other experimentally would be complex, expensive, and time-consuming. Observational evaluations would be less expensive but are often carried out after investments in particular innovations have already been made; therefore, they do not always solve the problem of which combinations of models to invest in to begin with.

In order to inform the prioritization of improvements for retention, we administered a discrete choice experiment (DCE) regarding preferences for clinical services in a random sample of patients who were lost to follow-up from HIV care—a vulnerable group critical to reengage—in Zambia [6]. A choice experiment offers respondents (in this case, patients who were lost to follow-up) a series of attributes of a service (in this case, healthcare) in which attribute characteristics are varied. Examination of responses can reveal a quantitative and comparative measure of the desirability of features of services. These preferences offer insights into the potential attractiveness of certain features of services and thus ones to prioritize. Choice experiments have been used extensively in marketing and increasingly in healthcare, including in Africa [7–9] for HIV-infected pregnant women [10]. To our knowledge, however, they have not yet been used in a general HIV population and among patients who have been lost to follow-up.

## Methods

### Population and sampling

Conceptually, the target population was adult HIV patients who were lost to follow-up but alive in Zambia. The actual source population for selection into the choice experiment were patients who were lost to follow-up (defined as >90 days late for their last visit) in Lusaka province. These patients were identified as a part of a larger parent cohort study to estimate mortality and retention [11]. A random sample of lost patients were intensively sought in the community and traced. Patients who were located in person were offered—in addition to the survey instruments about updated vital and care status—a choice experiment. We obtained ethical review board approvals from the University of Zambia Biomedical Research Ethics Committee (UNZABREC), the University of Alabama, Birmingham, School of Medicine, and the University of California, San Francisco. All patients provided written informed consent to participate in the DCE. The protocol for the choice experiment is available in Supporting information (S1 Appendix).

### Selection of attributes for the choice experiment

**Fig 1:**
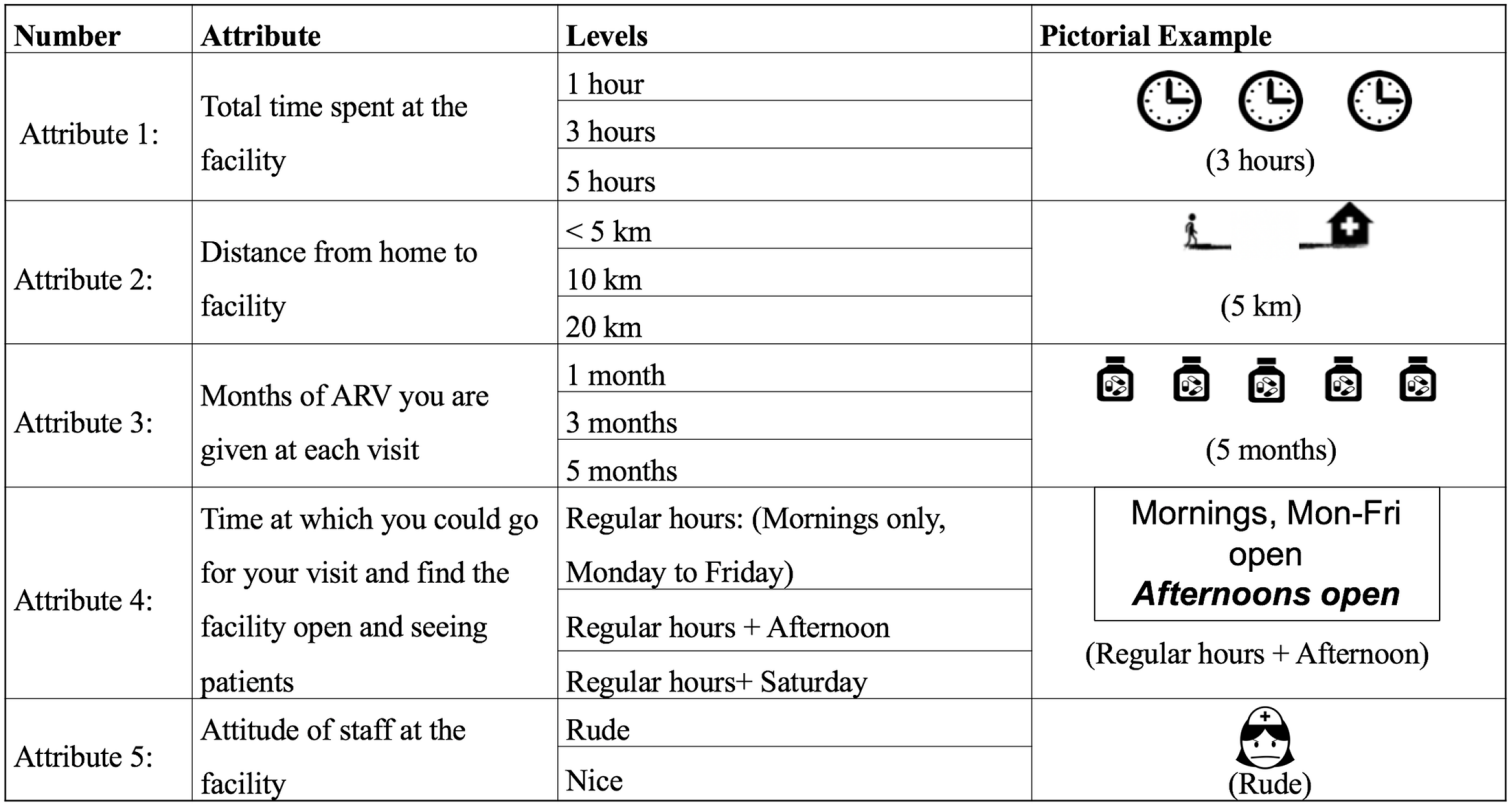
Attributes and levels.

We used qualitative research as well as a literature review to select attributes to include in the DCE [12]. Qualitative interviews were conducted with patients to assess facilitators and barriers to engagement in care in Zambia and are described in a separate manuscript [12]. Emergent themes were discussed with local stakeholders and healthcare workers. In these data sources, long waiting times to see clinicians as well as to collect medications from the pharmacy are widely reported and are therefore included in this study [13,14]. We selected response categories for waiting times of 1, 3, or 5 hours based both on local assessments as well as the literature [14]. Second, emerging data suggest that the quantity of medication dispensed (which determines the frequency of return to the clinic for refills) plays an important role in determining preferences [15]. At present, Zambia’s national policy is to give a 3-month supply of ART drugs to stable HIV treatment patients in line with the World Health Organization, but in practice, 1- or 2-month refills are common [16]. Patients were asked to select between facilities offering 1-, 3-, or 5-month refill intervals. The inability to visit the clinic due to conflicts with work is also widely reported. Although official clinic hours for most facilities in Zambia extend into the afternoon, qualitative interviews revealed that, in practice, sites sometimes do not serve patients after midday. We therefore sought to assess the desirability of clinics serving patients during the usual hours of 8:00 am until 12:00 noon, those with extended hours until 3:00 pm on weekdays, or adding Saturday [17] hours to usual 8:00 am to 12:00 noon hours of operation. Finally, rude, vindictive, and judgmental staff are often cited as a barrier to care and were mentioned by patients in the qualitative interviews [10,18]. Our choice experiment therefore included either “rude” or “nice” as characteristics of healthcare workers in facilities of interest. Finally, distance from residence to clinic is another common barrier to care, and therefore it was also included. The distances offered in the experiment were 5, 10, or 20 km between residence and clinic. All attributes were accompanied by pictures to help with the cognitive task of summarizing attributes at each clinic and comparing 2 clinics (Fig 1).

### DCE design

We followed standard approaches for the design of the choice experiment in order to achieve unbiased as well as statistical and response efficiency [19]. The DCE was based on 5 attributes. Four of the 5 attributes were described in choice tasks by 3 response levels and one attribute by 2 levels, yielding a total of (e.g., 3 × 3 × 3 × 3 × 2) 162 potential combinations. Because the total number of comparisons ([162 × 161]/2 or 13,041 total combinations) cannot be feasibly evaluated, we developed a fractional factorial design to minimize the total number of choice sets [19] using the method of Street and colleagues [20,21]. We used an orthogonal main effects plan (OMEP) in which we restricted comparisons to main effects across attribute levels but did not seek to compare all attribute levels across all possible combinations of other attributes [19]. Using provider attitude and a 2-level waiting time attribute as a hypothetical example, this would compare a clinic with a “rude provider” and “short visit” to another with “nice providers” and “long visits” but would not seek to make this comparison across all variations in all other attributes. This approach reduces the potential combinations substantially but sacrifices the ability to discern differences in preferences by levels of another attribute (i.e., effect modification). In this design, attribute levels were balanced (all attribute levels appear equally often in the experiment) and orthogonal (each pair of attribute levels appeared with the same frequency across all attribute pairs) [19]. Based on this design and recommendations in the literature [22], we assumed that more than 9 choice tasks would represent the maximum cognitive and time burden for participants, and so we used 18 choice sets in 2 blocks of 9 questions each. We included an opt-out response category in which a respondent could choose neither clinic in order to reflect potential disengagement of respondents if the service model was presented in the real world. The final design was fully efficient with a D-efficiency of 100% compared with the optimal design (S1 Table) [21]. We included a dominant response—that is, one in which one clinic was obviously preferable to the other because all attributes were more desirable. This question was used to evaluate how well respondents understood the choice experiment.

### Sample size

We aimed for an ideal sample size of 500 patients based on previous literature regarding sample size estimation for DCEs [19] but planned from the onset to modify the total sample size given actual logistical constraints that could emerge during the study. To seek unbiased—even if less precise—estimates if we were unable to trace all lost patients targeted for the choice experiment, we identified subjects randomly over the time period from a list of patients who were lost to follow-up. Given timing and competing priorities, we decided to end recruitment at 280 individuals. The rationale for doing so was in part due to calculations that show that the marginal increase in power between 300 (our approximate final sample size) and 500 (our ideal sample size) was minimal. The decision to stop recruitment was made in advance of any analysis of the data.

### Data collection

The choice experiment was delivered on tablets. The module was available in English as well as in all of the major local languages, and the patients could choose their preferred language. After looking for a private or protected setting, research assistants were trained to share the tablet screen with the respondent so that the respondent could visually see the 2 options. The research assistant would then walk them through each option using the following language: “In Clinic [A], the total time you spend at the clinic is [B] hours. The clinic is [C] km away from your home. At each visit, you are given [D] months of ART. The clinic sees patients [F]. The staff at the clinic is [G]”. Research assistants point at the corresponding images as they explain each attribute. The standard operating procedures for data collection are available in Supporting information (S2 Appendix).

### Analysis

We used STATA version 14 to clean and analyze the data. Descriptive characteristics were tabulated. We used a mixed logit regression model to estimate the relative utility (i.e., preference) of each attribute level in this patient population [23]. We assumed an independent covariance structure. In our main analysis, we treated distance as continuous, refill intervals as a multilevel ordinal variable (such that 3 months of drug dispensation is compared to 1 month, and 5 months is compared to 3 months), hours of waiting at the facility as continuous, hours of operation as a multilevel ordinal variable and staff attitude as binary. We introduced random effects for each term in the regression to allow heterogeneity in patient responses, except for waiting time, which we treated as a fixed effect (in order to allow our main willingness to wait analysis). We calculated McFaddens Psuedo-R^2^ (1 − [e(ll)/e(ll_0)]) to evaluate goodness of fit of mixed logit models [23]. We calculated a willingness to wait for each variable, which allows us to standardize the relative utility derived from visiting a clinic with a given characteristic against waiting time, similar to a “willingness to pay” analysis [24]. To calculate willingness to wait, we divided the coefficient of each variable by the coefficient of waiting time (thus considering waiting time linear). To explore relative strengths of preferences, we similarly calculated willingness to travel by dividing each coefficient by the coefficient for distance from a regression model in which travel time was treated as a fixed effect and all other attributes were treated as random effects. Finally, we explored an alternative approach to assess the strength of preferences to avoid assumption of linearity in the standard willingness to wait analyses—an assumption that can lead to trade-off values outside of the ranges in an attribute actually presented to the participants. To do so, we treated distance and time as ordinal in the regression model and summed regression coefficients for a number of attributes to reach the value of an alternative. We conducted subgroup analyses by sex, ART status, updated care status, and healthcare setting to explore differences in the preferences within these subgroups.

We followed the ISPOR Good Research Practices for Conjoint Analysis Task Force guidelines for Conjoint Analysis Applications in Health, a checklist (S3 Appendix).

## Results

**Fig 2:**
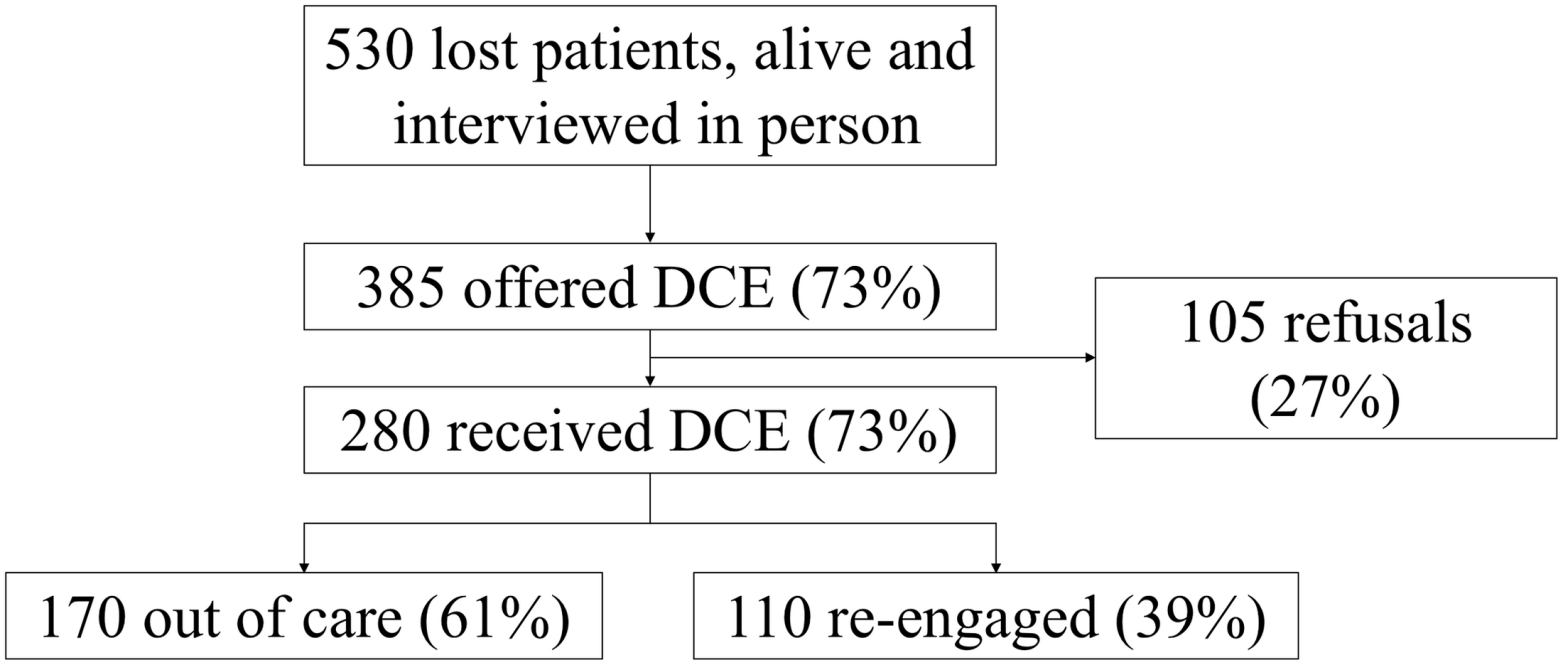
Flow diagram of patient selection for DCE. DCE, discrete choice experiment.

The choice experiment was offered to a random sample of 385 of 530 persons lost to follow-up identified by a parent study. Of the 385, 105 did not consent, yielding 280 people who were lost, traced, and contacted in the field and who agreed to the experiment between December 9, 2015 and May 31, 2016 (Fig 2). The 280 who received the DCE were very similar to the 105 who refused the DCE on clinical, sociodemographic, and other measures (S2 Table). Of the 280 respondents, the average age was 35 years, 60% were females, and 60% were married. A total of 55% were on ART at the time of loss to follow-up; most (68%) reported having initiated ART. The average time since the last visit at the original clinic was 1.7 years. Overall, 170 (61%) of these lost patients were not in care, and 110 (39%) had reconnected to care either at a new facility or back at their original facility by the end of the experiment.

Each patient was given 9 choice tasks that were drawn from 2 sets, resulting in 2,520 choices presented to 280 persons. Only 18 participants selected the opt-out response (neither clinic) for a given choice task, with 1 patient choosing neither clinic for all responses. Due to the limited number of opt-out responses, these were treated as missing in further analyses. Ten participants did not complete all questions presented in their choice set, indicating response fatigue for the last few questions in each choice set (S3 Table). Overall 2,489 choices from 279 participants contributed the main effects analysis, in which 92% of participants responded to the dominant question as expected.

**Fig 3:**
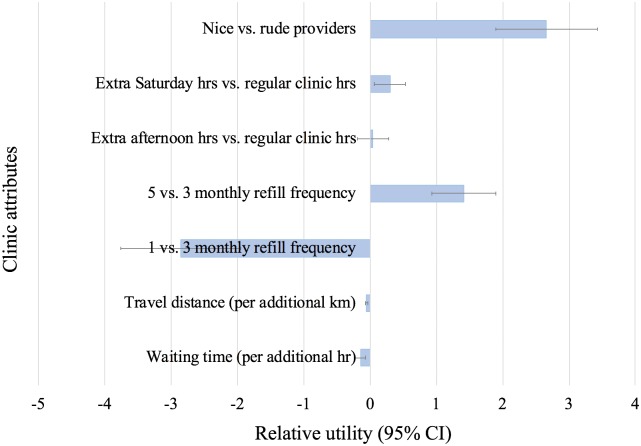
Relative utilities of clinic attributes (mixed logit regression model).

Results from the mixed logit model (Fig 3, Table 1) show that respondents held strong preferences for nice providers as opposed to rude providers (β or relative utility = 2.66; 95% CI 1.90–3.42; *p* < 0.001). Patients also exhibited strong preferences for receiving 5 months of medication compared with 3 months (β = 1.42; 95% CI 0.93–1.90; *p* < 0.001), as well as 3 months compared with 1 month (β = −2.85; 95% CI −3.76 to −1.95; *p* < 0.001). Patients expressed a preference for facilities that were nearer to their residence (β = −0.05 per km; 95% CI −0.07 to −0.03; *p* < 0.001), as well as reduced waiting time (β = −0.14; 95% CI −0.22 to −0.06; *p* < 0.001; per hour). Extending to weekend hours solicited only signs of a mild preference (β = 0.30; 95% CI 0.07–0.54; *p* = 0.011). There was no discernable preference with regard to extended afternoon clinic hours in addition to mornings (β = 0.04; 95% CI −0.19 to 0.28; *p* = 0.713).

**Table 1:** Mixed logit regression model results and willingness to wait analysis (*N* = 280).

Clinic attribute	β	95% CI		*p*-Value	Willingness to wait		
					Hours	95% CI	
Waiting time (per additional h)	−0.14	−0.22	−0.06	<0.001	-	-	-
Travel distance (per additional km)	−0.05	−0.07	−0.03	<0.001	0.37	0.15	0.58
1 versus 3 monthly refill frequency	−2.85	−3.76	−1.95	<0.001	19.94	7.60	32.28
5 versus 3 monthly refill frequency	1.42	0.93	1.90	<0.001	−9.90	−16.35	−3.44
Extra afternoon hours versus regular clinic hours	0.04	−0.19	0.28	0.713	−0.31	−2.01	1.39
Extra Saturday hours versus regular clinic hours	0.30	0.07	0.54	0.011	−2.12	−4.02	−0.22
Nice versus rude providers	2.66	1.90	3.42	<0.001	−18.59	−29.15	−8.03
Constant	0.52	−0.22	1.27	0.17	-	-	-
Model specifications	Log likelihood = −836.973; Prob > chi-squared = 0.000; Wald chi-squared (10) = 158.66; McFadden psuedo R^2^ = 0.35						

β = β-coefficient and represents relative utility; positive values represent positive preference. Mixed logit regression model with waiting time as a fixed effect and other attributes as random effects.

The willingness to wait analysis, carried out in order to calibrate the strength of patient preferences to a single standard, quantified how much waiting time patients were willing to tolerate in order to obtain a desired level of an attribute. This analysis revealed that patients were willing to wait 22 additional minutes (95% CI 9–35 minutes) to attend an otherwise equal facility that was 1 km closer to their residence. Similarly, patients were willing to wait about 20 hours at an otherwise equal facility giving 3 months of ART rather than only 1 month (19.94 hours; 95% CI 7.6–32.28 hours) and were willing to wait around 10 hours to obtain 5 months of medications compared with 3 months (−9.9 hours; 95% CI −16.35 to −3.44 hours). Patients were willing to wait nearly an additional 19 hours to use a facility with nice rather than rude staff (−18.59 hours; 95% CI −29.15 to −8.03 hours), all other things being equal.

In an alternative analysis to quantify the strength of preferences given large values in the standard willingness to wait analysis, we explored alternative approaches. First, we determined willingness to travel (S4 Table). In this analysis, we found that patients were willing to travel almost an additional 45 km for access to nice providers (−44.94 km; 95% CI −62.12 to −27.75 km). Second, we used a model in which waiting time and travel distance were treated as multilevel ordinal variables that we used to quantify trade-offs that would not exceed the response values actually offered for each attribute (Table 2). This analysis suggested that, in order to obtain nice staff, patients were willing to give up a facility that offered 5 as opposed to 3 months of medications, was located 10 km versus 5 km from home, and at which they needed to spend 5 as opposed to 1 hour waiting during a clinic visit (β difference = −0.03 [95% CI −1.05 to 1.00]).

**Table 2:** Mixed logit regression model with ordinal attributes (*N* = 280).

Clinic attribute	β	95% CI		*p*-Value
Waiting time 3 h versus 1 h	−0.27	−0.56	0.14	0.062
Waiting time 5 h versus 1 h	−0.59	−0.91	−0.28	<0.001
Travel distance 10 km versus 5 km	−0.44	−0.66	−0.22	<0.001
Travel distance 20 km versus 5 km	−0.80	−1.09	−0.52	<0.001
1 versus 3 monthly refill frequency	−3.10	−3.78	−2.42	<0.001
5 versus 3 monthly refill frequency	1.37	0.89	1.85	<0.001
Extra evening hours versus regular clinic hours	0.00	−0.24	0.24	0.987
Extra Saturday hours versus regular clinic hours	0.25	0.04	0.46	0.018
Nice versus rude providers	2.5	1.8	3.2	<0.001
Constant	0.8	0.1	1.5	0.019
Model specifications	Log likelihood = −836.782; Prob > chi-squared = 0.000; Wald chi-squared (10) = 180.74; McFadden Psuedo R^2^ = 0.305			

β = β-coefficient and represents relative utility; positive values represent positive preference. Mixed logit regression model with all attributes modelled as ordinal variables.

Overall, there was little difference in preferences between subgroups of participants. We conducted subgroup analyses by sex (S5 Table), ART status (S6 Table), and care status (S7 Table). These analyses showed similar results to the full cohort, with strong preferences for nicer staff and longer refills but with some loss of precision around estimates due to the reduced number of participants. A subgroup analysis performed to determine differences between patients attending rural, urban, or hospital-based clinics (S8 Table) yielded similar results to the main analysis for urban participants; we could not draw firm conclusions about the preferences of those attending other facilities due to the limited number of participants in these groups. A further sensitivity analysis restricted to participants who responded to all questions in their choice set (*N* = 262) showed little to no difference in parameter estimates compared with the full cohort (S9 Table).

## Discussion

Among HIV-infected persons who were lost to follow-up in Zambia, a choice experiment provided clear insights about what characteristics of care patients prefer—thus offering immediately actionable information for programs seeking to address the widespread predicament of poor retention. Nice providers emerged as a characteristic of unexpected importance. In a standard willingness to wait analysis, patients were willing to trade 19 hours of waiting time to obtain nice providers. Given the fact that 19 hours exceeded the actual waiting time categories offered in the choice experiment and was a product of a linearity assumption standard in willingness-to-wait analyses, we explored alternative ways of quantifying trade-offs. We attempted a willingness to travel analysis, which suggested that patients would travel 45 km farther to access a facility with nice as opposed to rude providers. An even more conservative additional approach, in which trade-offs were not allowed to exceed values actually asked, found that patients would be willing to accept a facility that required a combination of 5 hours of waiting time (as opposed to 1), was located 10 km from home (as opposed to 5 km), and that gave a 3 (as opposed to 5)-month supply of medications, all in order to access a facility staffed with nice as opposed to rude providers. Although each method offers slightly different ways of quantifying trade-offs, the message is clear and consistent: healthcare worker attitude is critically important to patients. Other salient findings included quantifying willingness to wait or travel in order to access facilities that gave larger quantities of medication, were located nearer to their residence, and had operating hours on Saturdays.

These findings concur with motivations that underlie the ongoing dissemination of differentiated service delivery models [5]. The strong preference for dispensation of more drugs, and therefore longer intervals between visits, underscores the fact that that even though the medications are free, opportunity costs of travel to clinics for medication refills and scheduled patient review are formidable [25,26]. Many patients engaged in hourly wage labor must give up a day of income to travel to facilities for HIV care. Caregivers for children often must travel with dependents, creating additional expenses and difficulties. Finally, many patients experience unexpected social obligations (such as weddings or burials) that conflict with frequent clinic visits. Studies showing an association between longer visit intervals and a higher probability of making the visit and reduced risk of loss to follow-up complement the findings in this analysis as well as demonstrate concordance between patient report (or stated preferences) with evidence of behaviors (or revealed preference) [15].

The results of this choice experiment, however, go beyond supporting existing directions. Indeed, the single biggest message points to new directions for public health innovations that have, to date, received relatively little attention: improving healthcare worker morale, attitude, and communication skills. Despite the widespread challenges to transportation and opportunity costs faced by African patients, they were willing to trade strikingly large amounts of time, effort, or inconvenience to interact with healthcare workers who were nice. In industrialized settings, interactions between healthcare workers and patients have been shown to influence a range of health outcomes in cardiovascular and metabolic as well as in HIV care [27–29]. In Africa, some may have assumed that unpleasant interactions with healthcare workers would represent only a minor challenge in the context of diverse structural barriers to care. This appears to be untrue. Recent innovative work by Kruk and colleagues showed that respect was highly valued among pregnant women with HIV in Africa [9,10,30–32]. Improving provider attitude, however, also depends on addressing myriad challenges faced by front-line healthcare workers in frail health systems. Studies suggest that burn out, inadequate remuneration, limited opportunities for career advancement, and poor working environments are all common. In sum, while this study supports continued expansion of existing models to make services easier to access (e.g., differentiated service delivery), stakeholders should also consider a commensurate investment to understand, train, and mentor healthcare workers to improve patient–provider interactions.

Patients who receive HIV care resemble, in many ways, all other consumers. Established methods, therefore, for evaluating consumer preferences can and should be more widely used to augur demand in this population and tailor services to meet that demand. Although qualitative studies uncover important understandings of the patient experience, they do not quantify specific preferences for characteristics of healthcare. Prospective experiments can be costly and time-consuming to conduct. The choice experiment can be undertaken relatively rapidly, offers quantitative information, and therefore can provide critical and useful data. Choice experiments have been used more extensively in North America and Europe, where the conceptualization of patient as consumer is long-standing. More extensive use of choice experiments can bring patient perspectives and preferences front and center in the conversation about improving long-term retention in care in Africa.

This study has limitations. First, DCEs are based on stated preferences and not on actual behaviors. The existing literature suggests that response in choice experiments predicts behavior, but this association is far from perfect. As a result, while choice experiments can efficiently narrow the field of candidate interventions, evidence of patient behavior may still be needed in many circumstances. A second limitation in this analysis stems from the fact that a choice experiment does not offer the universe of attributes because the choice task becomes difficult and patients are less willing to critically appraise each attribute as the list grows. Not all potentially important attributes, such as psychosocial factors, were assessed. Third, our willingness to wait analysis was based on the ratio of 2 regression coefficients, which assumes linearity in trade-off relationships, an assumption which may not be met. We therefore conducted additional analyses to capture the willingness to travel as well as summarizing preferences without extrapolating beyond the actual responses assessed. Fourth, given the limited sample size, we were unable to examine whether preferences differed between subgroups of interest. Specifically, there were few patients from rural sites, and our results may therefore be more generalizable to patients in urban settings. Finally, our finding that extending hours into the afternoon was not highly valued could have been influenced by the fact that we carried out interviews during working hours, which means that the patients contacted could have been those who were not working at that time and were therefore less likely to need extended operating hours.

In summary, we conducted a choice experiment to assess patient preferences for different elements of the health system among a group of vulnerable HIV patients who had failed to remain engaged. Their responses suggest clear priorities for health systems innovations. Differential service delivery models to reduce the logistical burden of accessing care may achieve optimal impact if combined with interventions to improve healthcare worker empathy and attitude. Interventions to optimize provider attitude have yet to figure prominently into efforts to improve global HIV treatment but should be explored. Wider use of choice experiments can help improve prioritization of innovations of public health services when resource constraints preclude use of all strategies.

## Supporting information

S1 Appendix Protocol. (PDF)

S2 Appendix Standard operating procedures. (PDF)

S3 Appendix Checklist for conjoint analyses methods and reporting. (DOCX)

S4 Appendix Study data. (XLSX)

S1 Table Results of evaluation of DCE statistical efficiency. DCE, discrete choice experiment.
(DOCX)

S2 Table Patient characteristics. (DOCX)

S3 Table Patient responses to choice sets. (DOCX)

S4 Table Mixed logit model and willingness to travel analysis. (DOCX)

S5 Table Mixed logit model and willingness to wait, by gender. (DOCX)

S6 Table Mixed logit model, restricted to ART users. (DOCX)

S7 Table Mixed logit model, by engagement status. (DOCX)

S8 Table Mixed logit model, by healthcare setting. (DOCX)

S9 Table Mixed logit model, restricted to complete choice sets. (DOCX)

## Abbreviations

DCE: discrete choice experiment
OMEP: orthogonal main effects plan
SMS: short message service
UNZABREC: University of Zambia Biomedical Research Ethics Committee

## References

[1] Amanyire G, Wanyenze R, Alamo S, Kwarisiima D, Sunday P, Sebikaari G, et al Client and provider perspectives of the efficiency and quality of care in the context of rapid scale-up of antiretroviral therapy. AIDS Patient Care STDS. 2010;24(11):719–27. 10.1089/apc.2010.0108 21034243 PMC2994592

[2] Bemelmans M, Baert S, Goemaere E, Wilkinson L, Vandendyck M, Cutsem G, et al Community-supported models of care for people on HIV treatment in sub-Saharan Africa. Trop Med Int Health. 2014;19(8):968–77. 10.1111/tmi.12332 24889337

[3] Pop-Eleches C, Thirumurthy H, Habyarimana JP, Zivin JG, Goldstein MP, de Walque D, et al Mobile phone technologies improve adherence to antiretroviral treatment in a resource-limited setting: a randomized controlled trial of text message reminders. AIDS. 2011;25(6):825–34. 10.1097/QAD.0b013e32834380c1 21252632 PMC3718389

[4] Chang LW, Kagaayi J, Nakigozi G, Ssempijja V, Packer AH, Serwadda D, et al Effect of peer health workers on AIDS care in Rakai, Uganda: a cluster-randomized trial. PLoS ONE. 2010;5(6):e10923 10.1371/journal.pone.0010923 20532194 PMC2880005

[5] Grimsrud A, Bygrave H, Doherty M, Ehrenkranz P, Ellman T, Ferris R, et al Reimagining HIV service delivery: the role of differentiated care from prevention to suppression. J Int AIDS Soc. 2016;19(1):21484 10.7448/IAS.19.1.21484 27914186 PMC5136137

[6] Ryan M, Gerard K, Amaya-Amaya M. Using discrete choice experiments to value health and health care: Springer Science & Business Media; 2007.

[7] Terris-Prestholt F, Hanson K, MacPhail C, Vickerman P, Rees H, Watts C. How much demand for new HIV prevention technologies can we really expect? Results from a discrete choice experiment in South Africa. PLoS ONE. 2013;8(12):e83193 10.1371/journal.pone.0083193 24386160 PMC3875434

[8] Mangham LJ, Hanson K, McPake B. How to do (or not to do)… Designing a discrete choice experiment for application in a low-income country. Health Policy Plan. 2009;24(2):151–8. 10.1093/heapol/czn047 19112071

[9] Kruk ME, Mbaruku G, McCord CW, Moran M, Rockers PC, Galea S. Bypassing primary care facilities for childbirth: a population-based study in rural Tanzania. Health Policy Plan 2009;24(4):279–88. 10.1093/heapol/czp011 19304785

[10] Kruk ME, Riley PL, Palma AM, Adhikari S, Ahoua L, Arnaldo C, et al How can the health system retain women in HIV treatment for a lifetime? A discrete choice experiment in Ethiopia and Mozambique. PLoS ONE. 2016;11(8):e0160764 10.1371/journal.pone.0160764 27551785 PMC4994936

[11] Holmes CB, Sikazwe I, Sikombe K, Eshun-Wilson I, Czaicki N, Beres LK, et al Estimated mortality on HIV treatment among active patients and patients lost to follow-up in 4 provinces of Zambia: Findings from a multistage sampling-based survey. PLoS Med. 2018;15(1):e1002489 10.1371/journal.pmed.1002489 29329301 PMC5766235

[12] Topp SM, Mwamba C, Sharma A, Mukamba N, Beres LK, Geng E, et al Rethinking retention: Mapping interactions between multiple factors that influence long-term engagement in HIV care. PLoS ONE. 2018;13(3):e0193641 10.1371/journal.pone.0193641 29538443 PMC5851576

[13] Dahab M, Charalambous S, Hamilton R, Fielding K, Kielmann K, Churchyard GJ, et al "That is why I stopped the ART": patients’ & providers’ perspectives on barriers to and enablers of HIV treatment adherence in a South African workplace programme. BMC Public Health. 2008;8:63 10.1186/1471-2458-8-63 18282286 PMC2276211

[14] Wanyenze RK, Wagner G, Alamo S, Amanyire G, Ouma J, Kwarisima D, et al Evaluation of the efficiency of patient flow at three HIV clinics in Uganda. AIDS Patient Care STDS. 2010;24(7):441–6. 10.1089/apc.2009.0328 20578908 PMC2933556

[15] Mody A, Roy M, Sikombe K, Savory T, Holmes C, Bolton-Moore C, et al Improved Retention With 6-Month Clinic Return Intervals for Stable Human Immunodeficiency Virus-Infected Patients in Zambia. Clin Infect Dis. 2018;66(2):237–43. 10.1093/cid/cix756 29020295 PMC5850531

[16] McCarthy EA, Subramaniam HL, Prust ML, Prescott MR, Mpasela F, Mwango A, et al Quality improvement intervention to increase adherence to ART prescription policy at HIV treatment clinics in Lusaka, Zambia: A cluster randomized trial. PLoS ONE. 2017;12(4):e0175534 10.1371/journal.pone.0175534 28419106 PMC5395211

[17] Bogart LM, Chetty S, Giddy J, Sypek A, Sticklor L, Walensky RP, et al Barriers to care among people living with HIV in South Africa: contrasts between patient and healthcare provider perspectives. AIDS Care. 2013;25(7):843–53. 10.1080/09540121.2012.729808 23061894 PMC3552028

[18] Sando D, Kendall T, Lyatuu G, Ratcliffe H, McDonald K, Mwanyika-Sando M, et al Disrespect and abuse during childbirth in Tanzania: are women living with HIV more vulnerable? J Acquir Immune Defic Syndr. 2014;67(Suppl 4):S228. 25436822 10.1097/QAI.0000000000000378 PMC4251905

[19] Johnson FR, Lancsar E, Marshall D, Kilambi V, Mühlbacher A, Regier DA, et al Constructing experimental designs for discrete-choice experiments: report of the ISPOR conjoint analysis experimental design good research practices task force. Value Health. 2013;16(1):3–13. 10.1016/j.jval.2012.08.2223 23337210

[20] Street DJ, Burgess L, Louviere JJ. Quick and easy choice sets: Constructing optimal and nearly optimal stated choice experiments. International Journal of Research in Marketing. 2005;22(4):459–70.

[21] Street DJ. Sydney: School of Mathematical Sciences UoT. Discrete Choice Experiments 2007 [Computer software]. http://crsu.science.uts.edu.au/choice. [Accessed 13 Nov 2015].

[22] Bridges JF, Hauber AB, Marshall D, Lloyd A, Prosser LA, Regier DA, et al Conjoint analysis applications in health—a checklist: a report of the ISPOR Good Research Practices for Conjoint Analysis Task Force. Value Health. 2011;14(4):403–13. 10.1016/j.jval.2010.11.013 21669364

[23] Hauber AB, Gonzalez JM, Groothuis-Oudshoorn CG, Prior T, Marshall DA, Cunningham C, et al Statistical Methods for the Analysis of Discrete Choice Experiments: A Report of the ISPOR Conjoint Analysis Good Research Practices Task Force. Value Health. 2016;19(4):300–15. 10.1016/j.jval.2016.04.004 27325321

[24] Kleinman L, McIntosh E, Ryan M, Schmier J, Crawley J, Locke GR, et al Willingness to pay for complete symptom relief of gastroesophageal reflux disease. Arch Intern Med. 2002;162:1361–6. 12076234 10.1001/archinte.162.12.1361

[25] Layer EH, Brahmbhatt H, Beckham SW, Ntogwisangu J, Mwampashi A, Davis WW, et al “I pray that they accept me without scolding:” Experiences with disengagement and re-engagement in HIV care and treatment services in Tanzania. AIDS Patient Care STDS. 2014;28(9):483–8. 10.1089/apc.2014.0077 25093247

[26] Dlamini-Simelane TT, Moyer E. ‘Lost to follow up’: rethinking delayed and interrupted HIV treatment among married Swazi women. Health Policy Plan. 2016;32(2):248–56. 10.1093/heapol/czw117 28207052

[27] Beach MC, Keruly J, Moore RD. Is the quality of the patient-provider relationship associated with better adherence and health outcomes for patients with HIV? J Gen Intern Med. 2006;21(6):661–5. 10.1111/j.1525-1497.2006.00399.x 16808754 PMC1924639

[28] Stewart M, Brown JB, Donner A, McWhinney IR, Oates J, Weston WW, et al The impact of patient-centered care on outcomes. J Fam Pract. 2000;49(9):796–804. 11032203

[29] Stewart MA. Effective physician-patient communication and health outcomes: a review. CMAJ. 1995;152(9):1423–33. 7728691 PMC1337906

[30] Kruk ME, Johnson JC, Gyakobo M, Agyei-Baffour P, Asabir K, Kotha SR, et al Rural practice preferences among medical students in Ghana: a discrete choice experiment. Bull World Health Organ. 2010;88(5):333–41. 10.2471/BLT.09.072892 20458371 PMC2865662

[31] Kruk ME, Paczkowski M, Mbaruku G, de Pinho H, Galea S. Women’s preferences for place of delivery in rural Tanzania: a population-based discrete choice experiment. Am J Public Health. 2009;99(9):1666 10.2105/AJPH.2008.146209 19608959 PMC2724466

[32] Kruk ME, Paczkowski MM, Tegegn A, Tessema F, Hadley C, Asefa M, et al Women’s preferences for obstetric care in rural Ethiopia: a population-based discrete choice experiment in a region with low rates of facility delivery. J Epidemiol Community Health. 2010;64(11):984–8. 10.1136/jech.2009.087973 19822558

