# Supplementary material for: Understanding preferences for HIV care and treatment in Zambia: Evidence from a discrete choice experiment among patients who have been lost to follow-up
Source: PLoS Med. 2018 Aug 13;15(8):e1002636. doi: 10.1371/journal.pmed.1002636 (PMC6089406; doi:10.1371/journal.pmed.1002636)
Supplement: S1 Appendix — (PDF) [file pmed.1002636.s001.pdf]

Better Information for Health in Zambia:

Evaluating Outcomes of HIV Care and Treatment Across Four Provinces

(BetterInfo)

Protocol Version 1.3

15 May 2015

Sponsor: The Bill and Melinda Gates Foundation

Table of Contents

1.0 Background and Introduction 4

1.1 Rationale for study 4

1.2 Significance 5

1.3 Aim 6

1.4 Specific Objectives 7

1.5 Ethical Issues 8

2.0 Literature Review 9

2.1 Results of HIV-infected patients in Africa after engaging care is not well documented 9

2.2 Current literature suggests that the fate of lost to follow-up patients is heterogeneous and varies from setting to setting 9

2.3 Studies based on modelling outcomes among lost to follow-up patients have limitations 9

2.4 A well-established literature in survey epidemiology informs an efficient approach to assessing outcomes in HIV programs 10

2.5 A better understanding of health outcomes leads to a better understanding of effectiveness of health services 10

3.0 Methodology 10

3.2 Objective 2: 'Determine drivers of engagement in care through study of patient perceptions, health care worker experiences, and facility-level factors' Research Methods 26

3.3 Objective 3 'Identify epidemiologic predictors of outcomes' 33

Research Methods 33

3.4 Objective 4 'Understand the process of implementing a sampling-based approach to address lost to follow-up' 36

Research Methods 36

Process and Outcome Results Dissemination 41

3.5 Considerations for Human Participants 41

3.5.2.1 Physical risks 42

3.5.2.2 Social risks 42

3.5.2.3 Psychological 42

3.6 Data Usage ..... **Error! Bookmark not defined.**

4.0 Budget 44

5.0 Time Frame 44

6.0 Reference List 46

Roster of Investigators

Name, Affiliation	Role
Dr. Charles Holmes, MD, MPH Centre for Infectious Disease Research in Zambia (CIDRZ) and John Hopkins University.	Principal Investigator
Dr. Elvin Geng, MD, MPH University of California San Francisco	Co-Principal Investigator
Dr. Izukanji Sikazwe, MBChB, CIDRZ	CIDRZ Principal Investigator
Dr. Muntanga Mapani, MBChB, CIDRZ	Co-Investigator
Dr. Steph Topp, PhD, MPH, MPhil, University of Alabama at Birmingham	Co-Investigator
Laura Beres, MPH, CIDRZ	Co-Investigator
Dr. Carolyn Bolton, MBChB, MSc, University of North Carolina at Chapel Hill (UNC)	Co-Investigator, UNC Principal Investigator
Dr. Nancy Padian, PhD, MPH University of California San Francisco	Co-Investigator
Dr. Arianna Zanolini, PhD CIDRZ	Co-Investigator
Monika Roy MSC Clinical Research, University of California , San Francisco	Co-Investigator
Nancy Czaicki, MSPH, University of California Berkeley	Analyst

1.0 Background and Introduction

1.1 Rationale for study

1.1.1 Emerging Challenges in Global HIV Programs

The global HIV response has grown far beyond early expectations but continues to face formidable and evolving challenges at the policy, organizational and individual health worker and patient levels [1-3]. As HIV programs transition to country ownership to ensure sustainability, gaps in capacity and resource commitments have emerged. Health delivery systems involved in the response have been strengthened by infusions of resources, but are increasingly stretched by the sheer volume of individuals seeking chronic care, especially in countries with the highest burdens of disease, such as Zambia - where there are an estimated 1.1 million persons living with HIV, an HIV prevalence of 12.7% and approximately 30,000 AIDS deaths annually. Greater numbers of HIV-infected patients are also entering a phase of long-term follow-up. A new generation of innovative interventions is needed to overcome these multifaceted barriers to optimization of the engagement of HIV infected patients with the public health systems that have emerged to serve them.

1.1.2 The Need to Understand Patient Outcomes

As the HIV/AIDS response has evolved from an emergency scale-up to building sustainable systems of delivery, the need to understand patient outcomes in care has become increasingly critical to guide decision-making [4]. A better understanding of patient outcomes will allow for better assessment of public health systems and programs efficiencies. "BetterInfo" seeks to better understand lost to follow up and the drivers thereof; and to use this information to strengthen programs and identify potential interventions for improving patient retention. Systems to capture outcomes, however, have lagged far behind systems to deliver services. After enrolment in HIV care, many settings have observed a high fraction of loss to follow up in programs in sub-Saharan Africa, including in Zambia [5-10]. Although often portrayed as a homogenous group, those classified as lost to follow up (LTFU) actually comprise at least three distinct states: death, "silent" transfers to other health facilities, and full disengagement from healthcare – each state requires different public health responses. The large numbers of LTFU patients means that ascertainment of all outcomes would require tremendous resources at a population level. Yet, such information is absolutely critical to understanding program performance. To obtain meaningful estimates without tremendous resources, individual HIV clinic sites in East Africa have applied a sampling-based approach. This approach has revealed that retention may be underestimated by up to 50% [11-14] and mortality by as much as 80% [15]. Furthermore, the sampling-based approach has also shown that analyses that do not account for outcomes among the lost can result in both spurious and missed associations [16, 17], which may potentially mislead programmatic responses. In short, in the present environment, we lack a systematic understanding of patient outcomes which is hampering data driven programmatic improvement.

1.1.3 A Sampling-Based Epidemiologic Strategy

In order to more accurately estimate patient outcomes where LTFU is high, this proposal uses a sampling based approach in which we identify a numerically small **but randomly selected** sample of lost patients and **intensively seek them to ascertain their true outcomes** (e.g., death, "silent transfer" to other facilities, or disengagement from care). Outcomes in a random sample are by definition an unbiased estimate of outcomes in the underlying population [18]. The outcomes ascertained among the lost are then incorporated, using a probability weight, into overall program results to yield corrected estimates of mortality and retention in the entire patient population. We will also apply in depth interviews and surveys among traced patients identified

as LTFU to understand what configuration of factors most influences decision to transfer or disengage from care.

1.1.4 Better Information to Support Policy and Implementation Objectives

We are aware that novel approaches in health too often fail to influence routine practice. To ensure that this initiative is feasible, potentially adoptable and can be disseminated easily within Zambia, we will not only focus on tracing lost patients and their outcomes, but we will also carefully assess and report on the implementation process of this approach [19, 20]. We will continue to plan and execute this project in close engagement with the Zambia Ministry of Health, thereby incorporating views and priorities of the Ministry of Community Development, Mother and Child Health and Provincial and District-level health offices, thereby accounting for health service delivery and implementation priorities within the national health system. At the end of the study we seek to not only show the feasibility of carrying out a novel epidemiologic approach, but to also be poised to use it to its maximal utility.

1.2 Significance

1.2.1 Understanding outcomes in HIV care and treatment is crucial

Effective and efficient delivery of quality HIV care and antiretroviral therapy (ART) is essential to achieving global targets for improvements in health outcomes, including reductions in HIV incidence and mortality. HIV programs in Africa, however, face particular challenges in assessing critical patient outcomes such as mortality and retention in care. Many programs across the continent have reported rates of loss to follow-up (LTFU) as high as 25-40% [11-13, 21]. Patients lost to a particular program or site may be dead, have sought health care elsewhere, or disengaged from the system entirely. Without understanding the frequency of each of these outcomes – and how these vary across patient populations and over time – routine estimates of outcomes may be severely biased. Previous work has suggested that because the fraction of patients among the lost who have died is high, failure to account for outcomes in the lost means that mortality is possibly underestimated [15]. The lost also include large numbers of patients who must travel to maintain mobile livelihoods, and therefore not accounting for their outcomes leads to underestimates of retention in care [22]. In this manner, LTFU undermines overall assessments of, and strategies to improve, the effectiveness of programs [11-13].

1.2.2 Knowledge of outcomes drives data-driven public health practice and policy

Decision making in health must incorporate the effects of policies and programs on patient health outcomes to be efficient and effective. Over a generation ago a simple but now widely used model for understanding the nature of health service delivery was put forth. It conceives of health service delivery as comprised of three components: structures (material and human investments in health and the policies and guidelines that guide them), processes (the activities, decisions and interactions that take place at all levels of the health system including actual interactions between patients and delivery systems) and outcomes (health status of the patients such as survival, disability, and quality of life as well as population level outcomes including financial protection and equity.) [23]. The President's Emergency Plan for AIDS Relief, the Global Fund, and national governments from around the world have made tremendous investments in the structures to deliver HIV care in resource-limited settings. Public health scientists now have the imperative and the opportunity to study how the huge diversity of program processes actually influences patient outcomes, and how to optimize those delivery strategies within the specific and distinctive social, cultural, economic, and program contexts in Africa. Measurement of outcomes is a crucial step in this process.

1.2.3 The future of the global response to HIV is a transition to country ownership

In the global response to HIV/AIDS, the importance of outcome measurement is amplified by the massive transition in management of programs from international donors to country ownership. Without methods to accurately assess outcomes, national governments and donors will have limited ability to monitor and react to problems, including potential slippages in quality. Furthermore, without accurate outcomes, targeted work to identify the determinants of both mortality and loss from care, many of which are attributable to modifiable aspects of program implementation, can be biased. The importance of outcome measurement was crystalized in a 2013 Government Accountability Office report to Congress titled, “PEPFAR: Shift toward Partner-Country Treatment Programs Will Require Better Information on Results.” The report recommended PEPFAR “establish a set of minimum standards for data generated by partner countries’ M&E systems.” (http://www.gao.gov/products/GAO-13-460). This study therefore seeks to enhance assessments of outcomes and their determinants, thereby facilitating larger policy objectives in the global response to HIV. Assessing outcomes among patients LTFU provides the ability to evaluate the effectiveness of treatment in the entire population supported by the CIDRZ program that contributes almost 40% of all patients in the national ART program. Critically, the use of mixed methods approaches will enable us to understand the reasons for these lapses in effectiveness, and apply novel perspectives from implementation sciences to document the uptake of this process, positioning these results to be optimally used in wide public health practice.

1.3 Aim

This study seeks to use a novel epidemiologic approach- based on sampling outcomes among lost to follow-up patients in HIV programs- to obtain meaningful estimates of engagement in care and mortality in a real world setting where loss to follow-up is high. Furthermore, this study will describe how service-related, socio-cultural and economic factors contribute to these outcomes. The study will also enable assessments of program effectiveness and comparative effectiveness across settings and allow us to better understand the implementation process and outcomes of this approach within the larger public health monitoring and evaluation systems. We believe this will strengthen program efficiencies and support country ownership.

To ensure that the study design and process is successful and relevant, we will first conduct a smaller pilot study. Once completed, these data will be rapidly reviewed and analysed and used to adapt or hone the larger study before implementation if necessary. The pilot study will be conducted in 8 of the total 30 clinics and will include patient tracing of approximately 160 patients and measurements of drivers of patients outcomes. Following this, phase two will include all 30 clinics and include approximately 5,000 patients and their outcomes.

<u>The study timeline is as follows (also outlined in Section 5.0):</u>

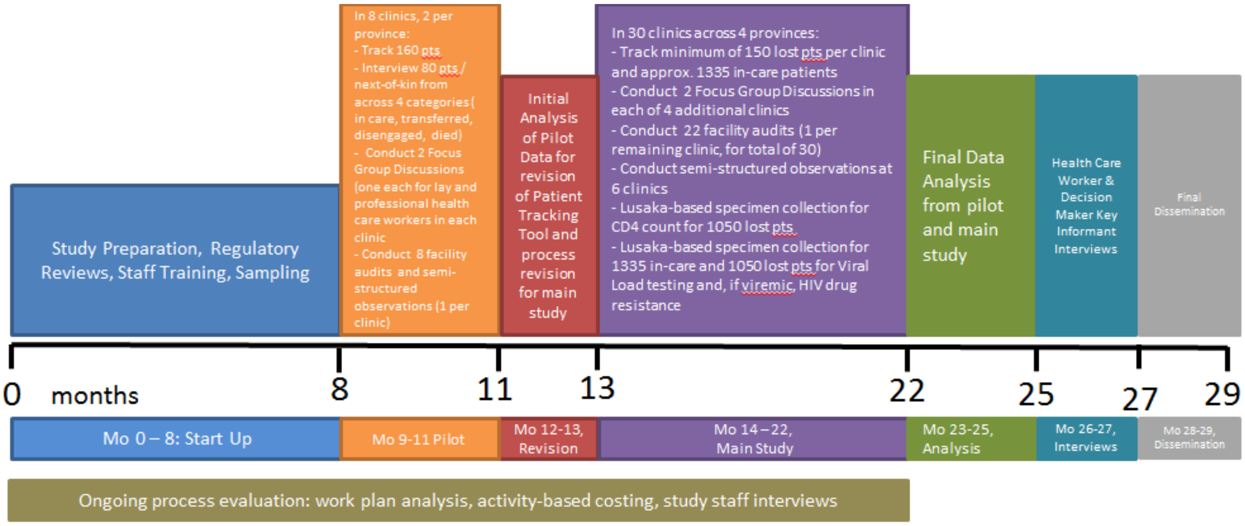

1.4 Specific Objectives

a. We will obtain crucial measures of programme outcomes in Zambia that are representative at a regional (provincial) level.

b. We will examine critical factors that influence programme success.

c. We will systematically gather information about the processes needed and the challenges faced in collecting such information in the context of existing national programs.

Our findings will help to inform broader regional and global initiatives to standardize monitoring and evaluation, and provide regional data in Zambia to further optimize health services for HIV care and treatment. We will share findings throughout the study process and through various fora to support application-oriented dissemination of the revised estimates, patient outcome drivers and process evaluation outcomes.

1.4.1 Objective 1: Application of a sampling based approach to incorporate outcomes among patients lost to follow-up into overall assessments of program effectiveness

We will assess overall effectiveness of the HIV care and treatment cascade in four provinces, Southern, Eastern, Western and Lusaka, using a sampling-based approach to estimate mortality, and retention among all patients who have enrolled in care. The application of this approach across these four provinces will enable us to provide regionally representative estimates of the above-stated outcomes. We will also add laboratory-based assessments in Lusaka Province using point-of-care CD4 count for pre-ART patients (to determine subsequent eligibility) and collection of a dried blood spot for ART patients for HIV RNA quantification and HIV drug resistance (HIVDR). We shall measure patient CD4 counts. However these results will not be used for patient clinical care, therefore we shall ask patients to get a repeat when they return to their clinic. These findings will greatly enrich the current metrics of health status outcomes and assessments of program effectiveness. We will use standard instruments for satisfaction, respect, activation [24] and empathy in lost patients, comparing them to a subsample of "in care" patients to identify differences between the groups and highlight potential areas for intervention. We will ask questions about patient social environment and demographic factors. We will also solicit patient preferences about the health care environment by presenting a series of attributes and record their responses in a discreet choice survey.

1.4.2 Objective 2: Determine drivers of engagement and dis-engagement in care through study of patient perceptions, provider experiences, and facility-level health system factors

We will use a mixed methods approach to assess patient and provider experiences and perceptions related to the causes of disengagement from HIV care. In-depth interviews and focus group discussions will be carried out with a sample of providers, patients in care, patients disengaged from care, and in cases where patients have died, the families of the deceased. These individualized data will be triangulated with structured and semi-structured observations of health facility operations. This mixed-methods approach recognizes that poor patient outcomes result from a complex interplay of barriers, which likely vary by individual and also across socio-cultural and operational settings. Data about the reasons for disengagement will be collected among those patients who are successfully tracked in the pilot phase of Objective 1. Focus group discussions with health care workers, health facility audits and semi-structured observations in facilities will also take place during the pilot phase of Objective 1.

1.4.3 Objective 3: Identify epidemiologic predictors of outcomes

We will use multi-level epidemiologic methods to identify structural, clinic-level and patient-based factors associated with sample-revised measures of mortality, retention and HIV RNA suppression [25].[25]. We have previously shown how data obtained from this sampling-based methodology can drastically alter predictors of negative patient outcomes. In this proposal, we extend our previous work by applying our methodology to a probability sample of 30 sites taken from 76 sites across four provinces. The scope of this analysis allows multi-level analysis in which we assess the association between outcomes and site level factors, using data collected under Objectives 1 and 2. These analyses will inform the assessments of the basis of effectiveness. We will also use instruments for satisfaction, respect, activation [24] and empathy in lost patients, comparing them to a subsample of "in care" patients to identify differences between the groups and highlight potential areas for intervention.

1.4.4 Objective 4: Understand the process of implementing a sampling-based approach to address lost to follow-up

As research and evaluation becomes increasingly "pragmatic," [26-28] investigators now seek not only to provide results, but also to document the implementation process, so that any results of value can be more easily replicated. We will therefore carefully document and evaluate the "implementation outcomes" [19, 20] of this sampling-based approach to understand key factors that influence future utilization in wider programmatic settings. Important outcomes of interest will include perceptions of appropriateness, acceptability, fidelity, adoptability, cost, and others [19]. These assessments will aid in understanding the eventual application and disseminate-ability of these methods elsewhere [29]. These implementation outcomes will be assessed through process evaluation measures and key informant interviews.

1.5 Ethical Issues

This protocol, the informed consent documents, and any subsequent modifications will be reviewed and approved by the UNZA Biomedical Research Ethics Committee (BREC) and the necessary IRB's responsible for oversight of the study. Further information on participant risks, benefits and other considerations for human participants are addressed in Section 3.5 of the protocol 'Considerations for Human Participants'.

Patient tracing will follow the tracing procedures that are currently implemented in standard clinical practice. This includes finding the patients, identifying their outcomes, and encouraging them to return to care. When relevant, this also means speaking with family members of the deceased to determine the patient outcome. Tracing is conducted with the utmost care and concern for patient confidentiality. This will include practices such as training of patient tracers, stating only health-related purposes or other socially acceptable, non-identifiable reasons for any visit, and consulting health care workers and key stakeholders at the study-affiliated clinics to identify other important tracing practices to maintain

confidentiality. In addition, tracers will not wear any identifiable clothing or badge when making household visits. When possible, we will gender-match staff members doing the tracing and patients who are being traced or send tracers in pairs.

Prior to informed consent, the study tracing teams will only conduct activities as outlined in the current tracing procedures. Any further activities will occur only if informed, voluntary consent is granted by the potential participant. When patients who are traced are found, they will be encouraged to re-engage in care if they are disengaged, supporting the programmatic objectives of the ART programmes operating in the clinics that comprise the study sampling frame.

2.0 Literature Review

Given that, in many reviews on retention in care, the fraction of patients LTFU ranges from 10% to 50% [11, 13], a critical gap in the literature exists. There are few studies that document the outcomes of lost patients. Those that do document outcomes (2.2 below) show that outcomes vary across settings. There are no studies that estimate the outcomes of lost patients in Zambia. Survey epidemiology demonstrates that unbiased estimates can be obtained from a random sample of the population in question (2.4 below). This study seeks to use well-established sampling methodologies to understand what happens to Zambian patients who are lost from HIV care, allowing for better health decision making and, ultimately, improved patient outcomes (2.5 below).

2.1 Outcomes of HIV-infected patients in Africa after engaging care is not well documented

Despite over 60 billion dollars invested in responding to the global HIV epidemic and over 10 billion dollars spent on treatment, the outcomes of treatment – the results of the response—are not well understood. This is largely because the scale up occurred rapidly in settings without either comprehensive surveillance systems or integrated medical record keeping infrastructure that could provide meaningful surveillance. As a result, outcomes are generally only available at the level of individual clinic sites, and often at this level, there is a large proportion of patients that have unknown outcomes due to loss to follow-up. In many reviews on retention in care, the fraction of patients LTFU ranges from 10% to 50% [11, 13]. The consequence of high loss to follow-up is that many deaths are not documented. Second, retention in care across the system is also poorly ascertained. Without accurate figures relating to retention and survival, the overall effectiveness of public health programs is uncertain.

2.2 Current literature suggests that the fate of lost to follow-up patients is heterogeneous and varies from setting to setting

Relatively few studies to date have sought to identify outcomes among lost patients, but in those that have, between 10% and 60% of patients have died [30-32]. A number of studies also suggest that 30% to 90% of patients who are not dead are in care elsewhere [32]. This wide variability of these figures suggests that outcomes among the lost differ markedly from setting to setting, therefore precluding meaningful summaries at the national or regional level.

2.3 Studies based on modelling outcomes among lost to follow-up patients have limitations

Several strategies have been proposed to correct estimates for the “unknown” outcomes in the lost to follow-up, but each of these has limitations. A “nomogram” has been proposed which is based on a correction factor which assigns a death rate that is related to the overall loss to follow-rate [33]. This approach, however, assumes that a single correction factor holds, and is also unable to adjust for analytic estimates. Second, inverse probability of censoring weights (IPCW) has emerged as a method to address unknown outcomes in the setting of longitudinal cohorts [34]. These techniques are powerful, but must

rely on assumptions that differ across settings. We have suggested that in African HIV treatment programs, the outcomes directly influence outcome ascertainment and therefore violate the "censoring at random" assumption that underlie IPCW estimation [16].

2.4 A well-established literature in survey epidemiology informs an efficient approach to assessing outcomes in HIV programs

Alternatives to estimation-based methods to account for missing information include design-based survey approaches. In short, survey epidemiology, the science behind censuses and polls, seeks to identify sampling frames and then draw observations from those frames with a known probability. Knowing the probability that a particular individual is sampled, allows investigators to understand how many others in the population can be represented. By drawing a random sample, the measured characteristics are, by definition, unbiased [35]. Stratification of the sample allows greater statistical efficiency as well as guaranteeing reasonably precise estimates within categories of interest [36]. Cluster sampling allows the ability to reduce costs and simplify the logistics of survey sampling. Finally, post-estimation weights can make further use of data from sites that do not have re-sampled outcomes, but that do have rich baseline covariates on patients. We apply these principles in our approach to first sample sites and then individuals lost to follow-up within those sites to make empiric measurements of outcomes in the Zambia context.

2.5 A better understanding of health outcomes leads to a better understanding of effectiveness of health services

Biomedical science increasingly recognizes that the real impact of health services is based on understanding patient outcomes in real world settings [37]. Research in real world settings, however, has lagged behind traditional population sciences because in many settings (such as Africa as well as the United States) no single comprehensive database of health services and outcomes is available. Therefore, directed evaluation to sample and examine "real world" populations is needed. Efforts to do so have found tremendous unexplained heterogeneity in outcomes across small geographical areas [38]. A similar evaluation of outcomes, and comparative differences, is needed to guide our understanding of the success of services.

3.0 Methodology

We will evaluate approximately 5,000 patients lost to follow up and approximately 1,335 patients currently engaged in care, to be conducted in Lusaka, Southern, Eastern, and Western provinces of Zambia. We will employ a sampling-based survey methodology with all participants and collect blood specimens from a sub-sample of patients in Lusaka province. The primary study outcome of interest is revised estimates of lost patient HIV care status: disengaged from care, engaged in care at a different site, or dead. In order to better understand patient preferences regarding factors related to re-engagement in care, we will ask patients to give us their preferences when presented with different potential attributes of the health delivery environment. To understand the drivers of the outcomes, we will also conduct focus group discussions with health care workers and in-depth interviews with patients and families. To better understand clinic-related influences on outcomes we will conduct health facility audits and patient-provider observations. Finally, we will conduct a process evaluation of the study in order to define and evaluate the approach to inform potential future implementation.

The study is explained according to four objectives. Objective 1 describes the sampling-based approach to trace patients and determine their outcomes. Objective 2 describes the qualitative work that will inform outcome drivers and examine the interactions between different influences on patient outcomes. Objective

3 describes the analyses that will determine revised outcome estimates, identify outcome predictors and other meaningful associations. Objective 4 describes the implementation science process evaluation.

3.1 Objective 1: Application of a sampling based approach to incorporate outcomes among patients lost to follow-up into overall assessments of program effectiveness'

Research Methods

Objective 1 is to evaluate the HIV care and treatment cascade in four provinces using a sampling-based approach to estimate mortality, retention and – in one province – HIV RNA suppression. We will utilise a sampling-based epidemiologic strategy to generate revised regional (provincial) estimates of patient outcomes in Zambia. In HIV-prevalent settings where the absolute numbers of patients is very large, intensively tracing all LTFU patients to comprehensively account for outcomes is not a viable strategy for program evaluation. However, identifying and tracing a numerically small – but randomly selected sample – of lost patients, and then incorporating these outcomes into the underlying patient population, represents a feasible strategy to recover broadly valid estimates.

Objective 1 will trace approximately 5,000 patients who are lost from HIV care across 30 health facilities selected from a stratified random across 4 provinces. Study staff will trace patients using standard clinical practice including learning the patient outcome (disengaged from care, in-care at a different facility or dead). The patients, or next-of-kin of deceased, will then be invited to voluntarily enrol in the study. They will be asked questions to help understand the predicators of the various outcomes. This will yield provincially-valid revised outcome estimates. In a sub-sample of participants, including a sample of in-care patients, study procedures will also include biological measurements of CD4 and viral load results, with HIV drug resistance if viremic. All Objective 1 elements are explained in greater detail and summarised in the table below.

<u>Summary Table of Objective 1 Research Activities</u>

Research Activity	Number (Approx.)	Sampling Criteria	Data Collection
Trace Lost Patients	5,000	• Visit for HIV care at one of 30 clinics in the study sample in 2 years prior to sampling<br>• Enrolled in HIV care at age 18 or older at their last visit date<br>• Next-of-kin to a patient who has passed away, who was in the 'current clinic population' and traced to determine patient outcome<br>• Willing/able to give informed consent<br>• Greater than 90 days late for last visit or 180 days since any visit	• Short survey including vital status and care outcome with patient or, if patient deceased, next-of-kin<br>• Socio demographic and laboratory measures from patient records<br>• DCS<br>• Administer short care experience 'empathy', 'satisfaction', 'activation', and / or 'respect', stigma, violence, alcohol use, demographic questionnaires
Trace In-Care Patients	1,335	• Visit for HIV care at one of 30 clinics in the study sample in 2 years prior to sampling<br>• Enrolled in HIV care at age 18 or older at their last visit date<br>• Willing/able to give informed consent<br>• In-care patient: visit for HIV care < 90 days	• Short survey including vital status and care outcome<br>• Socio demographic and laboratory measures from patient records<br>• DCS<br>• Administer short care experience 'empathy', 'satisfaction', 'activation', and / or 'respect',

			stigma, violence, alcohol use, demographic questionnaires
Measure patient care experience among those on ART	2,385	• 1,050 lost, on ART patients in Lusaka (included in 5,000 above)<br>• 1,335 in-care, on ART patients in Lusaka (included in 1,335 above)	• Collect blood for viral load testing and HIV drug resistance testing if viremic<br>• Administer short care experience 'empathy', 'satisfaction', 'activation', and/or 'respect', stigma, violence, alcohol use, demographic questionnaires
Measure patient care experience among those pre-ART	1,050	• Lost, pre-ART patients in Lusaka (included in 5,000 above)	• Collect blood for CD4 testing<br>• Administer short care experience 'empathy', 'satisfaction', 'activation', and/or 'respect', stigma, violence, alcohol use, demographic questionnaires

3.1.1 Design (Objective 1)

We will first sample approximately 30 clinic sites from the approximately 78 clinics across the 4 provinces. Within clinic sites, we will identify all lost and, in some facilities, in-care patients. We will intensively seek outcomes information about a random sample using clinic-based records and communication with the patient. The patient tracing activities will be conducted in accordance with the standard operating procedures pertaining to current clinical practice. These clinical practice standard operating procedures include tracing the patient, determining the patient's vital status (alive or passed away), learning if the patient has stopped care or transferred to another care facility, and encouraging the patient to return to care. After these procedures are completed, the patients (or, in appropriate cases, the family members of deceased patients) will engage in an Informed Consent process to indicate their voluntary willingness to participate in the study. Only patients and family members voluntary willing to consent to participate in the study will continue with further procedures. Those patients and family members who elect not to sign the informed consent documents will be excluded from the study and no further information will be collected from them.

We will then estimate engagement in care and mortality at each step of the cascade. Most estimates will take the form of cumulative incidence of mortality and engagement between enrolment and staging, eligibility and ART initiation and after ART initiation. In addition, in Lusaka Province, we will make additional biological measurements to better describe the HIV treatment cascade.

3.1.2 Patients (Objective 1)

For Objective 1, the target population is the current HIV-infected adult (18 years or older) population who has made a visit to one of the approximately 30 randomly selected GRZ, CIDRZ-supported clinics in the two years before implementation of the study. This sample includes both new patients as well as any patient who has been in the clinic before the calendar period of interest (the previous two years). The electronic medical record has information on all patients not lost to follow-up. We will use data on these patients, supplemented with outcomes among those lost to follow-up and traced, to obtain overall estimates in the CIDRZ-supported sites. The current clinic population enrolled in these GRZ facilities is approximately 469,720 individuals as of April 2014 of which an estimated 140,916 have been classified as lost to follow up (defined as being greater than 90 days late for their last scheduled visit). For logistical and financial reasons,

this population is far too large to be effectively traced in the community to comprehensively assess outcomes. Ensuring efficient assessment of this population motivates a multi-level, stratified sample of clinic sites and then patients lost to follow-up within each site for intensive tracing. In Lusaka Province, we will also target a population of actively in-care patients to better understand biological outcomes. Sampling of this population will enable us to assess individual patient-level outcomes (i.e. dead; alive but transferred; alive but disengaged from care) as well as produce revised estimates of the proportion of each of these outcomes for: i) each of the 30 sites and ii) at the Provincial level.

In-care patients will be identified as follows for sampling: HIV-infected adult (18 years or older) patients who have made a visit to one of the approximately 30 randomly selected GRZ, CIDRZ-affiliated clinics in the two years before implementation of the study and who are not lost to follow-up, died nor transferred out. We will randomly or systematically select patients from this pool of in-care patients. We will seek to match in-care patients with lost patients on observation time, thus allowing calculation of rate ratios for becoming disengaged from care due to time-invariant exposures.

Inclusion Criteria for Patients and Next-of –Kin who will be traced in the pilot or main study:

1. Had visit for HIV care at clinic in the study sample in the two years prior to patient-level sampling (part of 'current clinic population')
2. Enrolled in HIV care at age 18 years or older at their last visit date.
3. Next-of-kin to a patient who has passed away, who was in the 'current clinic population' and traced to determine patient outcome
4. Willing and able to give informed consent in English, Nyanja, Tonga, Bemba or Lozi
5. If clinic where known to be last enrolled in care is in Lusaka, Southern, Western or Eastern Province: Greater than 90 days late for last scheduled clinic visit or 180 days since any visit to the clinic if no scheduled return date is known
6. If clinic where known to be last enrolled in care is in Lusaka Province only: Had clinic visit for HIV care within past 90 days
7. In-care patient who is in the current clinic population at one of the study sites

Exclusion Criteria for Patients and Next-of –Kin who will be traced in the pilot or main study:

1. Under the age of 18 years
2. If clinic where known to be last enrolled in care is outside of Lusaka Province: Known to be actively in-care (had a clinic visit in past 90 days)
3. Enrolled in care at clinic that is not included in study sample
4. In-care patient exclusion: Patient who has died, has transferred out, or is lost (90 days late for a clinic visit)

Additional Inclusion Criteria for Patients who will complete Viral Load Testing:

1. Part of current clinic population in Lusaka Province-based clinic included in study sampling
2. Last known status for in-care or lost patient is 'on ART'

Additional Inclusion Criteria for Patients who will receive HIV Drug Resistance Testing:

1. Viral load test found to be viremic

Additional Inclusion Criteria for Patients who will complete CD4 Testing:

1. Part of current clinic population in Lusaka Province-based clinic and identified as 'lost' (Greater than 90 days late for last scheduled clinic visit or 180 days since any visit to the clinic if no scheduled return date is known)
2. Last know status for lost patient is 'pre-ART' or not yet initiated on ARVs

3.1.3 Sampling (Objective 1)

Our sampling strategy is designed to generate data on individual outcomes as well as improved estimates on patient outcomes (i.e. dead; alive but transferred; alive but disengaged from care) at the: i) health centre level and ii) Provincial level. These estimates will provide a firm basis for overall inferences about outcomes in the larger Zambian HIV-infected clinic care population. The specific sampling methodology outlined below will be applied to the entire population of CIDRZ-supported health facilities at the time of sampling. While they are yet to be confirmed, the current number of health facilities and patients in the sampling frame utilised in the calculation are a close illustration of what the actual sampling frame will be. The sampling cascade is depicted in Figure 1:

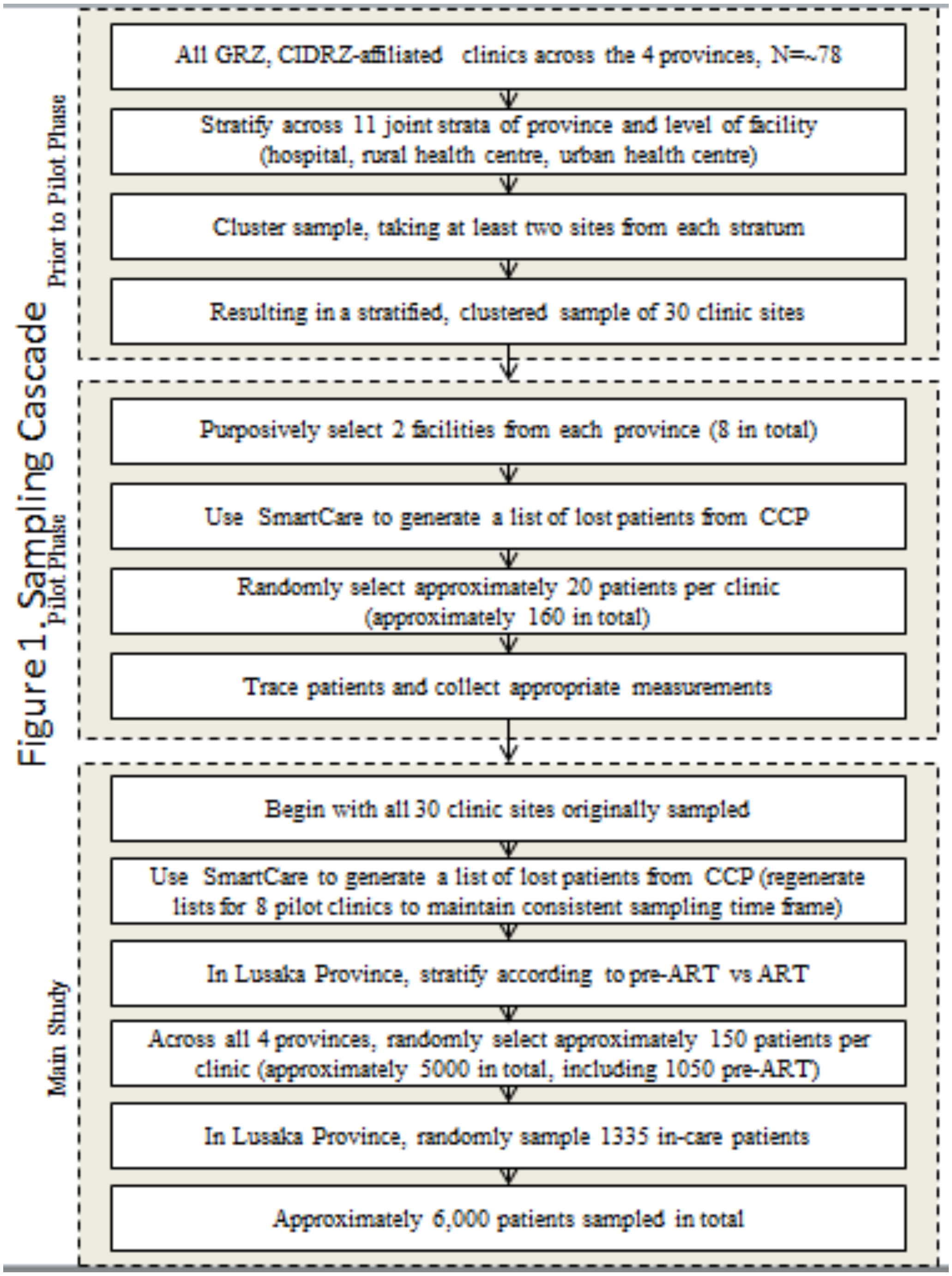

Figure 1. Sampling Cascade

Stratification

The study team has identified site level characteristics of interest to be provincial and three health facility levels (rural health centre, urban health centre and hospital). This means we will stratify the sampling across 12 joint strata of province and facility, with example sample strata outlined in Table 1. We consider a 10% width of the 95% confidence interval to yield actionable estimates.

Table 1. 11 proposed sampling strata

Province	Health facility level	Joint stratum number	Total facilities in stratum
1	1	1	6
1	2	2	28
1	3	3	2
2	1	4	3
2	2	5	5
2	3	6	3
3	1	7	5
3	2	8	7
3	3	9	6
4	1	10	5
4	2	0	0
4	3	11	6
TOTAL		76	

Health facility levels denote rural health centres, urban health centres and hospitals, respectively.

Clustering

Practically, the cost associated with sampling is largely determined by the number of sites. Therefore we will cluster-sample facility sites before selecting individuals with probability proportional to size. Two clusters per stratum are needed at a minimum to quantify the within and between cluster variability in the outcome of interest – the "rate of homogeneity" – needed for overall variance estimates. Based on past experience of recommended tracing time and feasibility, we will trace approximately 150 patients identified as lost per site. Fewer patients may be sampled in a site with a smaller lost patient population. . This sample size will allow for site-specific revised estimates of patient outcomes in addition to contributions of site-level data to regional (provincial) revised estimates.

Rate of homogeneity

Based on previous work in East Africa, we assume mortality of 30% among the lost and a rate of homogeneity of 0.15 in order to obtain relatively conservative projections of precision in each entire stratum. In stratum number 2 (urban health centres in Lusaka), we assume a higher rate of homogeneity of 0.25 because these units serve neighbourhoods with highly varied socioeconomic status, and therefore making it likely that the between clinic variability in outcomes (such as mortality) may be higher than between clinic site variability in other strata.

Anticipated site selection

Applying these considerations to the patient population in the CIDRZ-supported GRZ health facilities; we will select approximately 30 sites from 12 joint strata. We take 2 sites from each stratum then increase the number of sites in stratum two to 10 to reach a 95% confidence interval width of approximately 10% for a total hypothetical mortality of 10% (Table 2).

Table 2: Estimated weights for each individual sampled at both the facility level and then among those lost to follow up.

Joint stratum number	Total facilities in stratum	facilities sampled	Lost patients sampled	Lost patients found (assumed .85)	Probability of selection: 300 / Lost (or 1500 / [illegible]	Stratum weight: inverse of probability	Response rate adjustment: 1/RR [illegible]	Final weight: product of stratum wt and [illegible]
1	6	2	300	255	0.3175	3.1500	1.1765	3.7059
2	28	10	1500	1275	0.0178	56.2180	1.1765	66.1388
3	2	2	300	255	0.0767	13.0380	1.1765	15.3388
4	3	2	300	255	0.5291	1.8900	1.1765	2.2235
5	5	2	300	255	0.0376	26.6140	1.1765	31.3106
6	3	2	300	255	0.0489	20.4500	1.1765	24.0588
7	5	2	300	255	0.1603	6.2400	1.1765	7.3412
8	7	2	300	255	0.0396	25.2360	1.1765	29.6894
9	6	2	300	255	0.0254	39.3160	1.1765	46.2541
10	5	2	300	255	0.1871	5.3440	1.1765	6.2871
11	6	2	300	255	0.0211	47.3520	1.1765	55.7082

Anticipated lost patients traced

Within each of 30 sites we will seek a minimum of approximately 150 patients as described above. We therefore anticipate a minimum total of approximately 4,500 lost patients to be traced (150 patients per site x 30 sites). We will also consider over-sampling specific key populations, such patients who have previously been lost and returned to care, to ensure that inferences about that population can be made, likely approximately 10% of the sampled lost population or approximately 500 additional patients.

Sampling Weights

These considerations also provide projected weights for each observation in this sampling scheme - which vary substantially across strata (Table 2). We expect that approximately 42% of patients will come from Lusaka Province and 58% will come from the 3 other Provinces based on the geographic distribution of the population.

Sub-sample for biological measurements

In Lusaka Province, we will also sample patients who are actively in-care to obtain biological measurements of in-care HIV outcomes (See: ‘3.1.4.6 Obtain biological measures (Lusaka-only)’, below). Based on an estimate of the in-care HIV patient population in Lusaka, we will enrol approximately 1,335 Lusaka-based in-care patients who are on ART (an estimated 15% sample of in-care Lusaka-based patients then assuming 50% are on ART). We will also obtain biological measurements for a sub-sample of lost patients who are traced in Lusaka (CD4 for lost pre-ART and viral load / HIV drug-resistance testing for lost on ART).

3.1.4 Procedures (Objective 1)

Procedures for the sampling-based approach will include:

1. Enumerating the "current clinic population"

To define the current clinic population, we need to identify those patients who have been active within the last two years. This includes all adult patients, aged 18 years and older at the time of enrolment in HIV care who have enrolled during this time period or have accessed services within the last two years, regardless of date of enrolment. Operationally, the cohort definition will be defined by the last visit date at the time of evaluation falling within two years. We will filter SmartCare for patients who meet this characteristic and select for adults to yield the underlying "current clinic population" (CCP). The estimates of the outcomes of interest in this CCP will serve as the baseline estimate which will revise using the information learned during the study. We will draw data from the electronic medical record again at the end of the study to extend observation through the end of the study.

2. Identifying lost patients and selecting a random sample

Follow up of patients who have dropped out of care is not currently standardized across sites or regions. However, most sites have implemented systems for tracking patients and identifying those who require follow up. We will use the site supported methods of identifying patients for follow up whenever possible. This is likely to include both the use of SmartCare generated late lists and the use of manual, paper-based registers.

Lost patients will be identified from the CCP and defined as those who are greater than 90 days late for their last scheduled visit at the time of database examination or six months (180 days) late for patients with no return date recorded at their last visit. These criteria will be applied in a statistical software package, using SmartCare data or register data. In select facilities and during the pilot, this will also include identifying in-care patients. Staff at each site will receive a list of approximately 150 lost patients. Where SmartCare is used, the study team will review the paper charts of all patients lost to follow-up who are listed for tracking. The paper record may contain patient outcome information that is missing in the electronic database. This may be because visit data were not entered into SmartCare and therefore the patient appears to be lost. Also reports of patient deaths are often written by hand on the cover of a chart but never entered into the database. In both cases, if this is found to be the case, their actual status from the paper record will be updated in SmartCare.

If a patient is not 'truly lost' (has a known outcome or is still active in care) according to the review of the paper chart, that patient will generally be replaced by the next randomly sampled patient on the 'lost' list generated through SmartCare. This will continue so that all 150 patients who are chosen to be intensively traced are 'truly lost,' after review of all clinic data. The estimates of the outcomes of interest among the truly lost population will provide outcomes figures that will be used to revise initial estimates of clinic outcomes.

It is possible that the SmartCare system may also misclassify patients who are lost as 'in-care.' If this concern appears to be relevant in the pilot phase, we may conduct a chart review of 100% of the patients identified as being in the CCP at 2-3 clinics in order to estimate the amount of misclassification of this kind. We will do this during the pilot phase to help inform measures that could contribute to more accurate estimates during the main study period. While 2-3 clinics will not give us a representative estimate of the amount of misclassification overall, it will provide an indicator of the challenge. The

study is limited to this number of clinics due to the time and human resources required to complete this review.

3. Tracing selected patients

   Once selected for tracing, patients will be sought first via telephone if a phone number is available. Failing that, the patient will be sought in person using information available in the patient file or known by health facility workers, via study or public transportation, bicycle or on foot, as appropriate. Once found, trackers will encourage the patient to return to care, as is done in most CIDRZ-supported patient tracing practice in the ART programme. Trackers will obtain consent for study activities including blood finger sticks (in Lusaka Province) and completion of a semi-structured questionnaire that records patient outcome and information about drivers of their outcome. Data will be collected from patient next-of kin if the patient is dead. Traced patients or families of patients who have passed away who choose not to participate in the study (do not provide consent) may be replaced by another lost patient from the CCP for tracing.

4. Documenting reasons for non-return among lost patients and reasons for remaining in-care among in-care patients:

   When a patient is found, a semi-structured questionnaire (the Lost to Follow Up Questionnaire) will be used to elicit reasons for non-return following informed consent for study participation. Patients who are found to be accessing services at an alternative clinic will be asked about their reasons for switching clinics. Patients who are not in care will be asked about reasons for discontinuing care. Reasons will be recorded and coded into categories based on mappings of the Behavioural Model for Vulnerable Populations as well as other appropriate social epidemiological models. This semi-structured questionnaire will be adapted prior to use based on findings from the mixed-methods research conducted during the pilot phase, to ensure it is contextually and culturally appropriate and as complete as possible in this setting.

   All in-care patients traced in Lusaka will be asked questions from the In-Care Tracking semi-structured questionnaire to better understand what contributes to their successful maintenance of in-care status.

5. Documenting patient experience

   We will also extend previous work by introducing new individual-level measurements including empathy, satisfaction, activation and respect among patients using survey tools. These concepts are the focus of renewed interest in primary care [39], and previous work from this group and others suggest that they play an important role in engagement of HIV patients in care in Africa [40, 41]. To ensure the clarity and validity of these and other tools used in the study, we will conduct basic cognitive interviewing (discussion about the question structure and meaning) with groups of study staff members or volunteers as a part of study preparation activities. No identifying information will be collected from any cognitive interview participants. Some of the experience scales use a likert scale (rating how much the participant agrees or disagrees). To best facilitate participant response, the study will either use choice options (listing strongly agree, agree, disagree, etc.); an analog scale where the participant points on a line to how much she/he agrees or disagrees or a 2-step choice scale where they first either agree or disagree then discuss how strongly.

6. Obtaining biological measures (Lusaka only)

In Lusaka province, we will obtain additional measurements from patients who have provided written consent. We will obtain blood through a finger prick to get CD4 results for patients who are identified as lost but not yet eligible for treatment (pre-ART) and viral load results (using a Dried Blood Spot for HIV RNA levels) for patients already on ART but identified as lost (lost from ART list). We will conduct HIV drug resistance (HIVDR) testing among those who are viremic. Of note, unlike the other measurements (visits, vital status and CD4), the HIV RNA measurements will also be taken from the clinic population still in care.

When they are available, we will return patient results to the clinic from which the patient was originally identified. We will subsequently inform patients that their test results are ready and have been submitted to the clinic. We will recommend they return to discuss these results with their provider. This will support good clinical care and will also provide additional encouragement to those disengaged from care to re-engage.

3.1.5 Measurements (Objective 1)

1. Socio-demographic and recorded laboratory measures

   We will extract standard de-identified socio-demographic (e.g., sex, DOB), laboratory (e.g., CD4, haemoglobin) and clinical (e.g., visits, WHO stage, diagnoses, pregnancy) data for all patients in the CCP from the electronic medical record (e.g. Smartcare and the Laboratory Information Management System). For patients who consent to participate in the study, we will extract identified data to support analysis of predictors of the outcomes of interest.

2. Vital status of lost patients

   For patients who are sought and reported dead, we will record date of death, approximate if necessary, as well as the cause of death in six simple, mutually exclusive and exhaustive categories: accident, homicide, suicide, child-birth, illness, and other.

3. Current care status of lost patients:

   Patients successfully contacted will be asked whether they have seen any provider for HIV care during the period defined as LTFU from their primary facility. Patients who report not having seen a provider / accessed a health facility for 90 days or more for those on ART and more than 180 days for those not yet on ART will be defined as being 'not in care'. Those in care elsewhere will be defined as "silent transfers" and the first date of visit to a new clinic after loss to follow up at the old clinic will be ascertained to establish whether a gap in care occurred.

4. New laboratory results:

   We will measure HIV 1 RNA levels, CD4 counts, and resistance genotypes among a subset of participants studied in Lusaka Province. We will conduct CD4 testing for approximately 1,050 lost pre-ART patients in Lusaka and viral load testing on approximately 1,335 in-care and 1,050 lost ART patients in Lusaka. HIV resistance testing will be conducted for those patients found to be viremic, estimated at 30% of those receiving viral load tests. At the study interaction, patients will be told to visit the clinic after a period of time when results will be ready (for example ' return to the health facility after 2 weeks'). If a viral load lab result is critical or abnormal, they will also be notified by phone or in person to report to the health facility as soon as possible to see a provider. These results and all others will be entered into the electronic medical record as

well as the paper file and will be accessible by the provider at the patient's next clinical visit. The patient will receive the actual results in-person from a care provider at the health facility. As a research study, given that CD4 is standard of care, study CD4 results will not be used for clinical care. Patients will be advised to get a repeat test when they return to the health facility.

5. Discrete Choice Surveys

As part of Objective 2 above, we will conduct an additional module of questions about preferences for models of health care using a technique called Discrete Choice Surveys. Discrete Choice Surveys, sometimes called 'discrete choice experiments' (or DCE), present participants with two possible models of health care that differ from each other in one or more characteristics. For each pair of models, participants choose the one that they prefer. We will limit this additional model to Lusaka province for logistical reasons linked to training and time constraints. Participants will include both disengaged and silent transfers. The module will be asked to a random sample of approximately 500 participants (see power calculations below).

Discrete choice surveys are aimed at better understanding which and to what extent various attributes of the health care environment influence re-engagement in care for ART patients. Preferences for different characteristics of the health care environment can be hard to tease out with traditional methods, because they entail ranking, trade-offs and interactions. Discrete Choice Surveys methodology can help understanding which characteristics matter the most to patients and how do they rank by calling participants to make choices of health care models that involve trade-offs in different dimensions. The theory behind Discrete Choice Surveys is based on Economics, using the idea that people choose what is best for them. Critically, according to this theory, the characteristics of a product/choice (called "attributes") determine how much they value that product or care scenario.

We will use the 5 dimensions of quality (accessible/ safe/ patient centered/effective/efficient/ and equitable) as a guiding principle behind the dimensions. For example, one model will depict a high level of patient centerdness and access, but a low level of efficiency, and it will be contrasted to a model that has a low level of patient centerdness and access, but a high level of efficiency. We will call these dimensions "attributes" and we call the actual values "levels". Participants will be asked to choose which of the two hypothetical models would they prefer, and after repeating similar tasks, we will be able to infer their preferences over the different attributes and their ranking. Two models hypothetical scenarios are presented at a time.,Each participant will get a maximum of 9 questions where in each question, they will choose their preferred model.

Using preliminary results from the qualitative work described in Objective 2 and referencing literature on factors that influence loss and re-engagement in care, we have specified the attributes that the survey will present below. Each model of health care will be defined by a specific level for each attribute. For example, a model could be "5 Kms

distance; overall time spent at the facility for each appointment of around 2 hours; getting 3 months of medications ad no stock-outs; staff not welcoming and sometimes rude; facility open Mon-Fri only"

**Guiding principle**	**Attribute**	**Level 0 of attribute**	**Level 1 of attribute**	**Level 2 attribute**
Efficiency	Waiting time	1 hour	3 hours	5 hours
Access	Distance	Less than 5 Kms	10 Km	20 Km
Effective/Safety	Months of supply of ARV given at each visit	Get ARVs for 1 month	Get ARVs for 3 months	Get ARVs for 6 months
Patient centeredness	Staff attitude	The staff is nice.	The staff is rude	
Patient centeredness	Opening hours	The facility is open Monday to Friday mornings only	The facility is also open on Saturday	The facility is open on the atfernoons as well

The design proposed above has 5 attributes, 4 with 3 levels, and 2 with 2 levels. This makes it a *3*3*3*3*2* design, which has a total of 162 models of health care, which can be paired up in (162*161/2) ways. Since this is a number of combinations too large to ask to any one person, we will utilize a fractional factorial design, which implies that we will select only an optimal fraction of all possible paired combinations available. There are several methods to achieve an efficient method to select the fraction of combinations that will be included in the experiment. General principles for an efficient design are that all levels should be represented with the same frequency in the selected group of questions, that within a pair of options the combinations should be as little overlapping as possible, and that the levels presented for each attribute should be uncorrelated (orthogonality). We follow the Street (2007) method of maximizing the D-efficiency to select an efficient design. We select a fractional factorial design and use a foldover to generate the model of health care that we contrast to (modulo-addition 1 for binary choices, modulo addition up to 2 for attributes with 3 levels, and modulo addition up to 3 for the attribute with 4 levels).

The specific formulation of the questions and example of one of the combinations selected through the fractional factorial design are included in the appendix. We divide the fractional factorial design combinations in blocks, so that each participant is asked a maximum of approximately 8 questions.

Sample size:

Standard power calculation rules are not applicable for DCSs because the scale of the impact of each attribute on total utility is a purely ordinal concept rather than cardinal

(higher estimate for one attribute means a higher contribution of that attribute to the utility). However, the rule of thumb based on a precision –based approach is to provide a sample size at least of 400 participants. This has been calculated as a result of simulations plotting the effect of simulated sample size on precision of the estimate in different studies. An example from a published paper is represented below. In the figure, gains in precision flatten out after 500 participants. We therefore target approximately 500 participants based on data from: Constructing Experimental Designs for DCEs: Report of the ISPOR Conjoint Analysis Experimental Design Good Research Practices Task Force, *Value in Health* 16 (2013).

6. Process measurements

We will measure several known process characteristics of patient tracking exercises, which include comprehensiveness of locator information available to the tracker (e.g. presence of a phone number), time spent tracking, number of informants contacted, weather during tracking, demographic nature of patient residence (e.g. urban; semi-urban; rural), and tracker characteristics (e.g. age, gender, experience, etc.). These will be related to the outcome of tracking (e.g. patient found; close informant found who provided information; or patient/close informant not found). These measurements will be used to inform the process evaluation covered in more detail in Objective 4. When possible, we will match the tracker and the patient being sought by gender and age characteristics to encourage higher community acceptance of tracking. Trackers may also work in a male-female team to improve acceptability in some communities.

Outside of the process evaluation, each set of measurements is notionally linked to relevant analysis in the graphic depicted below:

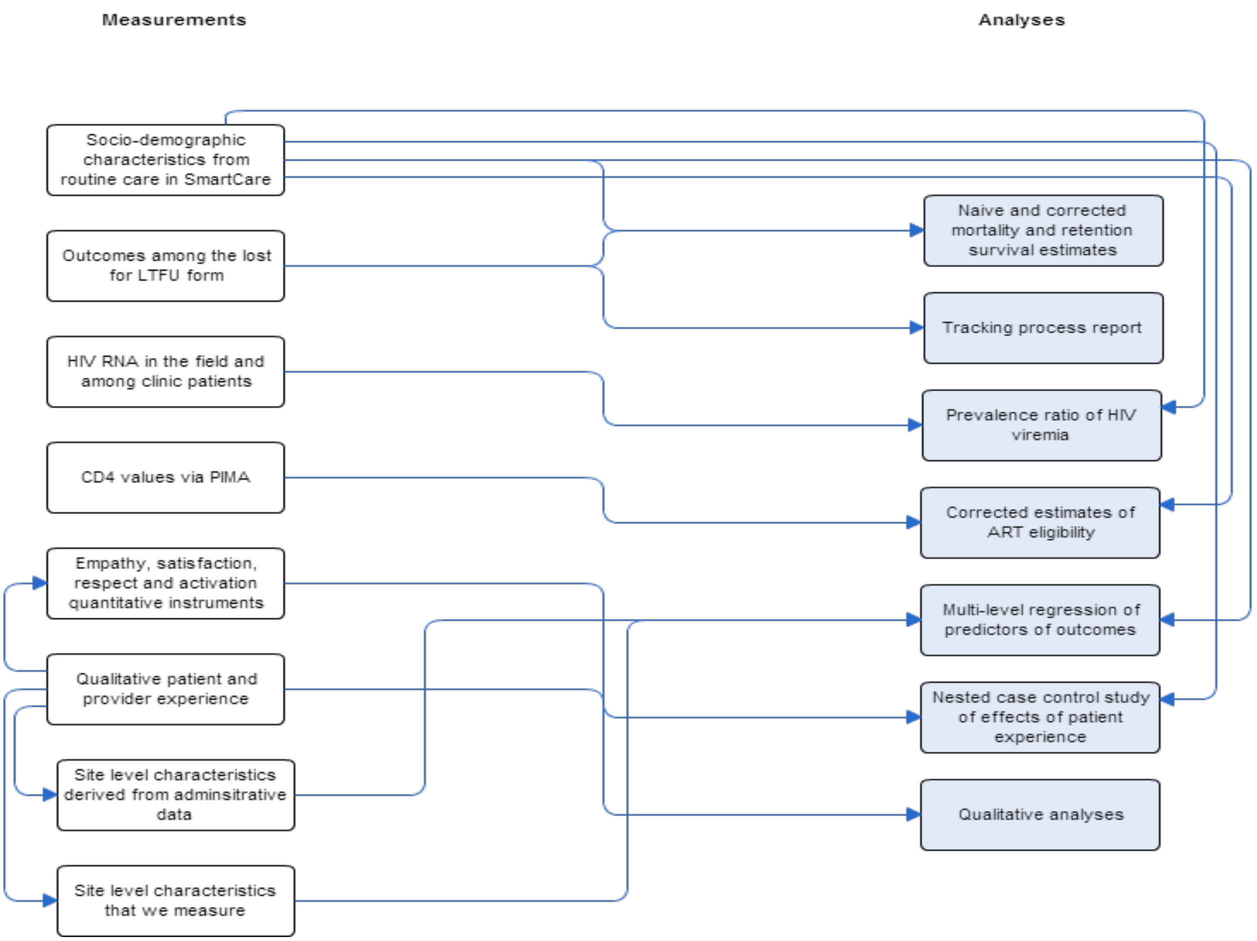

3.1.6 Analytic approach (Objective 1)

1. Determining the "naïve" or unweighted estimates of mortality and retention

Firstly, using only outcomes known to the clinics before our project sampling activities are implemented, we will estimate mortality and retention. These are the 3 'baselines' referenced above (i.e. disengaged from care, transferred, or dead). These analyses will follow standard survival analysis using Kaplan Meier, competing risk and Cox proportional hazards methods. These estimates will represent the "apparent" level of mortality and retention if outcomes among lost patients are not accounted for and will provide a point of comparison for revised sample-weighted estimates.

2. Analysing outcomes among lost patient who were traced

We will assess the proportions of each outcome among all lost patients traced in four mutually exclusive and exhaustive categories: died, alive but disengaged from care (patient alive and not seeing any HIV provider for 90 days since their last scheduled visit or 180 days from last visit if no visit was scheduled), alive but in care or "silent transfer" (patient alive and seeing another provider) or no further information obtained. We will look for factors associated with each of these states. Using Kaplan Meier techniques, we will estimate the cumulative incidence and hazard of mortality among the lost patients, beginning from the last visit at their original clinics [42]. For patients who were alive, we will estimate the time to connection at new clinic with Kaplan Meier plots.

3. Establishing revised (sample-weighted) estimates mortality and retention in the entire clinic population

    We will use Kaplan Meier plots or competing risk approach [43, 44] to estimate the cumulative incidence of mortality and retention - both overall and within strata -- at each step of the cascade (Table). Corrected estimates based on the outcomes of sampled patients will be equivalent to the method of Frangakis and Rubin [18]. The probability of inclusion in the analysis will depend first on the probability of site selection in each stratum. Therefore, the first stage of the weight for a given individual in a given stratum will be inverse to the probability within each joint stratum (defined by province and health level) of being selected.

    For example, if there are three clinics in a stratum, each with 1000 patients, and we randomly select two clinics (each with 1000), then the probability of being selected will be 2/3 and the weight each observation will get is 3/2 or 1.5. The probability of selection within each site will be used in a second stage of the overall weight each individual has in the analysis. Specifically, in a clinic, all patients who have known outcomes in the electronic records will be given a weight of 1. Those lost and sought will be weighted inversely proportional to the fraction with successful outcome ascertainment through tracking. The lost and not attempted to be traced will have a weight of zero. We will, in addition, explore the degree to which estimates change overall as we reduce the number re-sampled to understand how minimal and efficient the sampling can be while maintaining reasonably accurate and precise estimates. We will compare these corrected estimates to the "naïve" Kaplan-Meier estimate that simply uses available outcomes ascertained through routine clinic function. Confidence intervals will be obtained with bootstrapping.

4. Establishing revised estimate of distribution of HIV RNA (in previously initiated ART patients in Lusaka Province) and CD4 levels (in pre-ART patients in Lusaka Province).

    Among patients who present with high CD4 levels, only assessing eligibility among those who remain in care may substantially underestimate the cumulative incidence of ART eligibility over time. Estimates to date, using only data from patients who remain in care, fail to capture the number of patients who become newly eligible when they are not in care, thus missing an opportunity to initiate treatment in a timely way. Therefore we will study the CD4 counts obtained from the sub-sample of lost, pre-ART patients in Lusaka province.

    For patients on ART, prevailing estimates of HIV RNA failure are also cross-sectional and restricted to those patients who remain in care. HIV RNA levels in Lusaka will be measured in patients with varying levels of time since initiation of ART, and as such a single composite "prevalence" among all patients is not easily interpretable. We will stratify the findings of time on ART and therefore obtain the prevalence of HIV RNA viremia among those on treatment for 0-6 months, 7-12 months, 13-18 months, 19-24 months, and 24+ months.

    Of note these estimates will include the lost to follow up patients who are successfully traced, and will therefore allow for a better estimate of actual viral suppression rates among those who have started ART. We will stratify distributions based on follow-up status and also final outcome status. We will identify epidemiologic and site-level predictors of being viremic at each of those times.

3.1.7 Sample-size considerations

The overall sample size considerations in this proposal are based on a balance between our guiding principles of a minimal and efficient evaluation strategy which represents a proposed model for future dissemination and sufficient power to obtain revised estimates. With 11 joint strata, the numbers shown below, assumptions of 30% loss to follow up, roh of 0.15 for all stratum except 2 (where we assume .25), 150 traced per site, total and overall mortality of 10%, the table below (Table 3) shows the anticipated 95% confidence intervals accounting for both cluster sampling as well as resampling among the lost to follow up. The total width of the 95% confidence intervals in each stratum varies by joint stratum.

Table 3: Estimated 95% confidence interval width for total mortality of 10% in each stratum. We assume minimum of two clusters per stratum, 30% loss to follow up at each site, a minimum of 150 traced per site, and rate of homogeneity of 0.15 in all stratum except stratum 2 where it is assumed to 0.25becuase those clinics serve a more widely varied settings between sites.

**Stratum number**	**total number in stratum**	**Fraction of total patients in stratum**	**number of clinics in stratum**	**number of clinics sampled**	**average number of patients per clinic**	**deaths assuming 10% mortality**	**assumed " rate of homogeneity"**	**design effect (penalty due to cluster sampling)**	**total lost assuming 30% loss**	**total sought in stratum**	**fraction of lost sought**	**width of 95% CI for adjusted for resampling lost**	**width of 95% CI with accounting for cluster sampling**
1	3150	0.01	6	2	525	105	0.15	79	315	300	0.95	0.04	3.01
2	281090	0.60	28	10	10039	10039	0.25	2510	30117	1500	0.05	<0.01	11.17
3	13038	0.03	2	2	6519	1304	0.15	978	3911	300	0.08	0.01	12.14
4	1890	0.00	3	2	630	126	0.15	95	378	300	0.79	0.04	3.44
5	26614	0.06	5	2	5323	1065	0.15	799	3194	300	0.09	0.01	10.97
6	20450	0.04	3	2	6817	1363	0.15	1023	4090	300	0.07	0.01	12.41
7	6240	0.01	5	2	1248	250	0.15	188	749	300	0.40	0.03	5.21
8	25236	0.05	7	2	3605	721	0.15	541	2163	300	0.14	0.02	9.03
9	39316	0.08	6	2	6553	1311	0.15	983	3932	300	0.08	0.01	12.17
10	5344	0.01	5	2	1069	214	0.15	161	641	300	0.47	0.03	4.77
11	47352	0.10	6	2	7892	1578	0.15	1184	4735	300	0.06	0.01	13.34

3.2 Objective 2: 'Determine drivers of engagement in care through study of patient perceptions, health care worker experiences, and facility-level factors'

Research Methods

An HIV patient's retention in care is the product of various physiological, psychological, service-related, social and economic factors that interact in various ways to influence individuals' choices and behaviour through time. While previous literature allows us to hypothesize reasons for loss to follow-up, these factors vary considerably across different cultural, geographic and health system contexts.

To add explanatory power to this study and to inform the adaptation of the Loss To Follow Up Questionnaire used in the main study, research activities in Objective 2, will be carried out during the pilot stage. These activities will seek to identify how different factors combine in the Zambian settings to

influence individuals' engagement or disengagement in care and will be carried out in the 8 pilot facilities.

Specifically, we seek to:

(1) Identify what factors have influenced HIV patients' engagement in care and treatment

(2) Examine how different factors and contexts interact to influence those engagement outcomes.

We plan to answer these questions in relation to each of four categories: patients alive and in care at the original facility; patients alive and in care but transferred to another facility; patients alive but no longer receiving any care; and patients known to have died.

This will be done through in-depth interviews with patients and next-of-kin of deceased patients and focus group discussions with health care workers.

Objective 2 also includes health facility audits and observations of patient-provider interactions to further identify and understand factors that influence patient outcomes and how those factors interact.

3.2.1 Design (Objective 2)

To accomplish this objective, we will use a set of mixed methods, applied to the 8 clinics included in the pilot stage of this study. Mixed methods and qualitative research have explicit strengths for investigating the complex behaviours of, and relationships among, actors and organizational units [45, 46]. To support the generation of meaningful explanations for patient outcomes in each of these categories, as well as to inform and shape the Loss To Follow Up Questionnaire used in the larger traced patient sample under Objective 1, four core and inter-related research activities will be carried out: i) In depth interviews with a quasi-random selection of patients; ii) focus group discussions with a representative sample of professional and lay health care providers; iii) health centre infrastructure and equipment audits and iv) direct observations of health centre operations and patient-provider interactions. Table 4 below lists the number of each to be carried out.

Table 4: Summary of Objective 2 Research Activities

Research Activity	Number	Sampling Criteria	Exclusion Criteria	Data Collection
In-depth Interviews	Approx. 80	• 8 pilot clinics (1 rural / 1 urban per province)<br>• 1 M + 1 F in each of 4 categories* = 10 / clinic	• Individuals less than 18yrs;<br>• Inability / unwilling to provide consent<br>• Inability to participate in language other than English/Nyanja/Bemba/Lozi	Interview guide + recording & notes
Focus Group Discussions	Approx. 24	• 1 professional staff FGD + 1 lay staff FGD per clinic<br>• 8 pilot clinics (1 rural / 1 urban per province)<br>• + 4 purposively selected clinics during main study	• Individuals less than 18yrs;<br>• Inability / unwilling to provide consent<br>• Inability to participate in language other than English/Nyanja/Bemba/Lozi	Discussion guide + recording & notes

Facility Audit	Approx. 30	• All 30 randomly sampled facilities	N/A	Adapted facility audit tool
Direct Observations	Approx. 14	• 8 pilot clinics (1 rural / 1 urban per province) + 6 purposively selected clinics to ensure representation in all study strata<br>• Observations in 'public' zones of the clinic and OPD and ART screening rooms	• N/A for public zone<br>• Health staff not able / willing to provide informed consent for screening room observations	Hand-written research memos guided by observation themes

* **Patient categories:** i) alive, in care at original facility; ii) alive, in care transferred to another facility; iii) alive, disengaged from care; iv) dead – interviews with family member.

3.2.2 Interviews (Objective 2)

The study population will represent each of the four possible outcomes for patients (in care at original site, in care at another site, no longer in care, or died). Individuals who are still alive will be interviewed by trained team members. In our health facility focus groups, we will recruit professional and lay health care workers from a random sample of sites included in Objective 1.

1. Sampling for Patient Interviews

During the study pilot phase will carry out a series of in-depth interviews (IDIs) with a representative sample of: patients in care; patients disengaged from care; patients transferred, and in cases where patients have died, the families of the deceased. We plan to conduct approximately 20 IDIs with each group, for a total of approximately 80 interviews. We believe that this number will be sufficient to identify major themes and patterns and achieve saturation relating to the factors, and interactions between those factors, influencing patient (dis-) engagement from care.

Sampling for the qualitative interviews will be done in a phased manner. First, as part of Objective 1 (outlined above) 30 facilities will be randomly selected. From this random sample of 30 facilities, we will purposively select 2 facilities from each of the four provinces (8 in total). Selection of the 2 facilities per province will include one rural health centre, and either one urban health centre *or* level 1 hospital. We consider urban health centres and level 1 hospitals interchangeable for the purpose of the pilot and qualitative study, since they are often of similar size and operating capacity and are located in similar socio-economic and geographic environments. These selected facilities are the ones where the pilot will be carried out, including all related tracing, interview and other activities as listed in the timeline above.

For the interviews, from the overall random sample of clients identified (see Objective 1) in each of these 8 facilities, we will trace approximately 20 per facility during the pilot and *purposively* recruit approximately 10 individuals, including approximately two patients from each of the following categories: transferred; disengaged; family of dead patient. A further 2 patients who are in care at the facility at the time of study will be purposively recruited using a quasi-random selection from patient files. We will attempt to seek one male and one female from each category. We will also attempt to purposively seek 1-2 pregnant women per facility. At each of the 8 facilities, a total of approximately 10 interviews will be conducted, meaning some categories may have more than two participants. We will seek overall balance in purposive sampling across the sites. Selection will be purposive to enable gender representation and where feasible will also take consideration of age. Since pregnant women often enter HIV care through ante-natal care, their experience of care and treatment may differ from non-pregnant patients.

This sampling will seek to capture and understand the related variation. Where a potential interview candidate declines to participate, a replacement will be selected.

2. Recruitment / Tracing for Patient Interviews

Tracing procedures are unchanged from previous objectives. Recruitment of patient and family members for in-depth interviews will be based on individual permission for follow-up contact. In-depth interviews will be conducted by 1-2 trained research assistants (RAs). During the pilot stage, all traced patients will complete the standard lost to follow up forms. Then, if the participant agrees to be followed-up for an interview, the qualitative trained research assistant will visit them to conduct the interview.

3. Data Collection for Patient Interviews

Interviews will be framed by question guides developed to cover issues demonstrated in the literature to be important to understanding retention in care including: individual psycho-social and physiological factors, socio-cultural factors, geographic and financial factors and so forth. Question guides will include a section asking patients to reflect on their experiences negotiating the current health system, including the way healthcare worker attitudes, information availability, and socio-cultural norms, have affected past and current health seeking behaviour. Interview questions will be open-ended to enable RAs to probe for causal mechanisms influencing patients' engagement in care.

Interviews will be structured to elicit information about patients' perceptions, choices and behaviour in relation to their HIV care-seeking history. Interview question guides will be constructed with reference to existing literature about barriers and facilitators to retention in care in sub-Saharan Africa, but will involve iterative and open-ended discussions that enable new themes and/or experiences to be discussed. For patients who are alive and in care, we will seek to understand the barriers and facilitators of engagement. For patients alive and no longer in care or in care elsewhere, we will start with the same point of departure but expect that the balance of barriers and facilitators for these patients will differ. For patients who have died, we will interview their families to understand both the sequence of events that led up to death as well as the families' perception of the dead patient's experiences in care and their own role in their care process.

4. Procedures for Patient Interviews

We expect each IDI to last approximately 1 hour. Interviews will be conducted in the participant's choice of English or local language Nyanja, Tonga, Bemba, or Lozi. Where permission is granted, interviews will be recorded and translated (where necessary) and transcribed later. Where permission to record is not granted, and in all cases as a back-up form of data capture, extensive hand-written notes will be taken during the interview and typed up in full as soon as possible. Digital interview recordings will be stored in a secure office at CIDRZ accessible only to study staff. Transcriptions and notes will be imported into software for managing qualitative analysis (e.g. NVivo or Atlas TI) then subject to an iterative process of coding using multi-step processes of deductive and inductive techniques. After transcription, the digital recordings will be stored for a maximum of 2 years and then destroyed. Transcripts will be kept on password-protected computers and/or password-protected flash drives. The Zambia team will share the data with UNC, UAB and UCSF teams through an online file sharing system that is consistent with all three organizations' data protection policies.

Participation in the interviews will be completely voluntary and subject to written informed consent. All interviews will be conducted in a private space. Interviewers will make it clear that participants do not need to answer questions that make them

uncomfortable. Participants will receive transport reimbursement (approximately 35 ZMW per interview) as compensation for their time.

3.2.3 Focus Group Discussions

1. Sampling for FGDs

Over the course of the study we will conduct 24 focus groups – 16 during the pilot phase with the provision for a further eight during the main study. The first 16 FGDs will take place in the 8 health facilities selected for IDIs (see 3.2.2). We may conduct an additional eight FGDs (2 per clinic) in four purposively selected facilities during the main study, in order to explore themes arising from the main patient tracing exercise. In each facility, we will conduct one FGD with professional staff and one FGD with lay staff.

2. Recruitment for FGDs

Recruitment of FGD participants in all cases will be achieved through issuing open invitation to all health care workers to attend one of two FGD sessions – for professional and lay healthcare workers respectively. Separate sessions for lay and professional staff are thought necessary to enable lay staff to speak freely without the fear of contradiction by their supervisors. Recruitment will be on a first come, first served basis with a minimum of four and a maximum of 8 participants in each group.

3. Data Collection for FGDs

FGDs will be facilitated by a trained investigator using FGD discussion guides. Discussion guides will be framed by an investigation of health care workers' perception of their own role in promoting patient engagement in care and the barriers and facilitators to being able to fulfil this role. They will also include specific probes to explore any tensions between healthcare workers' perceptions and patients' own description of their experiences within health facilities. Within this framework questions will be open ended to enable the facilitator to probe emerging themes.

4. Procedures for FGDs

FGDs will last approximately 1 to 2 hours each. It is anticipated that FGDs with professional health staff will be conducted in English. FGDs with lay staff may be conducted in the participants' choice of English, or local language, Nyanja, Bemba, Tonga or Lozi. In all cases, discussions will be recorded due to the impracticality of taking sufficiently detailed notes during a multi-stakeholder discussion. Participants will be informed that they do not have to use their own name during discussions and will be issued with a participant number or pseudonym by which to identify themselves. Those who do not wish to have their voice recorded at all will be ineligible to participate.

Digital interview recordings will be stored in a password protected computer, and subsequently transferred to a secure office at CIDRZ accessible only to study staff. After transcription, the digital recordings will be stored for a maximum of 2 years and then destroyed. Transcriptions and notes will be imported into software for managing qualitative analysis (e.g. NVivo or Atlas TI) then subject to an iterative process of coding using multi-step processes of deductive and inductive techniques. All transcripts will be kept on password-protected computers and/or password-protected flash drives. The Zambia team will share the data with UNC, UAB and UCSF teams through an online file sharing system that is consistent with all three organizations' data protection policies. Participation in the FGDs will be completely voluntary and subject to written informed consent. Facilitators will make it clear that participants do not need to answer questions

that make them uncomfortable. All those taking part in an FGD will receive K50 and a refreshment for their participation.

3.2.4 Facility Audit

Recognizing the important role that health system performance plays in program retention, we will conduct direct (structured and unstructured) observations with a sample of facilities. These observations will provide an important source of data on health facility operations, service quality and responsiveness and, importantly, patient-provider relations, with which to triangulate data obtained during patient interviews and FGDs with providers.

1. Data Collection for Facility Audit

Facility audits will be conducted in all 30 facilities included in the main study. Structured observation of health centre operations will be recorded on pro-forma instruments with pre-specified items as well as space for free-text notes relating to unstructured observations. The data collection instrument will be adapted from the draft Zambian *National Healthcare Standards Assessment Tool (Class A-3 Health Facilities)* documenting, health centre compliance with various rules, processes, infrastructure standards and resource requirements, and will be designed to provide a snap-shot of the material and administrative structures in place.

2. Procedures for Facility Audit

The facility audit will be conducted by one study team member in conjunction with the facility in-charge and should take no more than 2 hours.

3.2.5 Direct Observations

Direction observations of health centre operations, formalized into research memos, will take place in the same 8 health centres selected for IDIs and FGDs, and an additional 6 facilities chosen purposively to ensure representation of every type of facility strata in the main study. A total of 14 direct observation exercises will be carried out. Data collected during observations will contribute to building a picture of typical workflows and human interactions (system software) that drive health centre operations and which might influence patients' experience and decisions relating to care-seeking. This data will also help supplement structured health centre audits through various informal conversations and fact checking opportunities.

1. Data Collection for Direct Observations

Observations will be conducted over approximately two days, incorporating time spent in each of the HIV department, outpatient department (OPD), TB corner, laboratory and pharmacy (where they pharmacy exists). Accompanied by the health centre in-charge, the trained observer(s) will introduce themselves to all staff in a general round of introductions at the beginning of the observation-period, and subsequently sit in various locations in each department, making shorthand notes related to their observations of health centre operations, healthcare worker interactions (verbal and non-verbal), patient-provider interactions and informal conversations. Notes will be structured under general thematic headings including: operational features; provider relations; patient-provider relations; environment / context. Where possible, notes will be transcribed daily into an electronic log for later analysis.

2. Procedures for Direct Observations

Direct observations will be divided into two categories. The first category of observations involves a trained study team member as a passive observer in public observable zones within the clinic, such as waiting areas, departmental registry rooms

and vitals measurement stations. Permission to be based in these areas will be established in the first instance with the overall-in charge at the commencement of study activities in the clinic. Based on a list of themes outlined in a semi-structured observation tool, the trained observer will sit or stand in an unobtrusive location to observe patient flow, one-on-one and group interactions and other elements of day-to-day operations. Each 'block' of observation will last 1-2 hours. As far as possible, the observer will not participate in conversations or activities although where necessary may answer direct questions. Short hand notes will be recorded on in summary around the following themes: (general environment and work flow; provider service behaviours; communication patterns; patient or provider group behaviours) and written up in full at the end of the day or as soon as possible thereafter.

A second category of observations will take place in the ART and OPD screening rooms. For these observations, which take place in a private setting, we will seek formal verbal consent from the health worker involved prior to commencing observations. Since the focus of the observation relates to the manner in which services are provided such as communication style and types of services being delivered we are seeking a waiver of formal consent for patients. All patients will nonetheless be informed of the observers' presence and have the option to request the observer leave. In such a case, the observer will exit the screening room for the duration of that screening event and then re-enter prior to the next patient. No personal or otherwise identifying information will be collected about either the patients or providers. Observations will be conducted once or twice in screening rooms of the OPD and ART, in 1-2 hours blocks. During this time the trained observer will adopt a completely passive role and (following consent procedures) will not participate or interact with either the patient or provider. Short hand notes based on the following themes (screening room environment; services provided; patient & provider communication styles; screening outcomes) will be recorded on a summary memo and written up in full at the end of the day or as soon as possible thereafter.

3.2.6 Analyses

All data from interviews, FGDs, observational research memos and audit tools will be typed up in full, translated (where appropriate), coded, and computerized for analysis. If conducted in a language other than English, the interviews will be translated into English with subsequent back translation or a second-party review of sections of the translation. The audio recordings, data collection tools and memos will be kept in a secure and locked file until the interviews are transcribed and the transcription is finalised, after which the recordings and notes will be destroyed. Identifying information will be removed during transcription in order to maintain confidentiality. Transcriptions will be kept in a password-protected computer file that will only be accessible to members of the research team for data analysis.

Investigators will use thematic content analysis across all data to analyse the data using qualitative software (e.g. NVivo or Atlas TI). Analysis will begin during data collection so that topics for further exploration can be noted and incorporated into ongoing fieldwork. Qualitative data analysis consists of searching for patterns in data and for conceptualizing ideas that help explain the presence of those patterns.

First round analysis of textual data to provide results to inform the tailoring (if necessary) of the larger patient survey will consist of five steps:

1. Reading for Content: Our analysis will begin with data reading until content becomes intimately familiar. As data are reviewed, emergent themes will be noted. Topics that previous research has not adequately addressed and ones that emerge unexpectedly will be explored in continued fieldwork.

2. Coding: A list of codes will be created based on identified themes and assigned to specific sections of text so that the text can be easily searched. Code definitions will be documented in a code book. Qualitative interviewers will be trained to apply the codes using qualitative analysis software. To ensure inter-coder reliability, 10% of data will be double-coded.
3. Data reduction: Once transcripts have been coded, we will work within each code to identify principal sub-themes that reflect finer distinctions in the data. This entails taking an inventory of what is related to the given code, capturing the variation or richness of each theme and noting differences between individuals or among subgroups.
4. Data display: Matrices and tables that categorize and display data will be used to help facilitate comparisons.
5. Interpretation: Once text has been read and coded, and central ideas extracted, we will identify and explain the core meanings of the data. We will search for relationships among themes or concepts identified and develop diagrams in order to map out relationships in the data.

A second and more lengthy analysis will repeat these five steps and additionally be guided by three key principles of rigorous qualitative (especially case-based) research including: i) triangulation, ii) negative case analysis and iii) respondent validation. Triangulation refers to comparing results across multiple sources of evidence and/or multiple cases to identify patterns of convergence or divergence across cases – i.e. patients [47]. Negative case analysis involves the identification of experiences or interactions that appeared to contradict the theoretical assumptions underpinning a study and the generation of rival explanations [36]. Respondent validation and peer debriefing allow for the review of preliminary case descriptions and cross-case findings, by interview or other research participants and/or other researchers. Since qualitative research is inherently reflective and recursive, this process, sometimes called 'member checking', contributes to a study's 'confirmability'. It suggests the ability to demonstrate that findings and conclusions are not just a set of subjective judgements [45, 46, 48, 49].

Once qualitatively analysed, thematically-coded and triangulated, text data may be also transformed into numeric variables and assigned binary values to indicate the presence (=1) or absence (=0) of the theme in the respondents' replies. This will allow us to: (1) examine patterns in free-text data for each of the open-ended questions, (2) examine the presence or absence of themes in individuals' responses across multiple questions, and (3) combine free-text and fixed-choice data types in various analyses.

These data will also contribute to analyses listed under Objective 3, as outlined below.

3.3 Objective 3 'Identify epidemiologic predictors of outcomes'

Research Methods

Objective 3 quantitatively analyses the data collected in Objective 1 and incorporates data and findings from Objective 2. This will be done using Directed Acyclic Graphs to clarify causal assumptions, possible bias, interaction and confounding; modelling to identify factors associated with patient outcomes and interactions between factors and outcomes; and logistic regression to examine clinic-based factors and patient outcomes.

We propose a sampling-based approach to conduct analyses to identify factors associated with mortality, retention in care, and immunologic and virologic outcomes in epidemiological analysis using multi-level regression. These analyses will provide information about variations in program effectiveness across patient, clinic and geographical variation. Individual as well as site level characteristics associated with poorer outcomes can be isolated and focus policy makers on responding to particular patient groups or

sites. The accurate identification of valid individual and program associations are a foundation for data-driven public health practice.

3.3.1 Design (Objective 3)

We will use data from Objective 1, a cohort in which we sample individuals in a complex, stratified survey design. Outcome estimates are weighted first for the probability of selection into a selected clinic sites and then, among those lost, for the probability of outcome ascertainment through tracing. We will also analyse the measurements of empathy, respect, activation and satisfaction obtained under Objective 1 in a sample of lost patients as well as group of patients in care at the same time (and therefore matched on observation time) to obtain prevalence and prevalence ratios of each of these measures. We will use the site level characteristics (e.g., provider/patient ratio, on-site TB services) obtained from the facility audits and semi-structured observations conducted under Objective 2 in multi-level regression models [25, 50] which contextualize the individual level associations. In addition, the application of a sampling based approach in the approximately 30 sites will enable quantification of between- and within- site variability in outcomes at each step of the cascade. Between-site variability in mortality, after adjustment for biological factors such as CD4 level at presentation, will provide an indication of service-delivery factors that may influence patient outcomes. We will also examine site-to-site variability through examining interactions between site characteristics and individual level associations. Finally, we leverage the considerable data around patient and provider perceptions of drivers of engagement in care derived from our mixed methods (see Objective 2) to inform the modelling process. The cutting edge of epidemiologic analysis recognizes that contextual knowledge is needed for causal inference and this contextual knowledge can best be encoded through directed acyclic graphs which influence model building in regression.

3.3.2 Patients (Objective 3)

We will use data from Objective 1, which comprise a random sample of patients enrolling in care across approximately 30 GRZ, CIDRZ-supported facilities across four Zambian provinces. This evaluation includes adults regardless of past ART initiation. This also includes patients who are already in care previous to the observation period as well as those who are newly enrolled in this interval. We will also use data from Objective 2 including site-specific measurements obtained through facility audits and semi-structured observations, as well as contextual data from patient, next-of-kin and health care provider perceptions of outcome drivers.

3.3.2 Procedures (Objective 3)

There are no field components to our study procedures in this study component. We will collate data collected from Objectives 1 and 2, as well as routinely available facility-level data, to create an analysis dataset. We will then perform the analysis as described below.

3.3.3 Measurements (Objective 3)

Measurements are conceptually unchanged from Objectives 1 and 2.

3.3.4 Analysis

1. Directed Acyclic Graphs

Our first analysis is the formal encoding of contextual knowledge obtained through qualitative interviews, observations and focus group discussions into directed acyclic graphs. This approach emerges from several observations in modern epidemiology: (1) observational analyses should strive for causal inferences; (2) statistical analyses alone provide limited inferences about causality (3) and statistical analysis must incorporate contextual knowledge both before analysis (in model specification) and after (in

interpretation) to obtain causal inferences. After using the qualitative data from Objective 2 to create initial DAGs, we will continue to revise the DAGs using the knowledge gained through modelling exposures, outcomes and variables outlined below. In this way, the DAGs can inform both the refinement of the models and the interpretation and analysis of the qualitative data. DAGs address bias by making causal assumptions explicit and provide investigators with conceptual depiction of potential bias; guidance on the control of confounding (in particular where standard regression techniques are unable to control for confounding; clarify potential for selection bias (or collider bias) via adjustment and defining the minimum set of variables on which to adjust (and thereby avoid potential additional bias by adjusting for a mediator of an unmeasured common cause) [51, 52].

2. Cox proportional hazards

Statistical analysis will first take the form of Cox Proportional hazards models in which we will seek to identify factors associated with retention, mortality and HIV RNA suppression. These analyses will be pooled as well as carried out separately for each cohort of patients: ART non-eligible (in which eligibility is treated as the event in Lusaka only), ART eligible (in which ART initiation is the event of interest) and on ART (where retention is the event of interest). We will explore models in which patient treatment status (eligibility and ART initiation) are treated as time-updated covariates in a single model. This will allow inferences to "borrow strength" by pooling larger numbers and more observation time within a single model.

3. Model specification

We explore interactions across health care level. A priori, we are interested in the impact of sex, health facility level, and CD4 level interactions. For example, we expect patients starting ART with lower CD4 values to experience more mortality than those who initiate with higher CD4 levels, but the magnitude of the difference may differ by level of health care in which patients are seeking care. It is plausible that at the hospital based clinics where there are more medical doctors and equipment, the hazard associated with starting ART with a CD4 < 50 is two-fold higher than starting with a CD4 count higher than 50, whereas among patients in lower level health centres the magnitude is larger, for example, 4 fold. This would suggest that patients with the lowest CD4 counts do better at tertiary health care centres. We will use cubic splines to assess the functional form of these relationships whenever possible to reduce residual confounding.

4. Mediation

We will also incorporate "process" metrics of site-level factors to identify mediators of facility level predictors of our major patient outcomes. For example, the time from a patient becoming eligible to receive antiretroviral drugs to the initiation of ART is an important measure of service delivery efficiency [53]; we will therefore incorporate the median time to ART initiation as a site-level characteristic in our models. We will conduct sub-group analyses evaluating time to ART initiation as the primary predictor of mortality among WHO Stage III or IV disease and HIV-TB co-infected patients. Other metrics of facility performance will include (as available) the average number of six month-monitoring CD4 counts obtained on time (among patients returning for those visits), the fraction of new patients screened for tuberculosis, and the completeness of assessment for pregnancy at each visit for women and other information collected under Objectives 1 and 2. We will look at community-level factors, defined as characteristics of patients defined in the CCP of a specific site to identify community-level mediators of patient outcome predictors such as religious influence and supportive environment. In

addition, we will explore factors associated with outcomes in the cascade and whether these factors differ across steps in the cascade.

5. Nested case control study of the effects of clinic-based patient experience

In order to assess the association between measures of clinic and socially-based patient experience and engagement in care, we will use identified “cases” – those disengaged from care – with two groups of controls: patients who are still in care – silent transfers (or patients who have sought care elsewhere) as well as patients who are still in care at their initial and original clinics. Since patients are sampled from an enumerated underlying cohort, this analysis will take the form of conditional logistic regression, which will allow odds ratios obtained from this case control study to approximate rate ratios in the absence of any rare outcome assumption. We will also examine the relationships between patient experience factors and patient return to care.

3.3.5 Sample size considerations

The projected sample size considerations given imprecision incurred by both cluster sampling as well as loss to follow up is not given by standard statistical software. However, using probability weighted dataset under an assumed measure of association with varying “N” can illustrate the effects of the sampling based approach on analytic associations.

3.4 Objective 4 ‘Understand the process of implementing a sampling-based approach to address lost to follow-up’

Research Methods

Objective 4 is an implementation science objective. It will allow the study to identify the key processes required to successfully obtain revised outcome estimates to guide future implementation. It will identify factors in the sampling-based approach that should be replicated or avoided and contextual factors that could influence success. It will also identify the perceptions of the approach and the revised estimates from key stakeholders, which will also inform any future implementation of efforts to obtain revised HIV care outcome estimates.

A recent evaluation of the evidence around global health interventions cites the gap in knowledge relating to public health practice *implementation* and recommends the use of process, cost and context assessments, among others, to fill this gap [54]. Drawing from the Proctor et al., taxonomy of implementation outcomes as a framework [55] and building upon methodologies used in other process evaluations [56], we will use a mixed methods approach to evaluate the feasibility of the sampling approach for generating regional outcome estimates and identify factors to consider for future implementation. Key pieces of implementation success of the sampling approach that this study intends to review are feasibility, fidelity (the degree to which an intervention was implemented as it was prescribed originally), cost, perception of potential for adoption and, from the study staff member perspective, acceptability, and appropriateness.

3.4.1 Design (Objective 4)

The process evaluation will include establishing a minimally sufficient activity and resource design for replication, by reviewing and analysing study activities and tracking activity-based costs. To identify potential implementation barriers and concerns, we will interview study staff and review tracking outcomes such as average time per patient tracked. We will use interviews with health care workers and health decision makers to elicit the perception of utility of the corrected estimates as well as perceptions of the tracking process for appropriateness, acceptability and potential adoption of the sampling approach. The study will also review relevant national guidance (policies, practices) to identify areas of congruence that would support or challenge future implementation.

3.4.2 Study Population (Objective 4)

Objective 4 will evaluate the study itself and engage with health care workers and decision makers to give their perceptions on sampling approach processes and outcomes. The study population consists of study staff members, routine study activity monitoring data, and health care workers and decision makers, including community members.

1. Work Plan Review

   Proposed and actual work plans for all study cadres directly related to the patient sampling and tracking will be included in the work plan review. At a minimum, this includes proposed and actual work plans from data staff members who support sampling, patient tracker supervisors, patient trackers and management staff members who oversee tracking. Routine study activity monitoring data will come from internal study activity work plans across the study staff member cadres and process data collected under Objective 1, such as persons successfully tracked and time required for tracking.

2. Study Staff Member Interviews, Debriefing sessions and Questionnaires

   The staff member interview sample will include a purposive sample of both managerial and direct implementation study staff from the 30 study sites. All tracking-related study staff members will complete process monitoring questionnaires at key stages of the study, as described below. The interviews will be guided by a semi-structured interview questionnaire. In total, we expect to conduct approximately 20 – 30 interviews. Using the same structured guide, we may also conduct debriefing sessions with multiple staff members at the same time if that is more convenient for their schedules.

<u>Inclusion Criteria for Study Staff Member Interviews</u>:

1. Employed by 'BetterInfo' study
2. Purposively selected to represent key cadres or geographical areas of interest

Exclusion Criteria for Study Staff Member Interviews:

1. Staff members under disciplinary evaluation or proceedings

3. Cost assessments

   Cost data will come from planned and actual study budget and expenditure records. Due to limitations in data access, any costs related to key study support activities (as determined by work plan review and study implementation plans) will be estimated. Additionally, the study will undertake a micro-costing exercise including estimating the average cost of obtaining the outcome for a sampled lost patient from the study perspective and the anticipated health system perspective. Unit costs will be collected from relevant sources including study and health facility data.

4. Health Care Worker and Decision Maker Key Informant Interviews

   The health care worker and decision maker interviews will include a purposive sample of key informants from across the clinics and districts/provinces where the sampling approach was implemented and high-level Zambian government health decision makers and funding partners. We will conduct approximately 20 – 30 interviews. We believe this will be sufficient to reach saturation in themes across the key informant groups of health care workers and health care decision makers. The interviews will be guided by a semi-structured interview questionnaire.

Recruitment: The study will identify key informants who represent the different health care workers who were most engaged with the sampling process (e.g. peer educators or In-Charges at clinics), community leaders and members, and decision makers from the cadre of government health officials who would contribute to decisions around adoption and future implementation of the approach (e.g. Ministry of Health or Ministry of Community Development Mother and Child Health ART and health information staff members). Once identified, a study staff member will invite the individual to participate in an interview. The invitation will be extended over email, through a phone call or in person, as appropriate. The potential participant will be free to refuse participation. If refused, the study staff members will identify a replacement key informant who they believe could provide similar perspective and invite that person to participate.

Inclusion Criteria for Key Informant Interviews:

1. Purposively selected to represent key health care worker or decision-maker cadre
2. Willing and able to give informed consent

Exclusion Criteria for Key Informant Interviews:

1. Health care workers or decision makers employed by 'BetterInfo' study

3.4.3 Procedures (Objective 4)

To establish a minimally sufficient package of activities and resources required to replicate the approach and identify key concerns to address when considering replication:

1. Work Plan Review

To understand required activities and how they might differ from expected activities, we will use work plan tracking. We will develop output based work plans for key stages of the sampling approach implementation against which progress will be tracked for study staff member cadres as described above on a regular basis. The first section of the 'Lost to Follow Up Questionnaire' which includes information about tracking times, tracking strategies and other tracking-specific implementation information will also be reviewed to better understand activity implementation.

2. Study Staff Member Interviews, Debriefing Sessions and Questionnaires

To identify barriers and key concerns about implementation of the sampling approach we will conduct semi-structured interviews, including techniques such as listing data, among a sample of study staff members working on each 'key stage' to understand the barriers and facilitators to accomplishing their required tasks including the relevance of acceptability and appropriateness as possible deductive barriers (feasibility, acceptability, appropriateness); strategies for overcoming barriers (fidelity); contextual factors that influenced their work (e.g. events, policies, infrastructure, human resources), site 'software' (e.g. ideas, interests, relationships, power dynamics and norms); or environmental factors (e.g. feasibility); and completion of a work flow diagram to map out the order and complement of activities they did to complete their task. We will also solicit their recommendations for revising the work flow activities and timing based on their experience. The interviews will be conducted at the beginning, during, toward the end, or immediately after a key stage when staff members are most experienced in their work, understand the full variation in it and will not suffer from recall bias due to a long delay between experience and data collection. If more convenient for the staff members, we may gather the information in a group setting such as a focus group discussion or a

‘debriefing session’. In the case of a debriefing session, the facilitator will produce a research memo which captures the information shared during the debrief.

Since the interviews and debriefs will contribute to both implementation monitoring in addition to research outcomes, we will use a waiver of consent for participation in the interviews. Study staff members will be reporting on the work they have accomplished and their perceptions of the challenges involved in the work. They can refuse to answer any question. The outcomes of the interviews may provide insight into staff performance. However, should a formal performance review for the purpose of human resources monitoring of organisational staff member(s) be required, it will be conducted separately from the interview described here and separate from any research aims.

The first section of the ‘Lost to Follow Up Questionnaire’ which includes information about tracking times, tracking strategies and other tracking-specific implementation information will also be used to better understand activity implementation.

3. Cost assessments

To define what is required for future implementation we will allocate the actual study costs to the activities they supported. This will include the combination of human resource (management and direct implementation) and other resources (supplies, services, etc.) required. Once a minimally sufficient set of activities for country-led implementation is defined , we will use the activity-based cost data to draft an implementation resource needs recommendations. If a critical task is identified that is not a direct expense of the study, the study staff members will estimate that expense using information from CIDRZ-affiliated ART programmes or health facilities. Once component parts of the sampling approach are understood and possible correlations within the health system are known, the study use micro-costing methodology to obtain accurate estimates of the average cost of obtaining an outcome for a lost patient.

<u>4. Health Care Worker and Decision Maker Key Informant Interviews</u>

To understand health care worker and decision maker perceptions of the subjective appropriateness of the sampling approach, we will ask questions to understand how respondents perceive ART outcomes estimates, how they interact with ART estimate data, if they see poor estimates as a problem and how they might envision using corrected estimates. The interviews will be conducted using a semi-structured interview guide. We will conduct approximately 20 – 30 interviews. To understand acceptability and potential adoption, we will review with the respondents the study process as well as minimally sufficient set of activities recommended to implement the sampling approach and understand their opinion of it, possible implementation challenges they would predict and perceived likelihood of adoption. While these data may not represent real implementation challenges they may identify possible areas of concern otherwise missed and/or represent perceptions that must be addressed to facilitate a supportive implementation environment in the future.

3.4.4 Measurements (Objective 4)

1. Work plan tracking

Using study planning and mapping tools, study leadership will identify key stages of measurement in the overall documentation of the implementation process. These may include the following : 1) LTFU list generation, 2) Paper tracing of patients on the LTFU list, 3) Identification and training of ‘tracers’, 4) Tracing of LTFU patients, 5) Data entry / management 6) Analysis. Study Management staff will evaluate work plans for

deviations and identify patterns by counting the deviations across sites. This will not only support rigorous monitoring of study progress, it will provide data to evaluate the immediate feasibility and fidelity of the approach.

2. Study Staff Member Interviews, Debriefs and Questionnaires

Interviews and debriefing sessions will last approximately 45 minutes and be facilitated using a semi-structured interview guide. The interviews and debriefing sessions will be influenced by data gathered in the questionnaires. They will be conducted by a research assistant or other study staff member and will be de-identified in order to support the study staff member to feel comfortable to comment on the entire work flow, including the interventions of their superiors.

We will seek information from the study staff about implementation facilitators, barriers and key concerns. We will review their perceptions of the relevance of acceptability and appropriateness as possible deductive barriers (feasibility, acceptability, appropriateness); strategies for overcoming barriers (fidelity); contextual factors that influenced their work such as events, policies, infrastructure, human resource, site 'software' (such as ideas, interests, relationships, power dynamics and norms) or environmental factors (feasibility). Completion of a work flow diagram will help to map out the order and complement of activities they did to complete their task. We will also solicit their recommendations for revising the workflow activities and timing based on their experience. Managerial interviews will combine queries about different key stages as required.

Tracing activity questionnaires will include items such as type of attempt (phone/in-person), number of attempts, etc. These data will be analysed using descriptive measures to determine average tracking time, most successful tracking methods and other important feasibility-related characteristics of tracking.

3. Cost assessment

Financial requirements will be assessed through regular review of budget to actual spending by activity category to assess the resources required for each activity to succeed. Expressed cost needs that cannot be met by the study funding will be documented in research memos and considered in the final cost analyses. The micro-costing will also identify costs associated with determining the outcome of a lost patient and will use sources including study data and health facility data to estimate costs.

4. Health Care Worker and Decision Maker Key Informant Interviews

Interviews will last approximately 1 hour and be facilitated using semi-structured interview guides. In-depth interviews will be conducted by 1-2 trained research assistants (RAs). The interviews with health care workers, community members and health decision makers will seek to understand the perception of utility of the corrected estimates. In addition, data will be gathered on perceptions of the tracking process for appropriateness, acceptability and potential adoption of the sampling approach among a purposively-sampled group of health care workers.

3.4.5 Analyses

1. Work plan tracking

Analysis of work plan data will include iterative coding, deductive and inductive application of themes, and quantification of incidents of activity implementation and timelines, and cross-site comparisons. This will result in a set of 'minimally sufficient' activities (i.e.

core components) required to implement the sampling methodology and operational definitions of those components. We will include environmental / contextual factors that are relevant to core component implementation and challenges / facilitators in the execution of core components. We will also identify implicit and recommended explicit performance assessment methods to monitor core component implementation.

2. Study Staff Member Interviews, Debriefs, Questionnaires and Key Informant Interviews

Interview data and debriefing memos will be analysed using grounded theory methods including iterative coding and deductive and inductive application of themes.[57] This will follow the same steps outlined in section 3.2.6, Analyses under Objective 2. Cross-site comparisons will be used to determine patterns of variation. Themes and specific recommendations from Key Informant interview data may also be compared to existing health documents including policies, guidelines, budgets, training methods and other recommendations to identify areas of concordance or deviation. Perceptions of the values of the methodology and key implementation support measures such as leadership, key competencies and organisational structures will be thematically coded. These analyses will produce context that will be considered in the core components definitions (informed by staff member data) and a report on the considerations required for potential future implementation (informed by key informant data).

3. Cost Data

Through reviewing planned and actual expenditures and cost needs, costs will be assigned to activities that comprise the core components of the study. We will review variation in activity costs across sites and produce average costs with intervals of cost variance.

All of these analyses will be used to support the production of a dissemination tool kit to support future implementation of the revised estimates methodology in other settings.

4. Process and Outcome Results Dissemination

Process and outcome results dissemination will be a critical piece of the study across all objectives. Through regular meetings at our 30 clinic sites we will regularly disseminate study implementation, interim and final findings to provincial, district, clinic and community stakeholders. We will elicit their feedback to improve the study and future dissemination plans at these meetings. In the final meeting, we will be able to share finalised site-specific revised outcome estimates.

We anticipate sharing of study interim and final process and outcomes results through communication with the Study Advisory Committee, Zambia health Technical Working Groups and specific dissemination meetings. We will prepare materials to support local and international dissemination including a ‘Revised HIV Outcome Estimate Toolkit’ that will include guides and tools that would support the implementation of the revised estimate methodology used in the study to obtain revised estimates in other settings including elsewhere in Zambia or internationally.

The study will seek other means of effective dissemination of both process and outcomes throughout the study period.

Publication of the results of this study will be governed by Ministry of Health (MOH), CIDRZ, UNC, UAB, UCSF and Gates Foundation policies. Any presentation, abstract or manuscript will be made available for MOH review prior to submission.

3.5 Considerations for Human Participants

3.5.1 Informed Consent

As indicated above, the patient tracing activities described within the BetterInfo protocol will be conducted in accordance with the standard operating procedures pertaining to current clinical practice. After these procedures are completed, the BetterInfo study protocol extends beyond current clinical practice, and patients (or, in appropriate cases, the family members of deceased patients) will then engage in an Informed Consent process to indicate their willingness to participate in the study.

This protocol, the informed consent documents, and any subsequent modifications will be reviewed and approved by the UNZA BREC and the necessary IRBs responsible for oversight of the study. Consent processes will be done in English or a selection of local languages (including Nyanja, Bemba, Tonga and Lozi). Written consent will be obtained from all patients who agree to enrol in the study. The informed consent will describe the purpose of the study, the procedures to be followed, and the risks and benefits of participation. A copy of the consent form will be offered to the study participant.

3.5.2 Risks to Participants

<u>3.5.2.1 Physical risks</u>

Staff performing blood draws will be trained in appropriate phlebotomy and will follow strict standard operating procedures for obtaining specimens. However, there is still a risk of minimal discomfort, bruising and (rarely) infection.

<u>3.5.2.2 Social risks</u>

Possible risk of involuntary disclosure of HIV status; inadvertent 'outing' of participants may occur during follow up. We will attempt to minimize this by training all follow up staff to be discreet and professional, ensuring that patient confidentiality is maintained at all times and rotating different trackers in areas so that the community members do not identify them as related to the study.

<u>3.5.2.3 Psychological</u>

Participants may become embarrassed, worried, anxious, or uncomfortable when discussing sensitive topics. The research staff will be trained to be sensitive to these areas, for example, as discussing deceased household members or challenges with HIV care.

3.5.3 Methods to Minimise Risks

We will use our previous experience with patient tracking in clinics to ensure patient confidentiality is maintained at all times. This is of critical importance to the study. Trackers will be well trained and will only state health-related purposes or other socially acceptable, non-identifiable reasons for any visits. We will also employ same gender trackers or mixed gender tracker pairs whenever possible. They will not wear any identifiable clothing or badge when making household visits. We will consult health care workers in facilities where we are working for other local considerations to ensure patient confidentiality at all times. All study staff members will be trained in appropriate implementation methods.

3.5.4 Anticipated Benefits to Participants

Participants may benefit from being followed up by trained counsellors and may decide to re-engage in care following these visits. Participants receiving additional blood tests (including CD4 and viral load testing) may receive additional medical treatment if warranted. The Zambian Ministry of Health and Ministry of Community Development Mother and Child Health may

benefit from the revised estimates allowing for better planning and formative research on drivers of outcomes potentially informing future interventions

3.5.5 Privacy of Individuals

Project staff will be trained in fundamental ethical principles and good research practices. As such, the need to respect persons and their privacy will be emphasized and shall constitute part of the Standard Operating Procedures (SOPs) for field work. All study-specific laboratory specimens, evaluation forms, reports, and other records will be identified only by a coded number to maintain participant confidentiality, unless being shared for purposes of clinical care as outlined in this protocol. All records will be kept in a secured area. All computer entry and networking programs will be done with coded numbers only. Clinical information will not be released without written permission of the participant, except as necessary for monitoring by the FDA, the Office for Human Research Protections (OHRP), the local IRB or Ethics Committee (EC).

As earlier indicated, information collected will be restricted to the study team, and if needed, the ethics review committees. Study-specific electronic databases shall not capture any individually identifying data and no unauthorised transmission of such information shall be allowed.

3.5.6 Confidentiality of Data

Completed questionnaires and all study information shall be kept confidential. All staff involved in this work will sign the CIDRZ confidentiality agreement, which prohibits them from sharing of confidential information.

3.5.7 Study Discontinuation

The study may be discontinued at any time by the sponsors, the Ministry of Health, or CIDRZ as part of their duties to ensure that research participants are protected. These data will only be used for analyses related to this study. CIDRZ and protocol-affiliated investigators and staff members will not use these data for other, unrelated analyses without the express permission of the MOH.

3.6 Data Usage

As described in Objective 1, in order to assess survival and retention in care for HIV infected patients in the 4 provinces we must (1) enumerate the entire current clinic population cohort; (2) characterize the patient population (e.g., age, sex, CD4 levels) and (3) quantify follow up and outcomes using visits and information on transfers and deaths. This will allow us to identify and seek a random sample of patients identified as lost to follow-up to ascertain their outcomes, and use these outcomes to revise estimates of mortality and retention in care in the sites and provinces.

Enumeration of the entire cohort is required to derive appropriate sampling weights. In order to enumerate the cohort we will review existing programmatic data on HIV-infected adults who have made any clinic visit in the last two years before the evaluation in the 4 provinces where CIDRZ supports the MOH: Lusaka, Western, Eastern, and Southern. This will include both new patients and any patient who has been in the clinic during the time period of interest (the previous two years).

To characterize these patients in this cohort, the study will review socio-demographic, clinical, and

laboratory variables. Again, of note, these data will include observations that begin earlier than two years prior to the evaluation even though we select patients who have made a visit in the previous two years. For example, if a patient enrolled three years ago and continues to make visits until the present, we would include them in this analysis and also desire to use their pre-therapy (or baseline) CD4 as well as values such as weight, WHO stage, visits and other information that begin three years ago.

From this enumerated population, we will we will sample 30 health facilities, identify lost patients and then seek a random sample to confirm their clinical outcomes. From each of the 30 facilities, we will randomly sample up to 225 lost patients per health facility and a total of approximately 2,000 in-care patients in Lusaka. We will work with the Ministry of Health and Ministry of Community Development and Mother-Child Health to link patient information with outcomes using the medical record number, so each individual sampled can be traced to ascertain their outcome and encourage them to re-engage in care and then to update each individual's clinical record in the electronic medical record.

These data will be shared, using secure transfer methods with all co-investigators and data-related study staff members based in Zambia and abroad. In case where export will be needed, databases will be de-identified and include only those necessary fields for QA/QC, data management, and analysis. These procedures will facilitate the monitoring of data quality and provide opportunities for local capacity building. We will seek additional, separate ethical review for any analyses falling outside the objectives of our current protocol.

4.0 Budget

Item	29-month Primary Budget
Study Staff Costs	$2,109,916
Travel	$202,990
Consulting	$39,310
Equipment and Supplies	$806,836
**Total**	**$3,159,052**

5.0 Time Frame

Month of Study	Activities
1 – 6	Study preparation, regulatory reviews
7 – 8	Staff training, site selection, sampling
9 – 11	Pilot patient tracking; in-depth interviews; initial focus group discussions; facility audits and observations
12 – 13	Initial analyses; study tool revision; additional staff training
14 – 22	Full patient tracing
23 – 25	Objective 1, 2 and 3 analyses complete; Objective 4 process review analysis complete

26 – 27	Conduct key informant interviews with health care workers and decision makers; produce dissemination toolkit
28 – 29	Provincial, national results dissemination

6.0 Reference List

1. Howard AA, Gasana M, Getahun H, Harries A, Lawn SD, Miller B*, et al.* PEPFAR Support for the Scaling Up of Collaborative TB/HIV Activities. *Jaids-Journal of Acquired Immune Deficiency Syndromes* 2012,**60**:S136-S144.

2. Atun R, Bataringaya J. Building a durable response to HIV/AIDS: implications for health systems. *J Acquir Immune Defic Syndr* 2011,**57 Suppl 2**:S91-95.

3. Amanyire G, Wanyenze R, Alamo S, Kwarisiima D, Sunday P, Sebikaari G*, et al.* Client and provider perspectives of the efficiency and quality of care in the context of rapid scale-up of antiretroviral therapy. *AIDS Patient Care STDS* 2010,**24**:719-727.

4. Bennett S, Boerma JT, Brugha R. Scaling up HIV/AIDS evaluation. *Lancet* 2006,**367**:79-82.

5. Teasdale C, Mugisha, V., Wang, C., Nuwagaba-Biribonwoha, H., Tayebwa, E., Ingarbire, E., Lahuerta, M., Sahabo, R., Abrams, E. Determinants of Mortality and Loss to Follow up among Adult Patients in Pre-ART Crae and on ART: Rwanda. In: *20th Conference on Retroviruses and Opportunistic Infections*. Atlanta; 2013.

6. Zachariah R, Tayler-Smith K, Manzi M, Massaquoi M, Mwagomba B, van Griensven J*, et al.* Retention and attrition during the preparation phase and after start of antiretroviral treatment in Thyolo, Malawi, and Kibera, Kenya: implications for programmes? *Trans R Soc Trop Med Hyg* 2011,**105**:421-430.

7. Tayler-Smith K, Zachariah R, Manzi M, Kizito W, Vandenbulcke A, Dunkley S*, et al.* Demographic characteristics and opportunistic diseases associated with attrition during preparation for antiretroviral therapy in primary health centres in Kibera, Kenya. *Trop Med Int Health* 2011,**16**:579-584.

8. Larson BA, Brennan A, McNamara L, Long L, Rosen S, Sanne I*, et al.* Early loss to follow up after enrolment in pre-ART care at a large public clinic in Johannesburg, South Africa. *Tropical Medicine & International Health* 2010,**15**:43-47.

9. Gerver SM, Chadborn TR, Ibrahim F, Vatsa B, Delpech VC, Easterbrook PJ. High rate of loss to clinical follow up among African HIV-infected patients attending a London clinic: a retrospective analysis of a clinical cohort. *J Int AIDS Soc* 2010,**13**:29.

10. Wools-Kaloustian K, Kimaiyo S, Diero L, Siika A, Sidle J, Yiannoutsos CT*, et al.* Viability and effectiveness of large-scale HIV treatment initiatives in sub-Saharan Africa: experience from western Kenya. *AIDS* 2006,**20**:41-48.

11. Rosen S, Fox MP. Retention in HIV care between testing and treatment in sub-Saharan Africa: a systematic review. *PLoS Med* 2011,**8**:e1001056.

12. Rosen S, Fox MP, Gill CJ. Patient retention in antiretroviral therapy programs in sub-Saharan Africa: a systematic review. *PLoS Med* 2007,**4**:e298.

13. Fox MP, Rosen S. Patient retention in antiretroviral therapy programs up to three years on treatment in sub-Saharan Africa, 2007-2009: systematic review. *Tropical Medicine & International Health* 2010,**15**:1-16.

14. Namusobya J, Semitala, F., Kabami, J., et al. Retention of care and Mortality among HIV - infected patients entering care with CD4 levels > 350/uL in Uganda In: *Conference on Retroviruses and Opportunistic Infections* Seattle; 2012.

15. Geng EH, Emenyonu N, Bwana MB, Glidden DV, Martin JN. Sampling-based approach to determining outcomes of patients lost to follow-up in antiretroviral therapy scale-up programs in Africa. *JAMA* 2008,**300**:506-507.

16. Geng EH, Glidden DV, Bangsberg DR, Bwana MB, Musinguzi N, Nash D*, et al.* A Causal Framework for Understanding the Effect of Losses to Follow-up on Epidemiologic Analyses in Clinic-based Cohorts: The Case of HIV-infected Patients on Antiretroviral Therapy in Africa. *Am J Epidemiol* 2012,**175**:1080-1087.

17. Namusobya J, Semitala FC, Amanyire G, Kabami J, Chamie G, Bogere J*, et al.* High Retention in Care among HIV-infected Patients Entering Care with CD4 Levels> 350/µl under Routine Program Conditions in Uganda. *Clinical infectious diseases* 2013.

18. Frangakis CE, Rubin DB. Addressing an idiosyncrasy in estimating survival curves using double sampling in the presence of self-selected right censoring. *Biometrics* 2001,**57**:333-342.

19. Proctor E, Silmere H, Raghavan R, Hovmand P, Aarons G, Bunger A*, et al.* Outcomes for implementation research: Conceptual distinctions, measurement challenges, and research agenda. *Administration and Policy in Mental Health and Mental Health Services Research* 2011,**38**:65-76.

20. Proctor EK, Landsverk J, Aarons G, Chambers D, Glisson C, Mittman B. Implementation research in mental health services: An emerging science with conceptual, methodological, and training challenges. *Administration and Policy in Mental Health and Mental Health Services Research* 2009,**36**:24-34.

21. Tassie JM, Baijal P, Vitoria MA, Alisalad A, Crowley SP, Souteyrand Y. Trends in Retention on Antiretroviral Therapy in National Programs in Low-Income and Middle-Income Countries. *J Acquir Immune Defic Syndr* 2010.

22. Namusobya J, Semitala FC, Amanyire G, Kabami J, Chamie G, Bogere J*, et al.* High Retention in Care Among HIV-Infected Patients Entering Care With CD4 Levels> 350 cells/µL Under Routine Program Conditions in Uganda. *Clinical infectious diseases* 2013,**57**:1343-1350.

23. Donabedian A. The quality of care. How can it be assessed? *Jama* 1988,**260**:1743-1748.

24. Hibbard JH, Stockard J, Mahoney ER, Tusler M. Development of the Patient Activation Measure (PAM): conceptualizing and measuring activation in patients and consumers. *Health services research* 2004,**39**:1005-1026.

25. Diez-Roux AV. Bringing context back into epidemiology: variables and fallacies in multilevel analysis. *Am J Public Health* 1998,**88**:216-222.

26. Thorpe KE, Zwarenstein M, Oxman AD, Treweek S, Furberg CD, Altman DG*, et al.* A pragmatic–explanatory continuum indicator summary (PRECIS): a tool to help trial designers. *Journal of clinical epidemiology* 2009,**62**:464-475.

27. Zwarenstein M, Treweek S, Gagnier JJ, Altman DG, Tunis S, Haynes B*, et al.* Improving the reporting of pragmatic trials: an extension of the CONSORT statement. *BMJ: British Medical Journal* 2008.

28. Roland M, Torgerson DJ. Understanding controlled trials: What are pragmatic trials? *BMJ* 1998,**316**:285.

29. Brownson RC, Colditz GA, Proctor EK. *Dissemination and implementation research in health: translating science to practice*: Oxford University Press; 2012.

30. Yu JK, Bong CN, Chen SC, Dzimadzi R, Lu DY, Makombe SD*, et al.* Outcomes in HIV-infected patients who develop tuberculosis after starting antiretroviral treatment in Malawi. *Int J Tuberc Lung Dis* 2008,**12**:692-694.

31. Brinkhof MW, Dabis F, Myer L, Bangsberg DR, Boulle A, Nash D*, et al.* Early loss of HIV-infected patients on potent antiretroviral therapy programmes in lower-income countries. *Bull World Health Organ* 2008,**86**:559-567.

32. Brinkhof MW, Pujades-Rodriguez M, Egger M. Mortality of patients lost to follow-up in antiretroviral treatment programmes in resource-limited settings: systematic review and meta-analysis. *PLoS ONE* 2009,**4**:e5790.

33. Egger M, Spycher BD, Sidle J, Weigel R, Geng EH, Fox MP*, et al.* Correcting mortality for loss to follow-up: a nomogram applied to antiretroviral treatment programmes in sub-Saharan Africa. *PLoS Med* 2011,**8**:e1000390.

34. Robins JM, Finkelstein DM. Correcting for noncompliance and dependent censoring in an AIDS Clinical Trial with inverse probability of censoring weighted (IPCW) log-rank tests. *Biometrics* 2000,**56**:779-788.

35. Kish L. Survey sampling. 1965.

36. Lumley T. Analysis of complex survey samples. *Journal of Statistical Software* 2004,**9**:1-19.

37. Clancy CM, Eisenberg JM. Outcomes research: measuring the end results of health care. *Science* 1998,**282**:245-246.

38. The Dartmouth Institute for Health Policy and Clinical Practice. Dartmouth Atlas of Health Care. In.

39. Beach MC, Keruly J, Moore RD. Is the quality of the patient-provider relationship associated with better adherence and health outcomes for patients with HIV? *J Gen Intern Med* 2006,**21**:661-665.

40. Ware NC, Wyatt MA, Geng EH, Kaaya SF, Agbaji OO, Muyindike WR*, et al.* Toward an understanding of disengagement from HIV treatment and care in sub-Saharan Africa: a qualitative study. *PLoS Med* 2013,**10**:e1001369.

41. Jewkes R, Abrahams, N., Mvo, Z. Why do nurses abuse patinets? Reflections from South African Obstectric Services. . *Soc Sci Med* 1998,**47**:1781-1788.

42. Kaplan EL, & Meier P. Nonparametric estimation from incomplete observations. *J Amer Statist Assn* 1958,**53**:457-481.

43. Coviello V, Boggess, M. Cumulative incidence estimation in the presence of competing risks. *The Stata Journal* 2004,**4**:103-112.

44. Prentice RL, Kalbfleisch JD, Peterson AV, Jr., Flournoy N, Farewell VT, Breslow NE. The analysis of failure times in the presence of competing risks. *Biometrics* 1978,**34**:541-554.

45. Gilson L. Health Policy and Systems Research: A Methodology Reader. In. Geneva: World Health Organization; 2012.

46. Yin RK. *Case Study Research: Design and Methods, Fourth Edition*. London: Sage; 2009.

47. Gilson L, Hanson K, Sheikh K, Agyepong IA, Ssengooba F, Bennett S. Building the Field of Health Policy and Systems Research: Social Science Matters. *Plos Medicine* 2011,**8**.

48. Cohen L, Manion L, Morrison KRB. Research Methods in Education. In. Abingdon: Routledge; 2007.

49. Polit DF, Hungler BP. *Nursing research. Principles and methods; 6th Edition*. Philadelphia: Lippincott; 1999.

50. Diez-Roux AV. Multilevel analysis in public health research. *Annu Rev Public Health* 2000,**21**:171-192.

51. Hernan MA, Hernandez-Diaz S, Robins JM. A structural approach to selection bias. *Epidemiology* 2004,**15**:615-625.

52. Hernan MA, Hernandez-Diaz S, Werler MM, Mitchell AA. Causal knowledge as a prerequisite for confounding evaluation: an application to birth defects epidemiology. *Am J Epidemiol* 2002,**155**:176-184.

53. Zolopa A, Andersen J, Komarow L, Sanne I, Sanchez A, Hogg E*, et al.* Early antiretroviral therapy reduces AIDS progression/death in individuals with acute opportunistic infections: a multicenter randomized strategy trial. *PLoS One* 2009,**4**:e5575.

54. Paul G. Shekelle MAM, Jill Luoto, Breanne Johnsen, Tanja R. Perry. Global Health Evidence Evaluation Framework. In. Rockville, MD: Agency for Healthcare Research and Quality; 2013.

55. Enola Proctor HS, Ramesh Raghavan, Peter Hovmand, Greg Aarons, Alicia Bunger, Richard Griffey, Melissa Hensley. Outcomes for Implementation Research: Conceptual Distinctions, Measurement Challenges, and Research Agenda. *Adm Policy Ment Health* 2011,**38**:65–76.

56. Clare IR Chandler DD, Susan Nayiga, Lilian Taaka, Christine Nabirye, Miriam Kayendeke, Eleanor Hutchinson, James Kizito, Catherine Maiteki-Sebuguzi, Moses R Kamya, Sarah G Staedke. The PROCESS study: a protocol to evaluate the implementation, mechanisms of effect and context of an intervention to enhance public health centres in Tororo, Uganda. *Implementation Science* 2013,**8**:113.

57. Hennink M, Hutter I, Bailey A. *Qualitative Research Methods*. First ed. London: SAGE Publications Ltd; 2011.
