# Supplementary material for: Understanding preferences for HIV care and treatment in Zambia: Evidence from a discrete choice experiment among patients who have been lost to follow-up
Source: PLoS Med. 2018 Aug 13;15(8):e1002636. doi: 10.1371/journal.pmed.1002636 (PMC6089406; doi:10.1371/journal.pmed.1002636)
Supplement: S2 Appendix — (PDF) [file pmed.1002636.s002.pdf]

TITLE: DCE SOP		SOP# 1.0
EFFECTIVE DATE: 19-01-2016	SUPERSEDES:	Page 1 of 4

PURPOSE

This SOP describes how to administer the DCE questionnaire on the computer tablet. Discrete choice surveys are aimed at better understanding which and to what extent various attributes of the health care environment influence re-engagement in care for ART patients.

The Discrete Choice Experiment (DCE) methodology is a quantitative research method that can measure the strength of preference and trade-offs that influence a patient's choice towards different health facility characteristics.

SCOPE

All Assistant Study Coordinators, QA/QC Data Supervisors and Tracers

RESPONSIBILITIES

I. All staff delegated to trace BetterInfo sampled LTFU patients are responsible for understanding and following this SOP.

II. The Study Coordinator is responsible for ensuring that staff are trained and that requirements of this SOP are complied with

PROCEDURES

Discrete Choice Experiments, sometimes called 'discrete choice surveys' (or DCS), present participants with two possible models of health care that differ from each other in one or more characteristics. For each pair of models, participants choose the one that they prefer.

The DCE subsets of questions aims at understanding what characteristics of ARV care respondents care the most about, and how do respondents make a trade-off.

The DCEs will be asked to a random sample of approximately 500 lost, on ART respondents only.

The eligibility criteria for the DCE is as follows:

- Refer to the protocol eligibility criteria
- Disengaged or re-engaged in care
- Lusaka province

DCE module is programmed in English, Nyanja, Bemba and Tonga. The respondent should be asked which language she/he prefers and the DCE module should be administered in that language.

The data manager has the responsibility of initiating the administration of DCE and stopping the administration of DCE when N=500 has been reached. The data manager will activate the form; he will also monitor the number of responses and delete the DCE form from the ODK as soon as the N is reached.

The surveyors should administer the DCE module if that form is active in their ODK.

For the first question:

Read the question introduction on its entirety. Make sure that the respondent understands that there will be 9 questions. Leave the respondent the opportunity to ask any question about the DCE module and ensure that they have understood.

Ensure that the tablet is visible to the respondents at all times. The respondent must be able to see what you are reading. If necessary, you can leave the tablet in the hand of the respondent as she or he reads and processes each question, and then just take it back to tap the answer that he or she selected.

After reading the introduction, the surveyor explains the logic behind each column:

The first column tells us the characteristics

Clinic A is the column on the left

Clinic B is the column on the right

For each of the questions, make sure you read out both Clinic A and Clinic B options in the following way (note, the following is an example)

"In Clinic A, the total time you spend at the clinic is 1 hour. The clinic is less than 5Km away from your home. At each visit, you are given only one month of ARVs. The clinic sees patients Mon-Friday in the mornings only. The staff at the clinic is rude"

As you read, make sure you point at every image, so that the respondent understands how you are reading the graph.

Continue:

"In Clinic B, the total time you spend at the clinic is 5 hours. The clinic is 10Km away from your home. At each visit, you are given ARVs for up to 6months. The clinic sees patients Mon-Fri mornings, as well as Saturday morning. The staff at the clinic are nice"

Then ask:

"Do you prefer going to Clinic A, Clinic B, or would you rather not go to either one, given the circumstances?

Tap the selected answer and move on.

Repeat the same process for each question. Consistency is key.

What are you allowed to do:

- Clarify differences between two models to the respondents. Example, if the respondent complains that one question looks the same as the one below, you are allowed to point out why it is different.

- You are allowed to clarify what each of the attributes mean:

- The **total time** you spend at the facility is defined as the time from the time you arrive at the facility to the time you leave the facility

- **Distance** to the facility is defined as the distance in Km from your house to the clinic

- **Months of ARV supply** is the number of months of supply of ARV that you receive at the clinic. IF you receive 3 months, it means you need to go back after 3 months to get more. If you receive 6 months, it means that you only need to go back after 6 months. If you receive 1 month, it means you need to go back the following month to pick up more drugs.

- The **opening time** is the time at which the clinic is open and receives new patients. This time is usually in the mornings only in Zambia, but some of the models specify clinics that are also seeing patients on Saturday morning—in addition to the regular hours—on afternoons also, in addition to regular hours.

Interviewers are **not allowed** to:

- Stop reading out the models. Each question needs to be read out as indicated above.

- Asking or framing questions in a different way than the one explained

- Suggesting what should respondents answer or highlight or judge certain characteristics
