# Supplementary material for: Understanding preferences for HIV care and treatment in Zambia: Evidence from a discrete choice experiment among patients who have been lost to follow-up
Source: PLoS Med. 2018 Aug 13;15(8):e1002636. doi: 10.1371/journal.pmed.1002636 (PMC6089406; doi:10.1371/journal.pmed.1002636)
Supplement: S3 Appendix — (DOCX) [file pmed.1002636.s003.docx]

**A checklist for conjoint analysis applications in health care.**

From: Bridges JFP, Hauber BA, Marshal D, Lloyd A, Prosser LA, Regier DA, et al. Conjoint Analysis Applications in Health – a Checklist: A Report of the ISPOR Good Research Practices for Conjoint Analysis Task Force. Value in Health 14 (2011): 403-413

1. Was a well-defined research question stated and is conjoint analysis an appropriate method for answering it?

1.1 Were a well-defined research question and a testable hypothesis articulated?

W*e have articulated the our research question as determining preference regarding clinic services among HIV positive people in Zambia.*

*See Background , paragraph 3*

- 1. Was the study perspective described, and was the study placed in a particular decision-making or policy context?

*We have specified that this experiment can assist in the design of differentiated care models to improve retention in HIV care.*

*See Background, paragraph 2*

What is the rationale for using conjoint analysis to answer the research question?

*We have described the limitations of other study designs for determining which interventions to prioritize for the improvement of retention in HIV care. See Background, paragraph 4*

2. Was the choice of attributes and levels supported by evidence?

2.1 Was attribute identification supported by evidence (literature reviews, focus groups, or other scientific methods)?

*We have described how literature and our qualitative research team assisted in identifying attributes and levels. See Methods, paragraph 2*

2.2 Was attribute selection justified and consistent with theory?

*As above, see Methods, paragraph 2*

2.3 Was level selection for each attribute justified by the evidence and consistent with the study perspective and hypothesis?

*As above, see Methods, paragraph 2*

3. Was the construction of tasks appropriate?

3.1 Was the number of attributes in each conjoint task justified (that is, full or partial profile)?

*Based on our qualitative work and literature review we identified these to be the main clinic attributes which may influence patients clinic attendance. We further designed experiment according to the method of Street et al and adapted the number of attributes and attribute levels according the methods outlined in Street DJ, Burgess L, Louviere JJ. Quick and easy choice sets: Constructing optimal and nearly optimal stated choice experiments. International Journal of Research in Marketing. 2005;22(4):459-70. (3)*

*See Methods, paragraph 3*

3.2 Was the number of profiles in each conjoint task justified?

*See above, Methods, paragraph 3*

3.3 Was (should) an opt-out or a status-quo alternative (be) included?

*An opt-out question was included but had poor responses and was dropped from further analyses. See Methods, paragraph 3*

4. Was the choice of experimental design justified and evaluated?

4.1 Was the choice of experimental design justified? Were alternative experimental designs considered?

*Yes, See Method, paragraph 3*

- 1. Were the properties of the experimental design evaluated?

*Yes we presented the evaluation of statistical efficiency.*

*See Methods paragraph 3 & S1 Table*

- 1. Was the number of conjoint tasks included in the data-collection instrument appropriate?

*We believe that 9 tasks per person was adequate for statistical efficiency and response efficiency, there was some evidence of response fatigue for a few patients.*

*See Results paragraph 2 & S3 Table*

5. Were preferences elicited appropriately, given the research question?

5.1 Was there sufficient motivation and explanation of conjoint tasks?

*We present the detail SOP in the S2 Appendix*

5.2 Was an appropriate elicitation format (that is, rating, ranking, or choice) used? Did (should) the elicitation format allow for indifference?

*We present the detail SOP in the S2 Appendix*

5.3 In addition to preference elicitation, did the conjoint tasks include other qualifying questions (for example, strength of preference, confidence in response, and other methods)?

*This was not included*

6. Was the data collection instrument designed appropriately?

6.1 Was appropriate respondent information collected (such as sociodemographic, attitudinal, health history or status, and treatment experience)?

*This data was already available for participants from the electronic monitoring systems*

6.2 Were the attributes and levels defined, and was any contextual information provided?

*See Methods, paragraph 2*

6.3 Was the level of burden of the data-collection instrument appropriate? Were respondents encouraged and motivated?

*See SOP in S2 appendix*

7. Was the data-collection plan appropriate?

7.1 Was the sampling strategy justified (for example, sample size, stratification, and recruitment)?

*See methods, paragraph 4 & 5*

7.2 Was the mode of administration justified and appropriate (for example, face-to-face, pen-and-paper, web-based)?

*Delivered on tablet.* *See SOP in S2 appendix*

7.3 Were ethical considerations addressed (for example, recruitment, information and/or consent, compensation)?

*Participant were asked for verbal consent to participate in the study. Those who declined are detailed in the results. The study received ethical approval from various IRB’s. Methods, paragraph 1*

8. Were statistical analyses and model estimations appropriate?

8.1 Were respondent characteristics examined and tested?

*Patient characteristics presented in S3 Table*

8.2 Was the quality of the responses examined (for example, rationality, validity, reliability)?

*Not examined*

8.3 Was model estimation conducted appropriately? Were issues of clustering and subgroups handled appropriately?

*This is presented in the methods section. We highlight that our study was not powered to be able to generate any firm conclusions from sub-group analyses.*

*See discussion, paragraph 5*

9. Were the results and conclusions valid?

9.1 Did study results reflect testable hypotheses and account for statistical uncertainty?

*We present uncertainty around all parameter estimates with 95% CI in all presented results. We further conducted several supplementary analyses to test the robustness of our results.*

*See results, paragraph 5*

9.2 Were study conclusions supported by the evidence and compared with existing findings in the literature?

*Yes*

*See discussion, paragraph 3*

9.3 Were study limitations and generalizability adequately discussed?

*See discussion, paragraph 5*

10. Was the study presentation clear, concise, and complete?

10.1 Was study importance and research context adequately motivated?

*Yes, see background*

10.2 Were the study data-collection instrument and methods described?

*Yes, see methods and S1 & S2 appendix*

10.3 Were the study implications clearly stated and understandable to a wide audience?

*Yes, see discussion, paragraph 2 and 3*
