# Supplementary material for: Understanding preferences for HIV care and treatment in Zambia: Evidence from a discrete choice experiment among patients who have been lost to follow-up
Source: PLoS Med. 2018 Aug 13;15(8):e1002636. doi: 10.1371/journal.pmed.1002636 (PMC6089406; doi:10.1371/journal.pmed.1002636)
Supplement: S1 Table — DCE, discrete choice experiment. (DOCX) [file pmed.1002636.s005.docx]

| **FULL DESIGN** | | | | | | | | | | | **Results - full design** | |
| --- | --- | --- | --- | --- | --- | --- | --- | --- | --- | --- | --- | --- |
|  |  |  |  |  |  |  |  |  |  |  |  |  |
| **Task** | **Waiting** | **Distance** | **Refill** | **Hours** | **Staff** | **Waiting** | **Distance** | **Refill** | **Hours** | **Staff** |  |  |
| **1** | 0 | 0 | 0 | 0 | 0 | 1 | 2 | 1 | 2 | 1 | 0 0 0 0 0 1 2 1 2 1 | Time taken was 0.63 seconds.  Number of choicesets: 18  Det C is: 1.30273168524e-21  Efficiency compared with optimal design for choice set size m = 2: 100.0%    Main effects are uncorrelated |
| **2** | 0 | 1 | 1 | 2 | 0 | 1 | 0 | 2 | 1 | 1 | 0 1 1 2 0 1 0 2 1 1 |  |
| **3** | 0 | 2 | 2 | 1 | 0 | 1 | 1 | 0 | 0 | 1 | 0 2 2 1 0 1 1 0 0 1 |  |
| **4** | 1 | 0 | 1 | 1 | 0 | 2 | 2 | 2 | 0 | 1 | 1 0 1 1 0 2 2 2 0 1 |  |
| **5** | 1 | 1 | 2 | 0 | 0 | 2 | 0 | 0 | 2 | 1 | 1 1 2 0 0 2 0 0 2 1 |  |
| **6** | 1 | 2 | 0 | 2 | 0 | 2 | 1 | 1 | 1 | 1 | 1 2 0 2 0 2 1 1 1 1 |  |
| **7** | 2 | 0 | 2 | 2 | 0 | 0 | 2 | 0 | 1 | 1 | 2 0 2 2 0 0 2 0 1 1 |  |
| **8** | 2 | 1 | 0 | 1 | 0 | 0 | 0 | 1 | 0 | 1 | 2 1 0 1 0 0 0 1 0 1 |  |
| **9** | 2 | 2 | 1 | 0 | 0 | 0 | 1 | 2 | 2 | 1 | 2 2 1 0 0 0 1 2 2 1 |  |
| **10** | 0 | 0 | 0 | 0 | 1 | 1 | 2 | 1 | 2 | 0 | 0 0 0 0 1 1 2 1 2 0 |  |
| **11** | 0 | 1 | 1 | 2 | 1 | 1 | 0 | 2 | 1 | 0 | 0 1 1 2 1 1 0 2 1 0 |  |
| **12** | 0 | 2 | 2 | 1 | 1 | 1 | 1 | 0 | 0 | 0 | 0 2 2 1 1 1 1 0 0 0 |  |
| **13** | 1 | 0 | 1 | 1 | 1 | 2 | 2 | 2 | 0 | 0 | 1 0 1 1 1 2 2 2 0 0 |  |
| **14** | 1 | 1 | 2 | 0 | 1 | 2 | 0 | 0 | 2 | 0 | 1 1 2 0 1 2 0 0 2 0 |  |
| **15** | 1 | 2 | 0 | 2 | 1 | 2 | 1 | 1 | 1 | 0 | 1 2 0 2 1 2 1 1 1 0 |  |
| **16** | 2 | 0 | 2 | 2 | 1 | 0 | 2 | 0 | 1 | 0 | 2 0 2 2 1 0 2 0 1 0 |  |
| **17** | 2 | 1 | 0 | 1 | 1 | 0 | 0 | 1 | 0 | 0 | 2 1 0 1 1 0 0 1 0 0 |  |
| **18** | 2 | 2 | 1 | 0 | 1 | 0 | 1 | 2 | 2 | 0 | 2 2 1 0 1 0 1 2 2 0 |  |

**S1 Table: Results of evaluation of statistical efficiency**.

From: Discrete Choice Experiments [computer software]. Sydney: School of Mathematical Sciences, University of Technology, 2007. Available from: <http://crsu.science.uts.edu.au/choice> [Accessed 2015].
