# Supplementary material for: Understanding preferences for HIV care and treatment in Zambia: Evidence from a discrete choice experiment among patients who have been lost to follow-up
Source: PLoS Med. 2018 Aug 13;15(8):e1002636. doi: 10.1371/journal.pmed.1002636 (PMC6089406; doi:10.1371/journal.pmed.1002636)
Supplement: S2 Table — (DOCX) [file pmed.1002636.s006.docx]

| **Patient Characteristics** | | **Refused DCE (N=105)** | **Accepted DCE (N=280)** |
| --- | --- | --- | --- |
|  |  | **N (%) / median (IQR)** | **N (%) / median (IQR)** |
| **Age at last visit (years)** | | 36 (29-41) | 35 (30-40) |
| **Enrolment cd4 count (cells /μl)** | | 282 (131-462) | 317 (174-494) |
| **Gender** | Female | 66 (63) | 169 (60) |
|  | Male | 39 (37) | 111 (40) |
| **ART status*** | Never started ART | 49 (37) | 126 (45) |
|  | Started ART | 56 (53) | 154 (55) |
| **WHO stage at enrolment** | Stage 1 | 44 (42) | 139 (50) |
|  | Stage 2 | 21 (20) | 40 (14) |
|  | Stage 3 | 31 (30) | 58 (21) |
|  | Stage 4 | 2 (2) | 10 (4) |
|  | Unknown | 7 (7) | 33 (12) |
| **Year of enrolment** | 2004-2006 | 5 (5) | 6 (2) |
|  | 2007-2009 | 11 (10) | 26 (9) |
|  | 2010-2012 | 28 (27) | 72 (26) |
|  | 2013-2015 | 61 (58) | 176 (63) |
| **Disclosure** | No | 4 (4) | 8 (3) |
|  | Yes | 12 (11) | 245 (88) |
|  | Unknown | 8 (8) | 27 (10) |
| **Education level** | None | 3 (3) | 9 (3) |
|  | Lower-mid basic | 19 (18) | 73 (26) |
|  | Upperbasic/secondary | 65 (62) | 147 (53) |
|  | College/Univ | 7 (7) | 19 (7) |
|  | Unknown | 0 (0) | 32 (11) |
| **Marital status** | Single | 15 (17) | 42 (18) |
|  | Married | 61 (68) | 148 (63) |
|  | Divorced | 5 (6) | 27 (11) |
|  | Widowed | 9 (10) | 19 (8) |
| **Facility** | Rural | 9 (9) | 44 (16) |
|  | Urban | 64 (61) | 203 (73) |
|  | Hospital | 32 (30) | 33 (12) |

Footnotes: * ART use at the time of sampling was 55%; by the time of the experiment 68% (N=189) had initiated ART

**S2 Table: Patient characteristics**
