# Supplementary material for: Understanding preferences for HIV care and treatment in Zambia: Evidence from a discrete choice experiment among patients who have been lost to follow-up
Source: PLoS Med. 2018 Aug 13;15(8):e1002636. doi: 10.1371/journal.pmed.1002636 (PMC6089406; doi:10.1371/journal.pmed.1002636)
Supplement: S3 Table — (DOCX) [file pmed.1002636.s007.docx]

| **Block** | **Question** | **Question: Clinic A or Clinic B** | | | | | | | | | | **Responses** | | | |
| --- | --- | --- | --- | --- | --- | --- | --- | --- | --- | --- | --- | --- | --- | --- | --- |
|  |  | **Clinic A** | | | | | **Clinic B** | | | | |  |  |  |  |
|  |  | **Waiting** | **Distance** | **Refill** | **Hours** | **Staff** | **Waiting** | **Distance** | **Refill** | **Hours** | **Staff** | **Neither**** | **Clinic A** | **Clinic B** | **Total** |
| **Block 1** | **Q1** | 1 hour | <5 Kms | 1 month | regular | rude | 3 hours | 20 Kms | 3 months | saturday | nice | 3 (2%) | 20 (12%) | 147 (86%) | 170 |
|  | **Q2** | 1 hour | 10 Kms | 3 months | saturday | rude | 3 hours | <5 Kms | 5 months | afternoon | nice | 1 (1%) | 20 (12%) | 149 (88%) | 170 |
|  | **Q3** | 1 hour | 20 Kms | 5 months | afternoon | rude | 3 hours | 10 Kms | 1 month | regular | nice | 5 (3%) | 100 (59%) | 65 (38%) | 170 |
|  | **Q4** | 3 hours | <5 Kms | 3 months | afternoon | rude | 5 hours | 20 Kms | 5 months | regular | nice | 1 (1%) | 35 (20%) | 134 (79%) | 170 |
|  | **Q5** | 3 hours | 10 Kms | 5 months | regular | rude | 5 hours | <5 Kms | 1 month | saturday | nice | 3 (2%) | 98 (58%) | 69(40%) | 170 |
|  | **Q6** | 3 hours | 20 Kms | 1 month | saturday | rude | 5 hours | 10 Kms | 3 months | afternoon | nice | 3 (2%) | 14 (8%) | 153 (90%) | 170 |
|  | **Q7** | 5 hours | <5 Kms | 5 months | saturday | rude | 1 hour | 20 Kms | 1 month | afternoon | nice | 2(1%) | 109 (64%) | 59 (35%) | 170 |
|  | **Q8** | 5 hours | 10 Kms | 1 month | afternoon | rude | 1 hour | <5 Kms | 3 months | regular | nice | 1(1%) | 9 (5%) | 160 (94%) | 170 |
|  | **Q9*** | 5 hours | 20 Kms | 3 months | regular | rude | 1 hour | 10 Kms | 5 months | saturday | nice | 4 (2%) | 10 (6%) | 153 (92%) | 167^#^ |
| **Block 2** | **Q10** | 1 hour | <5 Kms | 1 month | regular | nice | 3 hours | 20 Kms | 3 months | saturday | rude | 0 | 51 (46%) | 59(54%) | 110 |
|  | **Q11** | 1 hour | 10 Kms | 3 months | saturday | nice | 3 hours | <5 Kms | 5 months | afternoon | rude | 0 | 59 (54%) | 51 (59%) | 110 |
|  | **Q12** | 1 hour | 20 Kms | 5 months | afternoon | nice | 3 hours | 10 Kms | 1 month | regular | rude | 0 | 101 (92%) | 9 (8%) | 110 |
|  | **Q13** | 3 hours | <5 Kms | 3 months | afternoon | nice | 5 hours | 20 Kms | 5 months | regular | rude | 0 | 63 (57%) | 47(43%) | 110 |
|  | **Q14** | 3 hours | 10 Kms | 5 months | regular | nice | 5 hours | <5 Kms | 1 month | saturday | rude | 0 | 99 (90%) | 11 (10%) | 110 |
|  | **Q15** | 3 hours | 20 Kms | 1 month | saturday | nice | 5 hours | 10 Kms | 3 months | afternoon | rude | 0 | 48 (44%) | 62 (56%) | 110 |
|  | **Q16** | 5 hours | <5 Kms | 5 months | saturday | nice | 1 hour | 20 Kms | 1 month | afternoon | rude | 0 | 103 (94%) | 7 (6%) | 110 |
|  | **Q17** | 5 hours | 10 Kms | 1 month | afternoon | nice | 1 hour | <5 Kms | 3 months | regular | rude | 1 (1%) | 38 (37%) | 64 (62%) | 103^##^ |
|  | **Q18** | 5 hours | 20 Kms | 3 months | regular | nice | 1 hour | 10 Kms | 5 months | saturday | rude | 0 | 46 (45%) | 57 (55%) | 103^##^ |

*Q9= dominant question **6 participants in block 1 and 1 participant in block 2 chose the opt-out response: overall 3 participants opted out of 1 question; 2 opted out of 3 questions; 1 opted out of 5 questions; 1 opted out of all 9 questions in block 1. # 3 participants were missing data for q 9. # # 7 participants were missing data for q 17 & q18

**S3 Table: Patient responses to choice sets**
