# Supplementary material for: Understanding preferences for HIV care and treatment in Zambia: Evidence from a discrete choice experiment among patients who have been lost to follow-up
Source: PLoS Med. 2018 Aug 13;15(8):e1002636. doi: 10.1371/journal.pmed.1002636 (PMC6089406; doi:10.1371/journal.pmed.1002636)
Supplement: S4 Table — (DOCX) [file pmed.1002636.s008.docx]

| Clinic attributes | β | 95% CI | | | p-value | | Willingness to travel | | | |
| --- | --- | --- | --- | --- | --- | --- | --- | --- | --- | --- |
|  |  |  |  |  |  |  | Kilometers | | 95% CI | |
| Waiting time (per additional hr) | -0,06 | -0,08 | -0,04 | <0,001 | | 2,68 | | 1,10 | | 4,27 |
| Travel distance (per additional km) | -0,16 | -0,24 | -0,07 | <0,001 | |  | |  | |  |
| 1 vs, 3 monthly refill frequency | -3,06 | -3,72 | -2,39 | <0,001 | | 52,24 | | 33,30 | | 33,30 |
| 5 vs, 3 monthly refill frequency | 1,62 | 1,17 | 2,06 | <0,001 | | -27,65 | | -39,27 | | -16,04 |
| Extra afternoon hrs vs. regular clinic hrs | 0,01 | -0,25 | 0,26 | 0,97 | | -0,10 | | -4,46 | | 4,27 |
| Extra Saturday hrs vs. regular clinic hrs | 0,32 | 0,10 | 0,54 | 0,00 | | -5,43 | | -9,61 | | -1,25 |
| Nice vs, rude providers | 2,63 | 1,94 | 3,31 | <0,001 | | -44,94 | | -62,12 | | -27,75 |
| Constant | 0,69 | 0,14 | 1,24 | 0,01 | |  | |  | |  |
| Model specifications | Log likelihood=-827,53 ; Prob > chi2 =<0,001 ; Wald chi2 (8) =152 ; McFadden psuedo R2 = 0,31 | | | | | | | | | |

Footnotes: β = β-coefficient and represents relative utility, positive values represent positive preference; CI = confidence interval. Hr = hour. Km = kilometer. Mixed logit regression model with travel distance as a fixed effect and other attributes as random effects.

**S4 Table: Mixed Logit model and willingness to travel analysis (N=289)**
