# Supplementary material for: Understanding preferences for HIV care and treatment in Zambia: Evidence from a discrete choice experiment among patients who have been lost to follow-up
Source: PLoS Med. 2018 Aug 13;15(8):e1002636. doi: 10.1371/journal.pmed.1002636 (PMC6089406; doi:10.1371/journal.pmed.1002636)
Supplement: S5 Table — (DOCX) [file pmed.1002636.s009.docx]

| **Clinic Attributes** | **Male (N=111)** | | | | | | | **Female (N=168)** | | | | | | |
| --- | --- | --- | --- | --- | --- | --- | --- | --- | --- | --- | --- | --- | --- | --- |
|  | **β** | **95% CI** | | **p-value** | **WTW** | | | **β** | **95% CI** | | **p-value** | **WTW** | | |
|  |  |  |  |  | **Hours** | **95% CI** | |  |  |  |  | **Hours** | **95% CI** | |
| Waiting time (per additional hr) | -0,18 | -0,30 | -0,06 | 0,003 |  |  |  | -0,16 | -0,28 | -0,04 | 0,011 |  |  |  |
| Travel distance (per additional km) | -0,05 | -0,09 | -0,02 | 0,005 | 0,28 | 0,05 | 0,51 | -0,06 | -0,09 | -0,04 | <0.001 | 0,41 | 0,1 | 0,72 |
| 1 vs. 3 monthly refill frequency | -3,26 | -4,24 | -2,28 | <0.001 | 17,74 | 5,47 | 30,01 | -3,35 | -4,24 | -2,46 | <0.001 | 21,49 | 5,16 | 37,82 |
| 5 vs. 3 monthly refill frequency | 1,77 | 1,04 | 2,51 | <0.001 | -9,70 | -17,11 | -2,2 | 1,78 | 1,20 | 2,35 | <0.001 | -11,40 | -20,77 | -2,02 |
| Extra afternoon hrs vs. regular hrs | -0,06 | -0,43 | 0,31 | 0,756 | 0,32 | -1,74 | 2,37 | 0,16 | -0,16 | 0,48 | <0.001 | -1,04 | -3,4 | 1,33 |
| Extra Saturday hrs vs. regular hrs | 0,18 | -0,17 | 0,54 | 0,308 | -1,00 | -2,94 | 0,93 | 0,35 | 0,08 | 0,62 | 0,323 | -2,26 | -4,64 | 0,13 |
| Nice vs. rude providers | 3,18 | 2,13 | 4,23 | 0 | -17,32 | -29,99 | -4,67 | 2,58 | 1,47 | 3,68 | 0,011 | -16,54 | -29,14 | -3,93 |
| Constant | 0,33 | -0,35 | 1,01 | 0,34 |  |  |  | 0,61 | -0,19 | 1,41 | 0,135 |  |  |  |
|  | Log likelihood= -575.188; Prob > chi2 = 0.000; Wald chi2 (8) = 91.82; McFadden psuedo R^2^ = 0.37 | | | | | | | Log likelihood= -836.973; Prob > chi2 = 0.000; Wald chi2 (10) = 158.66; McFadden psuedo R^2^ = 0.36 | | | | | | |

β = β-coefficient and represents relative utility, positive values represent positive preference; CI = confidence interval; WTW=Willingness to wait calculated as β-attribute/ β-waiting time.

**S5 Table: Mixed logit model and willingness to wait, by gender**
