# Supplementary material for: Understanding preferences for HIV care and treatment in Zambia: Evidence from a discrete choice experiment among patients who have been lost to follow-up
Source: PLoS Med. 2018 Aug 13;15(8):e1002636. doi: 10.1371/journal.pmed.1002636 (PMC6089406; doi:10.1371/journal.pmed.1002636)
Supplement: S6 Table — (DOCX) [file pmed.1002636.s010.docx]

| **Clinic attributes** | **β** | **95% Confidence Interval** | | **p-value** |
| --- | --- | --- | --- | --- |
| Waiting time (per additional hr) | -0,15 | -0,25 | 1,52 | 0,004 |
| Travel distance (per additional km) | -0,04 | -0,06 | -0,05 | <0.001 |
| 1 vs. 3 monthly refill frequency | -3,17 | -4,02 | -0,02 | <0.001 |
| 5 vs. 3 monthly refill frequency | 1,28 | 0,82 | -2,32 | <0.001 |
| Extra evening hrs vs. regular hrs | 0,02 | -0,25 | 1,75 | 0,883 |
| Extra Saturday hrs vs. regular hrs | 0,28 | 0,02 | 0,29 | 0,038 |
| Nice vs. rude providers | 2,25 | 0,92 | 3,58 | 0,001 |
| Constant | 0,67 | -0,19 | 0,55 | 0,127 |
| Model specifications | Log likelihood= -575.188; Prob > chi2 = 0.000; Wald chi2 (8) = 91.82; McFadden psuedo R2 = 0.36 | | | |

β = β-coefficient and represents mean utility, positive values represent positive preference; CI = confidence interval

**S6 Table: Mixed logit model, among ART users (N=189)**
