# Supplementary material for: Understanding preferences for HIV care and treatment in Zambia: Evidence from a discrete choice experiment among patients who have been lost to follow-up
Source: PLoS Med. 2018 Aug 13;15(8):e1002636. doi: 10.1371/journal.pmed.1002636 (PMC6089406; doi:10.1371/journal.pmed.1002636)
Supplement: S7 Table — (DOCX) [file pmed.1002636.s011.docx]

| **Clinic Attributes** | **Disengaged (N=169)** | | | | **Re-engaged (N=110)** | | | |
| --- | --- | --- | --- | --- | --- | --- | --- | --- |
|  | **Coefficient** | **95% CI** | | **p-value** | **Coefficient** | **95% CI** | | **p-value** |
|  |  | **Lower bound** | **Upper bound** |  |  | **Lower bound** | **Upper bound** |  |
| Waiting time (per additional hr) | -0,06 | -0,17 | 0,05 | 0,292 | -0,33 | -0,51 | -0,15 | <0.001 |
| Travel distance (per additional km) | -0,04 | -0,07 | -0,02 | <0.001 | -0,07 | -0,11 | -0,03 | <0.001 |
| 1 vs. 3 monthly refill frequency | -3,50 | -4,59 | -2,42 | <0.001 | -3,40 | -4,54 | -2,26 | <0.001 |
| 5 vs. 3 monthly refill frequency | 1,68 | 1,17 | 2,18 | <0.001 | 1,91 | 0,57 | 3,25 | 0,005 |
| Extra evening hrs vs. regular clinic hrs | -0,07 | -0,38 | 0,24 | 0,65 | 0,29 | -0,19 | 0,78 | 0,24 |
| Extra Saturday hrs vs. regular clinic hrs | 0,31 | 0,03 | 0,60 | 0,032 | 0,42 | -0,06 | 0,90 | 0,089 |
| Nice vs. rude providers | 2,44 | 1,54 | 3,33 | <0.001 | 3,74 | 1,85 | 5,64 | <0.001 |
| Constant | 1,12 | 0,42 | 1,81 | 0,002 | -0,93 | -2,26 | 0,41 | 0,173 |
| Model specifications | Log likelihood= -490.776; Prob > chi2 = 0.000; Wald chi2 (8) = 87.74; McFadden psuedo R2 = 0.36 | | | | Log likelihood= -313.080; Prob > chi2 = 0.000; Wald chi2 (8) = 53.34; McFadden psuedo R2 = 0.35 | | | |

Footnotes: β = β-coefficient and represents relative utility, positive values represent positive preference; CI = confidence interval; McFadden pseudo R^2^ = 1-(e(ll)/e(ll_0)).

**S7 Table: Mixed logit model by engagement status**
