# Supplementary material for: Understanding preferences for HIV care and treatment in Zambia: Evidence from a discrete choice experiment among patients who have been lost to follow-up
Source: PLoS Med. 2018 Aug 13;15(8):e1002636. doi: 10.1371/journal.pmed.1002636 (PMC6089406; doi:10.1371/journal.pmed.1002636)
Supplement: S8 Table — (DOCX) [file pmed.1002636.s012.docx]

| **Clinic Attributes** | **Rural (N=44)** | | | | **Urban (N= 203)** | | | | **Hospital (N=33)** | | | |
| --- | --- | --- | --- | --- | --- | --- | --- | --- | --- | --- | --- | --- |
|  | **Coef** | **95% CI** | | **p-value** | **Coef** | **95% CI** | | **p-value** | **Coef** | **95% CI** | | **p-value** |
|  |  |  |  |  |  |  |  |  |  |  |  |  |
| Waiting time (per additional hr) | -0,53 | -0,85 | -0,21 | 0,001 | -0,09 | -0,18 | 0,01 | <0.001 | -0,14 | -0,59 | 0,31 | 0,539 |
| Travel distance (per additional km) | -0,14 | -0,23 | -0,05 | 0,002 | -0,04 | -0,07 | -0,02 | 0,066 | -0,06 | -0,13 | 0,01 | 0,118 |
| 1 vs. 3 monthly refill frequency | -5,78 | -11,30 | -0,25 | 0,041 | -3,07 | -3,74 | -2,40 | <0.001 | -4,29 | -13,23 | 4,65 | 0,347 |
| 5 vs. 3 monthly refill frequency | 1,84 | -0,42 | 4,10 | 0,111 | 1,80 | 1,32 | 2,29 | <0.001 | 1,52 | -0,08 | 3,12 | 0,062 |
| Extra afternoon hrs vs. regular clinic hrs | -0,12 | -0,96 | 0,71 | 0,772 | 0,08 | -0,21 | 0,37 | <0.001 | 0,00 | -1,31 | 1,31 | 0,998 |
| Extra Saturday hrs vs. regular clinic hrs | 0,55 | -0,08 | 1,17 | 0,086 | 0,28 | 0,01 | 0,55 | 0,577 | 0,18 | -1,30 | 1,66 | 0,811 |
| Nice vs. rude providers | 4,84 | 0,54 | 9,15 | 0,027 | 2,25 | 1,61 | 2,88 | 0,046 | 5,28 | -4,63 | 15,19 | 0,297 |
| Constant | -0,05 | -0,93 | 0,83 | 0,912 | 1,07 | 0,48 | 1,66 | <0.001 | 0,82 | -0,58 | 2,23 | 0,253 |
| Model specifications | Log likelihood= -121.48; Prob > chi^2^ = 0.0375; Wald chi^2^ (8) = 26.36; McFadden psuedo R^2^ = 0.37 | | | | Log likelihood= -594.86; Prob > chi^2^ = 0.000; Wald chi^2^ (8) =116.63; McFadden psuedo R^2^ = 0.36 | | | | Log likelihood= -90.29; Prob > chi2 = 0.010; Wald chi2 (8) = 20.24; McFadden psuedo R2 = 0.34 | | | |

Footnotes: β = β-coefficient and represents mean utility, positive values represent positive preference; CI = confidence interval; McFadden pseudo R^2^ = 1-(e(ll)/e(ll_0)).

**S8 Table: Mixed logit model, by health care setting**
