# Supplementary material for: Understanding preferences for HIV care and treatment in Zambia: Evidence from a discrete choice experiment among patients who have been lost to follow-up
Source: PLoS Med. 2018 Aug 13;15(8):e1002636. doi: 10.1371/journal.pmed.1002636 (PMC6089406; doi:10.1371/journal.pmed.1002636)
Supplement: S9 Table — (DOCX) [file pmed.1002636.s013.docx]

| **Clinic attributes** | **Coefficient** | **95% Confidence Interval** | | **p-value** |
| --- | --- | --- | --- | --- |
|  |  |  |  |  |
| Waiting time (per additional hr) | -0.14 | -0.23 | -0.06 | <0.001 |
| Travel distance (per additional km) | -0.05 | -0.07 | -0.03 | <0.001 |
| 1 vs. 3 monthly refill frequency | -3.36 | -4.05 | -2.68 | <0.001 |
| 5 vs. 3 monthly refill frequency | 1.62 | 1.15 | 2.09 | <0.001 |
| Extra afternoon hrs vs. regular clinic hrs | 0.04 | -0.21 | 0.28 | 0.766 |
| Extra Saturday hrs vs. regular clinic hrs | 0.17 | -0.05 | 0.40 | <0.001 |
| Nice vs. rude providers | 2.69 | 1.92 | 3.45 | <0.001 |
| Constant | 0.77 | 0.06 | 1.48 | 0.034 |
|  | Log likelihood= -760.30; Prob > chi2 = 0.000; Wald chi2 (8) = 112.09; McFadden psuedo R2 = 0.36 | | | |

**S9 Table: Mixed logit model; restricted to complete choice sets (N=262)**
